# Minimal biomass deposition in banded iron formations inferred from organic matter and clay relationships

**DOI:** 10.1038/s41467-019-12975-z

**Published:** 2019-11-04

**Authors:** Matthew S. Dodd, Dominic Papineau, Franco Pirajno, Yusheng Wan, Juha A. Karhu

**Affiliations:** 1 London Centre for Nanotechnology, University College London, WC1H 0AH London, UK; 2 Department of Earth Sciences, University College London, WC1E 6BT London, UK; 3 Centre for Planetary Sciences, University College London, WC1E 6BT London, UK; 4 School of Earth Sciences & State Key Laboratory of Biogeology and Environmental Geology, China University of Geosciences, Wuhan, China; 5 Centre for Exploration Targeting, The University of Western Australia, 35 Stirling Highway, Crawley, WA6009 Australia; 6 Beijing SHRIMP Centre, Institute of Geology, Chinese Academy of Geological Sciences, Beijing, China; 7 Department of Geosciences and Geography, University of Helsinki, Helsinki, P.O. Box 64, Finland; 8 State Key Laboratory of Biogeology and Environmental Geology, China University of Geosciences, Wuhan, China

**Keywords:** Palaeoecology, Biogeochemistry, Carbon cycle, Element cycles, Astrobiology

## Abstract

The cycling of iron and organic matter (OM) is thought to have been a major biogeochemical cycle in the early ferruginous oceans which contributed to the deposition of banded iron formations (BIF). However, BIF are deficient in OM, which is postulated to be the result of near-complete oxidation of OM during iron reduction. We test this idea by documenting the prevalence of OM in clays within BIF and clays in shales associated with BIF. We find in shales >80% of OM occurs in clays, but <1% occurs in clays within BIF. Instead, in BIF OM occurs with ^13^C-depleted carbonate and apatite, implying OM oxidation occurred. Conversely, BIF which possess primary clays would be expected to preserve OM in clays, yet this is not seen. This implies OM deposition in silicate-bearing BIF would have been minimal, this consequently stifled iron-cycling and primary productivity through the retention of nutrients in the sediments.

## Abstract (web summary)

Banded iron formations could have formed in the early oceans due to microbial metabolism. Here Dodd and colleagues find little organic carbon in these formations, indicating microbial iron cycling was minimal and could have limited the recycling of important nutrients to overlying waters.

## Introduction

Banded iron formations (BIF) are layered, iron-rich (15–40% iron) sediments which were laid down during the Precambrian. It is purported that they formed through a complex interplay of various microbial metabolisms. These involved either photoferrotrophy^1^, or/ in addition to dissimilatory iron-reduction^2,3^. Paradoxically, BIF are characteristically lacking in OM with total organic carbon (TOC), typically around 0.01 wt%^4^. An ad hoc hypothesis used to account for these low organic totals, suggests there was near-complete respiration/oxidation of OM, coupled to ferric iron reduction^2,3^. Proposed evidence for OM oxidation in BIF includes, ^13^C-depleted carbonate suggested to have formed after ^13^C-depleted OM^3^, and ^56^Fe depletions proposed to represent microbial iron reduction^3,5^. The co-occurrence of siderite and magnetite with haematite, apatite, carbonate and OM is then the hypothesised result of OM oxidation and iron reduction.

BIF often contain layers of nanoscopic particles of Fe-silicates and iron-oxyhydroxides^4,6^, and therefore would have high mineral surface areas. This is noteworthy because OM in marine sediment is concentrated with higher mineral surface areas, hence inversely proportional with grain size^7^. Other factors such as depositional rate, redox conditions and OM chemistry also will affect OM preservation in sediments^8^. The ratio of OM to mineral surface area is relatively constant as sediments age, indicating that OM loss over time is minimised by the available mineral surface area^9^. This implies that mineral-bound OM is effectively protected from oxidation, whereas unbound OM has been shown to be oxidised within days by microbes. This preservative mechanism may be so effective it preserves OM in oxygenated marine sediments for 10 s of millions of years and more^9,10^. More than 90% of OM preserved in marine sediments is intimately associated with mineral surfaces^9^, forming organo-minerals. OM is rapidly bound to specific minerals, such as clays in the water column, and specifically through bonds between functional groups and mineral surfaces^11^. Clay minerals represent some of the most common minerals associated with OM, and in cohort with other minerals can hinder^12^ and slow the decay rate of OM by five orders of magnitude^9^. This is thought to be achieved either though adsorption of OM to mineral surfaces, or entombment of OM within pore spaces in clays, which are inaccessible to enzymes used by microbes to break down OM^12^.

OM, or graphitic carbon as its metamorphosed equivalent, is therefore expected to survive as organo-minerals in the geological record. Organo-minerals can consequently be used to reveal whether the low organic totals of BIF are a result of restricted deposition of OM in BIF, or near-complete oxidation of the initial amount. We therefore document and compare OM mineral relations in OM-rich and OM-poor lithologies, using a suite of metapelite and BIF ranging in age from 3700 to 1800 million years old. We find that clays in BIF are devoid of OM, whereas >80% of OM in metapelite occurs with clays. Since clays are expected to preserve OM, it is suggested OM deposition was minimal in clay-bearing BIF, however OM is also found associated with apatite and ^13^C-depleted carbonate in BIF, suggesting OM oxidation may have occurred.

## Results

### Analysis of OM in metapelites

**Fig. 1:**
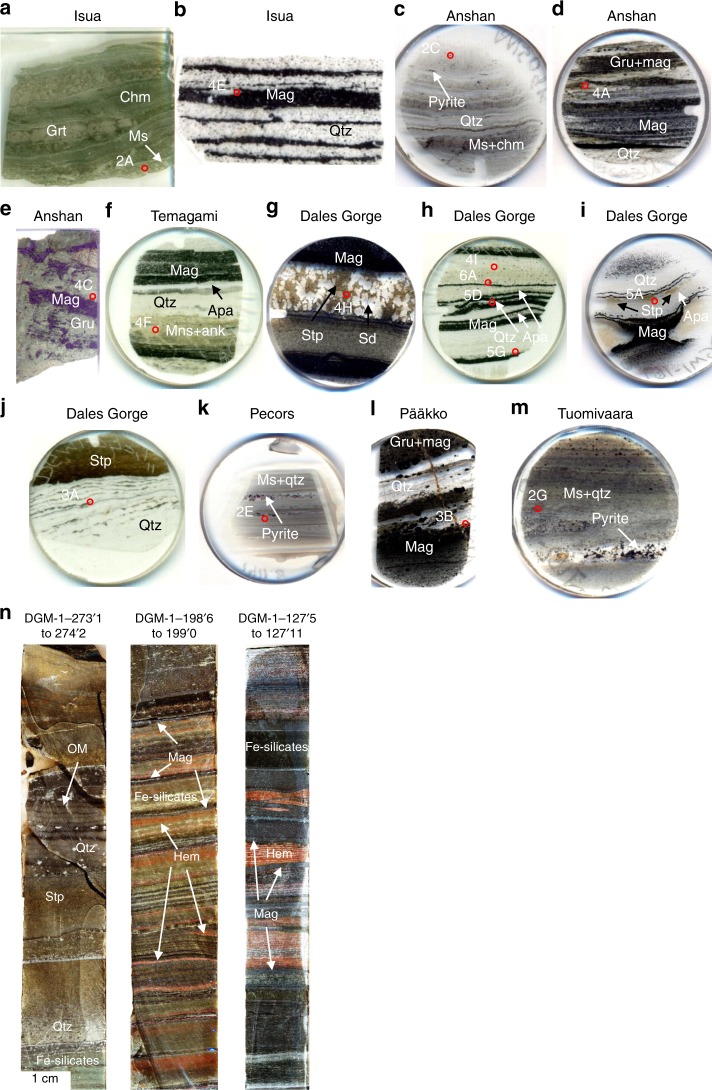
Images of thin sections of samples used in this study. **a** Isua metapelite (GRDP0402). **b** Isua BIF (GRDP0404). **c** Anshan metapelite (ANS0904). **d** Anshan BIF (ANS0917). **e** Anshan BIF (ANS1507). **f** Temagami BIF (TE0704). **g** Dales Gorge BIF (DGM-1-57‘8”). **h** Dales Gorge BIF (DGM-1-198’6”). **i** Dales Gorge BIF (DGM-1-169'6”). **j** Ferruginous chert, Dales Gorge section (DGM-1-273’−10”). **k** Pecors metapelite (SM0175_1383.5). **l** Pääkkö sulphidic iron-formation (RPK344-156.4). **m** Tuomivaara metapelite (TU2-98.25). **n** Polished drill cores from the Brockman BIF, note the relative variability in mineralogy between core sections, representing variable depositional conditions. Numbered circles correspond to figure panels. Circle sections are 2.5 cm wide, rectangular sections are 8 cm long apart from the Isua section which is 4 cm long. *Ank* ankerite, *Apa* apatite, *Chm* chamosite, *Gru* grunerite, *Grt* grunerite, *Mag* magnetite, *Ms* muscovite, *Qtz* quartz, *Sd* siderite, *Stp* stilpnomelane

**Fig. 2:**
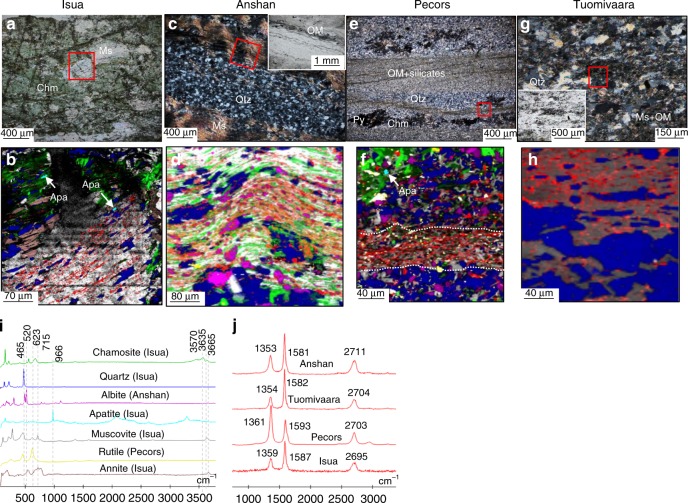
Organic matter (OM) mineral assemblages in metapelites. **a** Transmitted light (TL) image of muscovite in the Isua metapelite, exhibiting parallel-aligned graphite inclusions. **b** Raman image of the boxed area in A, showing the concentration of graphite in muscovite. **c** Cross-polar image muscovite layers in the Anshan metapelite, inset is a TL image of the same area showing black particles of graphite in muscovite layers. **d** Raman image of the boxed area in C, showing graphite within muscovite and chamosite layers with dispersed grains of albite. **e** Cross-polar image of OM and silicate layers in the Pecors metapelite, with sulphide-chamosite clusters. **f** Raman image of the boxed area in E, showing the concentration of OM with muscovite in a matrix of quartz and albite. **g** CP image of muscovite in quartz from the Tuomivaara metapelite, inset is of the same image in TL, dark particles are graphite. **h** Raman image of the boxed area in G, showing graphite within muscovite. **i** Representative Raman spectra for this figure (Sample names refer to where spectra taken from). **j** Raman spectra for organic matter in the metapelites of this figure

In the Isua metapelite (Supplementary Notes 1; Table 1), muscovite forms sheets up to 100 s of microns in width, distributed as folded layers that define schistosity in sedimentary bedding (Fig. 1a). Other phyllosilicates include chamosite and biotite (Supplementary Table 2). Black particles of graphite form linear disseminations throughout the muscovite (Fig. 2a, b) and are disseminated as microscopic particles in bands parallel to the foliated sedimentary laminations. Small apatite grains up to 10 µm in length occur in the muscovite and other phyllosilicates (Fig. 2b). Graphite and apatite also occur outside muscovite grains, but in considerably lower abundance. In the Anshan metapelite, quartz forms the majority of the ground mass, and in plane polarised light, graphite forms dark, continuous, undulating layers (Figs. 1c and 2c). In cross-polars, graphite layers can be seen correspondingly following the muscovite layers (Fig. 2c). Raman mapping shows graphite occurs almost entirely within layers of muscovite and chamosite, with inclusions of feldspar (Fig. 2d; Supplementary Table 2). Within the Pecors metapelite, OM, muscovite and albite form layers in the quartz matrix, similar to the Anshan metapelite (Fig. 2e, f). Rutile is also found to be concentrated in the OM layers within the Pecors metapelite (Fig. 2f). The Tuomivaara metapelite consists of a recrystallised quartz matrix with muscovite (Fig. 2g; Supplementary Table 2) forming irregular grains dispersed between quartz grains. The muscovite is typically dark in colour from the myriad of graphite inclusions (Fig. 2g, h).

The δ^13^C values of graphitic carbon in the Isua, Anshan and Tuomivaara metapelite samples are −21.8‰, −23.4‰, and −23.8‰, while δ^13^C_org_ for the Pecors pelite averages −34.6‰ (Supplementary Table 3). The Raman spectra of graphitic carbon in the various metapelite samples yield crystallisation temperatures consistent with their respective metamorphic grades, implying the graphitic carbon is a syngenetic and prograde phase (Supplementary Table 4). Carbonate constitutes a minor component of the pelite samples.

### Analysis of OM in ferruginous cherts

**Fig. 3:**
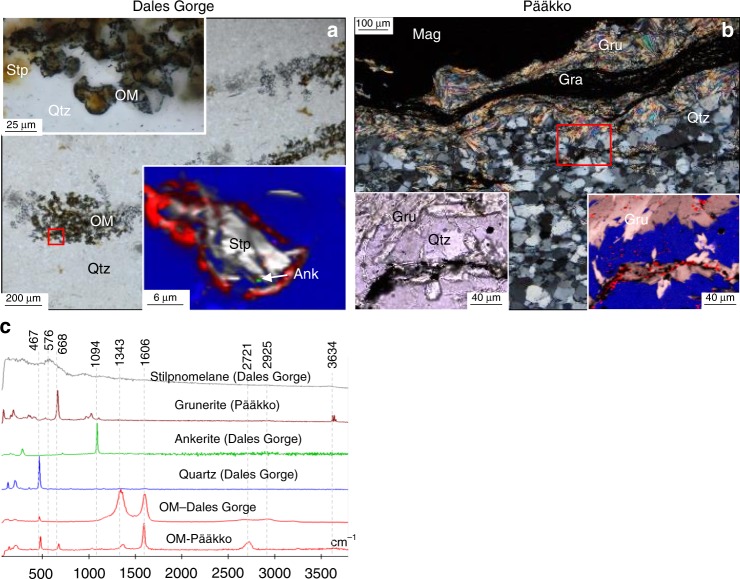
Organic matter in iron-clays from ferruginous chert. **a** TL image of organic-matter and stilpnomelane layers in an Fe-rich chert from the Dales Gorge formation. Top-left inset is a TL image of the boxed area, and top-right inset a Raman image of the boxed area, showing organic matter around stilpnomelane. **b** Cross-polar image of graphite within grunerite layers in the sulphidic Pääkkö iron-formation. Bottom left inset is a TL image of the boxed area and bottom right inset is a Raman image of the boxed area showing graphite occurring predominantly within prograde grunerite. **c** Representative Raman spectra for this figure

In ferruginous chert layers of >10 cm thickness within the Dales Gorge BIF sequence, carbonate and stilpnomelane form layers rich in OM (Fig. 1n; 3a). These cherty layers are distinct from the typical BIF layers in the Dales Gorge sequence, and can be described as ferruginous chert, as they are composed only of chert + stilpnomelane + ankerite + OM, no iron-oxides are preserved within this layer. The OM exhibits sub-spherical morphologies, with stilpnomelane at their centres (Fig. 3a). Stilpnomelane also occurs outside of the spherical clusters, but always directly associated with the organic-rich layers. Similarly, in an organic-rich (C_org_ – 1.5 wt%) (Supplementary Tables 2 and 5), sulphidic, feldspar-bearing iron-formation from the Pääkkö Formation of central Finland, graphite is found to be concentrated in some grunerite bands, which form interlayers between magnetite and quartz bands (Fig. 3b). The graphite forms undulating layers within grunerite bands, and only a minor amount of microscopic graphite particles is found in the quartz matrix. The bulk rock δ^13^C_Carb_ and δ^13^C_org_ for the ferruginous chert from the Dales Gorge BIF are −8.0‰ and −24.0‰, respectively, and the δ^13^C_Carb_ and δ^13^C_org_ values for the Pääkkö iron-formation are −11.7‰ and −19.6‰, respectively (Supplementary Table 3).

### Analysis of OM in banded iron formations

**Fig. 4:**
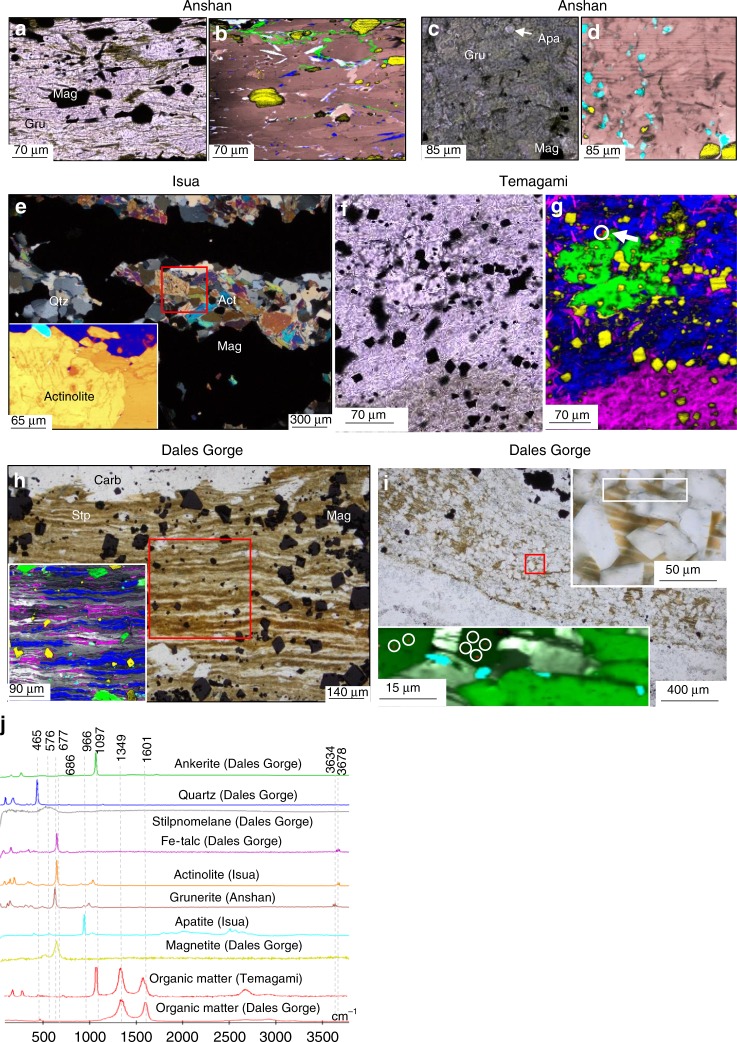
Iron-clays lacking organic matter in BIF. **a** TL image of a grunerite layer in the Anshan BIF. **b** Raman map of A, showing no inclusions within the grunerite, and large grains of magnetite, quartz and ankerite between grunerite. White colours are an artefact from colour mixing. **c** TL image of grunerite with apatite inclusions in the Anshan BIF. **d** Raman image of C, showing apatite inclusions in grunerite with no graphite inclusions. **e** Cross-polar image of grunerite and magnetite layers in the Isua BIF, inset is a Raman image of the boxed area, showing grunerite and apatite with no inclusions of graphite. **f** TL image of ankerite and Fe-talc in Temagami BIF. **g** Raman image of ankerite and Fe-talc in panel **f**, organic matter occurs only with the carbonate (circled). **h** TL image of finely layered stilpnomelane with magnetite, and ankerite overgrowths in the Dales Gorge BIF, inset is a Raman image of the boxed area, showing apatite within and around the stilpnomelane layers, neither the stilpnomelane or apatite have organic matter inclusions. **i** TL image of laminated stilpnomelane layers, overgrown by siderite rhombohedra. Top right inset is a higher magnification image of the red boxed area, white box in the inset corresponds to the bottom right inset. The bottom left inset is a Raman image showing the occurrence of organic matter (circles) in carbonate, and not stilpnomelane. **j** Representative Raman spectra for this figure

Grunerite (Supplementary Table 2) in the Anshan BIF samples forms millimetre thick layers associated with quartz and magnetite layers (Fig. 4a). In some samples, grunerite comprises the bulk of the sample, alternating with magnetite layers throughout (Fig. 4b). Neither grunerite, nor apatite in grunerite from the Anshan samples was found to host or be associated with graphite (Fig. 4a–d). The Isua BIF similarly comprises magnetite bands in a quartz matrix, with layers of actinolite (Supplementary Table 2). Raman scans of actinolite layers in the Isua BIF also show no inclusions of graphite (Fig. 4e). Bulk rock δ^13^C_Carb_ value of the Anshan BIF is −8.2‰, whereas δ^13^C_org_ values for the Anshan and Isua BIF are −26.7‰ and −26.9‰, respectively (Supplementary Table 3). Bulk rock geochemical data for the Isua and published data for the Anshan BIF are reported in Supplementary Table 4.

In the Temagami BIF, ankerite co-occurs with microscopic particles of OM, yet the surrounding Fe-talc (minnesotaite) does not host any OM (Fig. 4f, g). OM also occurs in ankerite and apatite intergrowths within an apatite-rich layer up to 600 microns in thickness^13^. Bulk rock δ^13^C_Carb_ and δ^13^C_org_ values for the Temagami BIF sample are −4.6‰ and −27.8‰ to −32.8‰ respectively (Supplementary Table 2). Bulk rock geochemical data for the Temagami BIF are reported in Supplementary Table 4.

**Fig. 5:**
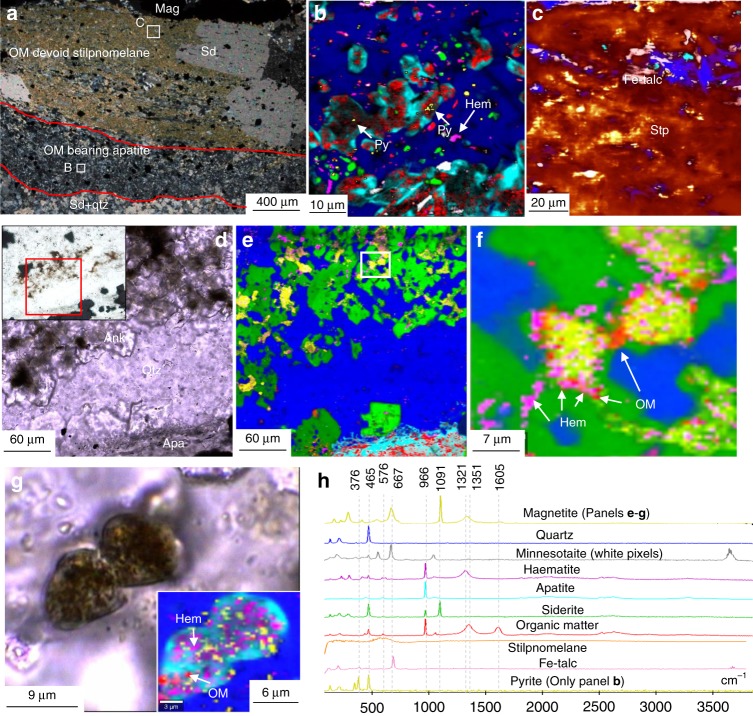
Organic matter, apatite and carbonate layers in BIF. **a** CP image of a laminated OM-devoid Fe-silicate layer and OM-bearing apatite layer adjacent to a magnetite layer. **b** Raman image of the boxed area in **a**; organic matter occurs within apatite, as does pyrite (white arrows). **c** Raman image of boxed area in **a**; no organic matter was found within the stilpnomelane. The yellow colours are artefacts, no pyrite was identified in this scan area. **d** TL image of ankerite and apatite between magnetite bands. **e** Raman image of **d**, showing pockets of magnetite and haematite in ankerite. **f** Higher magnification Raman image of the boxed area in **e**, showing the close spatial association of organic matter, haematite and magnetite within the ankerite. **g** TL image of an apatite grain with numerous inclusions, inset is a Raman image of the apatite, the inclusions are organic matter, haematite and magnetite. **f** Representative Raman spectra for this figure

**Fig. 6:**
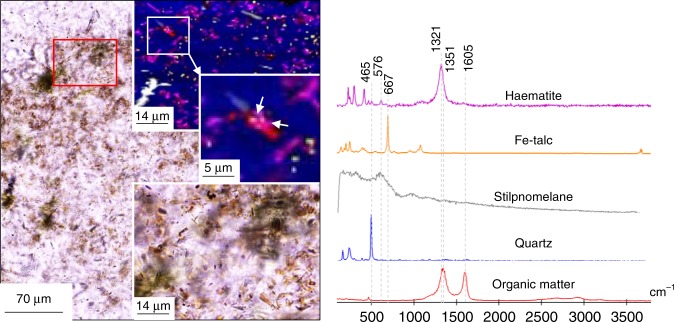
OM associated with haematite in the Dales gorge BIF. **a** TL image of haematite layer in the Dales gorge BIF. Bottom right inset is a TL image of the red boxed area. Top right inset is a Raman map of the red boxed area. Middle right inset is a Raman map of the white boxed area. White arrows point to haematite bound with organic matter. **b** Representative Raman spectra for the this figure

Stilpnomelane (Supplementary Table 2) forms continuous parallel layers between magnetite bands in the Dales Gorge BIF. The stilpnomelane layers are intermixed with lenses of Fe-talc, and contain magnetite, apatite and ankerite inclusions (Fig. 4h, i). These stilpnomelane and Fe-talc layers are not associated with, nor contain OM (Fig. 4h, i). Laminated stilpnomelane layers in the Dales Gorge BIF are frequently overgrown by rhombohedral siderite (Fig. 4h, i), which contain microscopic inclusions of OM (Fig. 4i), although no OM occurs within the stilpnomelane. In BIF layers from the Dales Gorge section, apatite forms layers of 50–500 µm thickness (Fig. 5a), or individual clusters overlying magnetite layers (Fig. 5g). In transmitted light, the apatite is often dark grey due to inclusions of OM, magnetite and haematite (Fig. 5a, g). The apatite layers are generally associated with layers of magnetite and minnesotaite or stilpnomelane (Supplementary Table 2) (Fig. 5a). The apatite layers include laths of minnesotaite and microscopic haematite, siderite, and pyrite (Fig. 5a). Pyrite occurs within apatite and surrounding the apatite layers (Fig. 5a). Carbonate in the Dales Gorge BIF occurs as irregular masses (Fig. 5d, e) or rhombohedra. Within irregular ankerite masses, pockets of OM, haematite and magnetite co-occur (Fig. 5d–f). Also, in haematite layers in the Dales gorge BIF microscopic particles of OM are found bound to microscopic particles of haematite (Fig. 6). Bulk rock δ^13^C_Carb_ and δ^13^C_Corg_ for the Dales Gorge BIF samples are −8.0 to −10.5‰ and −23.6 to −24.8‰, respectively (Supplementary Table 3).

## Discussion

**Fig. 7:**
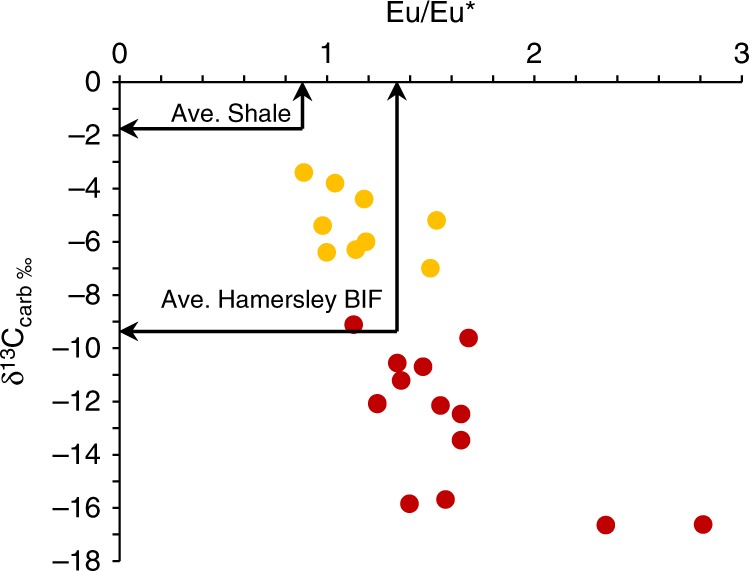
Correlation between hydrothermal input and δ^13^C_carb_ in BIF. Note increasing hydrothermal input corresponds to increasing depletions in δ^13^C_carb_. Yellow points are from Teixeira et al.^18^, and red points from Smith et al.^17^. BIF from Teixeira et al.^18^ are carbonate-magnetite-quartz BIF; BIF from Smith et al.^17^ are laminated magnetite-siderite chert-banded iron formation or laminated ferhythmite. Average Paleoproterozoic shale and BIF values after Alibert and McCollom^19^

The ^13^C-depletions of carbonate in BIF, has been attributed to oxidation of isotopically light carbon in OM^3^. Alternatively the low δ^13^C values of carbonate in BIF may be a result of carbon exchange between the mantle and seawater^14^. Rare earth element profiles of marine Archean sediments often carry pronounced Eu anomalies compared to modern seawater profiles, which is attributed to hydrothermal influence on marine waters^15^. Therefore volcanic outgassing in the Archean may have produced a ^13^C-depleted inorganic carbon pool through mixing with marine and mantle carbon reservoirs^14^, leading to the precipitation of ^13^C-depleted carbonate in BIF (Supplementary Fig. 1). Also, it has been observed in modern hydrothermal basins, brine pools around hydrothermal vents may carry bicarbonate with δ^13^C values of −7‰, and that Fe-Mn carbonates precipitate from mixtures of this brine water and overlying seawater, producing positive trends in δ^13^C and δ^18^O^16^, which has been observed in oxide/ silicate BIF^3^. However, some BIF were deposited distally to sites of hydrothermal activity, therefore dilution of carbon isotope signatures during transport would reduce the influence of hydrothermal fluids on BIF formation. This might be evidenced by shallow-water BIF having more seawater-like carbon isotope signatures compared to their deeper water counterparts^14^. If hydrothermal fluids influenced δ^13^C values in BIF, we might expect to find correlations between hydrothermal input and δ^13^C_carb_ in BIF. Compiled data for coupled Eu/Eu* and δ^13^C_carb_ measurements in BIF from the Witwatersrand^17^ and Minas groups^18^ (Fig. 7) exhibit decreasing δ^13^C_carb_ values with increasing hydrothermal input (Eu/ Eu*), suggesting that the low δ^13^C_carb_ values in BIF may be partially accounted for by mixing of mantle and seawater water carbon reservoirs^14^. Expectedly, BIF carry higher Eu/ Eu* values and more negative δ^13^C_carb_ values than associated shales^19^. The carbon isotopic composition of hydrothermal vent fluids is widely ranging and can be between −2 and −40‰, with the majority falling between −4 and −9‰^20^. Such a range could account for the variable δ^13^C composition of carbonate in BIF. This scenario may also explain the low δ^18^O values of carbonate in BIF, which can be explained as a result of high rates of fluid exchange between volcanic crust and seawater^21,22^, that would lower the δ^18^O values for seawater in BIF depositional environments^14^. If the carbon isotopic composition of the surrounding seawater approached mantle values, less OM is required to be oxidised in order to explain the low δ^13^C of carbonate in BIF (Supplementary fig. 1). Additionally, the metamorphic grade of the BIF studied here, are all at or above the greenschist facies, therefore δ^13^C_carb_ could have been lowered by 1–3‰ due to metamorphic alteration^23^.

Nevertheless, there are multiple lines of evidence for iron and carbon cycling in some BIF (Fig. 5). The isotopic composition of carbon in carbonate from some BIF is lower than −10‰^3,17,24^ (Supplementary Table 3), suggesting average mantle carbon values might not be solely accountable for the isotopic depletions (Supplementary Fig. 1). For instance, in the Dales Gorge BIF, δ^13^C_carb_ values of −8.0 to −10.5‰ for carbonate (Supplementary Table 3), which hosts mixtures of microscopic particles of OM, haematite,magnetite and apatite (Fig. 5e, f, g), is in agreement with OM oxidation coupled to ferric iron reduction. Therefore, the presence of apatite layers containing inclusions of OM in the Dales Gorge BIF (Fig. 5b), and OM in carbonate (Fig. 5f), is compelling evidence for oxidation of OM in some BIF layers^13^.

Additionally, depletions in δ^56^Fe in BIF have been proposed to reflect dissimilatory iron reduction; however δ^56^Fe enrichments are more prevalent than depletions^5^. Also, shales carry more pronounced depletions in δ^56^Fe than BIF, suggesting shallower marine sediments may have had a more active iron cycle, and therefore some low δ^56^Fe values in BIF may be sourced from a dissimilatory iron reduction shuttle^25^.

Currently Fe-clays in BIF are thought to have formed either diagenetically within iron-silica gels^26,27^, or via precipitation from seawater by inorganic chemical reaction of dissolved iron and silica^6,28,29^. In the latter case, minerals such as greenalite, or stilpnomelane (if Na, K, and Al are present^4^), are then expected to flocculate like iron-oxide particles and deposit on the seafloor. Both greenalite and stilpnomelane are phyllosilicate minerals and part of the kaolinite-serpentine and smectite groups, respectively. Greenalite undergoes structural changes to form minnesotaite - part of the pyrophyllite-talc group − at low grade metamorphism^4,6^. At high grade metamorphism, minnesotaite and stilpnomelane recrystallize into double-chain inosilicate minerals, such as grunerite or actinolite^4^ (Supplementary Table 6).

The direct association of OM with stilpnomelane in ferruginous chert bands in the Dales Gorge Formation (Fig. 3a) suggests this smectite-group clay binds to and preserves OM, like many other clays. Similarly, it can be inferred that the sheet-silicate greenalite would also act in a similar manner, trapping and binding particulate OM in the water column, which is corroborated by observations showing that serpentine minerals adsorb OM in soils^30^. Therefore greenalite forming in BIF depositional environments should bind to organic material in the water column, and protect it from degradation^9,31^. Organo-silicate complexes persist through metamorphism as evidenced by the preservation of consistently graphitised OM in smectite, illite and mica (Fig. 2)^32^. OM bound to greenalite or stilpnomelane is therefore expected to persist through recrystallisation during metamorphism, as greenalite and stilpnomelane recrystallise to minnesotaite and grunerite at progressively higher grades metamorphism. The association of graphite within grunerite in the Pääkkö sulphidic iron-formation (Fig. 3b) demonstrates that metamorphic recrystallisation of clays still preserves OM within the clay. The association of grunerite and graphite has also been documented in Neoarchean turbidite deposits, interbedded with BIF in the Slave Craton^33^, supporting its ability to preserve OM through metamorphism. However, multiple Raman scans of various sheet-silicates in BIF, including minnesotaite, stilpnomelane, Fe-talc, grunerite and actinolite, shows little to no OM within or associated with these silicates. The lack of OM or graphite in Fe-silicates within BIF (Fig. 4) is unusual, given phyllosilicates in other marine sediments preserve OM through diagenesis and metamorphism (Fig. 2). The low levels of OM in BIF is often attributed to microbial or diagenetic oxidation of OM with ferric iron, yet the abiotic, diagenetic oxidation of OM requires direct contact with the oxidant, and OM bonded within silicates would prevent this reaction from proceeding. Additionally, clays have been shown to inhibit heterotrophic bacteria from decomposing OM, by restricting enzymes from entering the pore spaces in which OM is held^12,31^.

The relatively common association of OM with carbonate and apatite, but not Fe-clay layers in BIF is puzzling, as the clays should have scavenged and preserved OM from the water column, as shown in Figs. 2–3. Two possible scenarios to explain this observation would be the deposition of OM in BIF sediments was decoupled from Fe-clay deposition, or Fe-clays in BIF formed during late-diagenesis. It has been suggested the deposition of iron and quartz layers in BIF were decoupled^34^. Therefore, it could be possible that OM was deposited slowly following deposition of Fe-clays and Fe-oxides in a water column with low OM sedimentation rates, as a result OM could avoid capture by Fe-clays.

Alternatively, if Fe-clays formed during late-diagenesis, then they would not capture and preserve OM from the water column. Additionally, it was suggested that certain fossilised microbes from the Gunflint Formation produced Fe-silicates in vivo^35^, however in this instance the Fe-silicates occur within and in contact with organic-walled microfossils. This is similar to other clay-bearing microfossils from the Torridonian Group^36^, which can enhance the preservation of OM. Also, REE patterns of greenalite in the Hamersley BIF were interpreted to reflect a diagenetic origin for the Fe-silicate^37^. If Fe-silicates formed diagenetically, it could occur through the reduction of ferric-oxides with OM oxidation. This could liberate ferrous iron, which may subsequently react with silica or ferric iron in a seafloor gel to form iron-silicates especially under alkaline conditions^28^. Therefore the Fe-silicate clays would post-date OM and not necessarily capture the OM. Alternatively, ferrous iron in pore water could be sourced from the overlying ocean. In contradiction, experiments mimicking BIF diagenetic chemistry found mixing OM, ferric oxides and silica did not produce ferrous silicates, but only siderite and magnetite^38^. Nevertheless, if Fe-silicates were forming during iron-reduction, the newly formed diagenetic clays would adsorb to, or encapsulate the remaining OM and/ or microbes, as is seen around microbes^39,40^, preventing further OM oxidation^12^, and therefore OM should be preserved within Fe-silicates in BIF, which is evidently not common in our samples (Figs. 4 and 5).

Experimental observations show amorphous Fe-silicates readily form within Precambrian seawater solutions^28,29,41^, therefore a sole diagenetic origin for Fe-silicates seems unlikely. Furthermore, REE patterns for stilpnomelane and minnesotaite in BIF from the Transvaal group were interpreted to reflect a primary seawater origin for these Fe-silicates^42^. Additionally, petrographic observations show nanoscopic crystals of greenalite preserve primary sedimentary laminations that predate diagenetic shrinkage structures, which is consistent with low temperature precipitates^6^, and not diagenetic products. These nanoscopic silicates should have exceptional ability to sequester OM, as it has been observed that finer grained silicates are the most potent preservers of OM in sediments, due to their higher surface to volume area ratios^8^. Therefore, the relative lack of OM preserved in Fe-silicates within BIF, suggests Fe-clay deposition was decoupled from OM deposition in BIF. The subsequent decay of any OM depositing in the BIF sediments led to the formation of apatite layers and contributed ^13^C-depleted carbonate hosting the residual OM. According to isotope mass balance calculations for the Dales Gorge BIF, 2.3–4.2 wt% of OM needs to be oxidised in order to account for the observed carbon isotopic compositions of the BIF (Supplementary Fig. 1). These large quantities of OM are of a similar magnitude to TOC in pelite associated with BIF^27^. However, in the pelite samples of this study, it is estimated around 80–95% of the observable OM occurs with or within phyllosilicates, yet in BIF samples no OM was found with phyllosilicates. Given the protective capabilities of clays^31,32,40,43^, it would appear unfeasible to expect no OM to remain in clays within BIF after the estimated original 2–4 wt% of TOC is removed. In addition to the lack of OM in Fe-clays within BIF, previous studies have shown that trace elements such as Ba, which correlates with high organic productivity^44^ − are depleted in BIF compared to OM-bearing sediments associated with BIF^14^. Similarly, low Mo abundance and low δ^98^Mo values in the Hamerlsey BIF compared to shale, were used to infer adsorption of isotopically light Mo to Fe-oxyhydroxides was more important than Mo adsorption to OM, implying OM deposition was low^45^.

The initial mineralogy of BIF and metapelite is different, as are their respective clays. The clays in pelite are Al-bearing, while those in BIF are predominately Fe and Mg-bearing (Supplementary Table 2), however the composition of clays in BIF and metapelite (Supplementary Table 6) has been shown to make little difference in their ability to bind OM, as stilpnomleane preserves OM just like muscovite (Figs. 2–3). Furthermore, while clays in BIF could have been water column precipitates, and clays in pelite detrital, this difference in origin still permits both clay-types being suspended in the water column, and therefore open to capture and preserve OM. This principle is also applicable to iron formations, as granular iron formations contain similar clay types (minnesotaite, greenalite, stilpnomelane^46,47^) to banded iron formations, and have been found to exhibit higher organic carbon contents in clay-rich varieties^47^, and organic matter is found in clays within these clay-rich granular iron formations, as is found in pelites. Therefore, the comparison of OM-clay associations in BIF and pelite is appropriate. However, granular iron formations formed in shallower water settings compared to banded iron formations, therefore depositional mechanics would have been different. Nevertheless, it is unlikely the depth of deposition greatly affects the ability of Fe-clays to bind with OM. Moreover, granular and banded iron formations are both predominantly chemically-precipitated sediments^4,48^. Therefore granular iron formations show clays in chemically-precipitated sediments exhibit the same ability for OM preservation as those clays in clastic sediments.

Banded iron formations generally have less clay compared to metapelite; we estimate this using whole rock MgO, given Mg forms roughly the same percentage of clay minerals in both BIF and metapelite (Supplementary Table 2). However, in BIF Mg may also constitute up to 10% of carbonate minerals, considering carbonate average around 5 wt% in BIF, Mg in carbonate would shift total Mg by 0.5 wt% of BIF. On the other hand, many Fe-clays in BIF may have no Mg (e.g. Fe-talc in Fig. 5) and therefore bulk rock MgO may underestimate total clay content. The ratio of MgO in Pelite/ BIF varies from 1.8 to 9.8 with an average of 3.2 (Supplementary Table 5 and Supplementary Fig. 2), which gives BIF about 31% of the clay content of metapelite, therefore BIF should also have 31% less clays containing OM. From petrographic estimates the pelite samples in this study contained 50–80% clay minerals, and BIF contained 5–80% with the majority having 20–30% Fe-clay content (Fig. 1), which equates to BIF having 25–40% of the clay content of pelite, which is similar to the estimate using whole rock MgO. The McRae shale which forms part of the Hamersley iron formation has an average of 2.5 wt% TOC^45^, so if most of the OM is bound in clays − which is observed here (Fig. 2) − then from extrapolation, BIF should preserve 31% of this TOC, because BIF have 31% the clay content of pelite giving 0.78 wt% TOC, instead the average TOC of BIF is 0.03 wt% (Supplementary Table 3) or 17 times lower. Other factors will control OM preservation, not only clay content, however this calculation emphasises that the clay content of BIF is sufficient to preserve more OM than is observed.

BIF have more ferric iron than metapelite which could have oxidised more OM, therefore the original abundance of ferric iron will affect the preservation of OM and TOC in BIF and metapelite. The average iron oxidation state of BIF is estimated as Fe^+2.4^ ^49^, which equates to 60% ferrous and 40% ferric iron, whereas the average oxidation state of iron in pelite before the Great Oxidation Event is 80% ferrous and 20% ferric iron^50^. Therefore, more ferric iron was available for iron reduction in BIF. However, the published ferric iron content in BIF from the Transvaal Supergroup, S. Africa, show a positive correlation with OM (Supplementary Fig. 3A), which is the opposite trend expected for ferric iron reduction coupled to OM oxidation. In modern marine sediments, iron is believed to be a sink and preserver of OM in the sediments, and OM bound to iron possibly accounts for up to 21% of the total OM in modern sediments^51^. The observed co-occurrence of microscopic particles of OM and haematite in the Dales Gorge BIF (Fig. 6) supports this interpretation.

In BIF and metapelite from the Transvaal Supergroup, published TOC values are positively correlated with Al (*r*^2^ = 0.33) (Supplementary Fig. 3B-D), supporting a similar source of OM in BIF and metapelite. Since Al is typically attributed to being an element in detrital minerals sourced from weathered continental rocks, OM could also have been sourced from detritally enriched, shallow-marine environments. Continental weathering input to the Hamersley and Pongola BIF is supported by previous work, which has shown silica-rich layers contain Ge/Si ratios similar to Ge/Si ratios in modern continental run-off^34,52,53^. This is exemplified in the Pongola BIF where increasing Al content correlates with a shift in Ge/Si ratios toward more continental-like ratios (Supplementary Fig. 4E). Therefore, the correlation of OM with Al in BIF and metapelite, suggests a significant proportion of OM in these sediments may have been exported with detritus from biologically productive shallow-marine environments. However, there is a stronger correlation (*r*^2^ = 0.55) between TOC and ferric iron, than TOC and Al in the Transvaal BIF (Supplementary Fig. 3A, D), which suggests OM was sourced from regions of ferric iron production, or was selectively preserved by ferric-oxyhydroxides^51^.

**Fig. 8:**
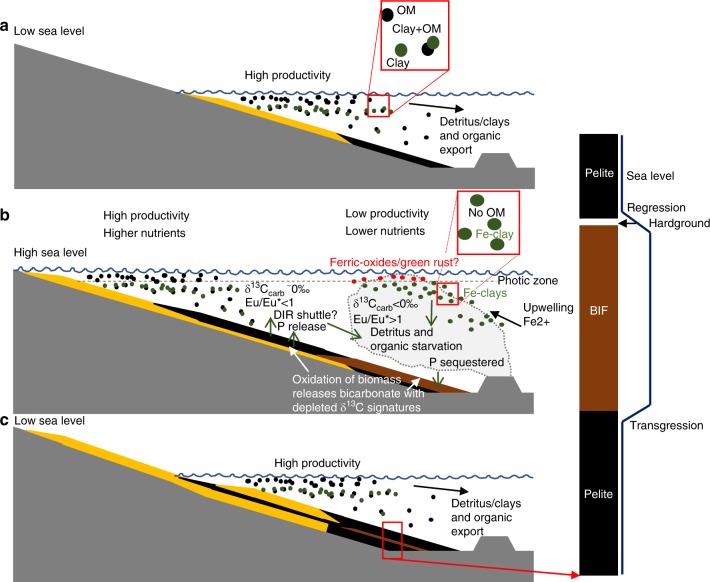
Model for the deposition of BIF. **a** Low sea level allows extensive export of organic matter and detritus from shallow marine environments to far shelf, slope and abyssal environments. Organic matter and clays complex in the water column and are deposited in the sediments together which protects organic matter from oxidation. **b** Higher sea level reduces the export of organic matter from the shelf and starves the deeper basin of organic matter and detritus. Exhalation of hydrothermal fluids leads to precipitation of various iron-rich minerals, Fe-clays precipitate during mixing of hydrothermal fluids and seawater in a water column devoid of organic matter, therefore decoupling their deposition together in BIF precipitates. Small quantities of organic matter exported from shallow marine environments may be deposited and oxidised in conjunction with ferric iron reduction. **c** Following a hiatus in hydrothermal exhalations, hardgrounds form on the detritus starved seafloor. Sea level falls, returning plentiful organic matter and detritus export to deeper parts of the basin. Yellow layers represent shallow marine sediments, black layers represent shale, and brown represents BIF

Numerous studies have proposed iron formations were deposited during marine transgressions^54–56^. This would have led to BIF being deposited in basins starved of detritus (Fig. 8), which is supported by chert hardgrounds found atop of the Brockman Formation^57^. If OM was sourced from shallow marine environments as indicated by its correlations with Al (Supplementary Fig. 3B–D), then OM too would have been in minimal supply at BIF depositional sites^55,58^. The OM-starved water column above BIF sediments would be expected to have Fe-clays and ferric-oxyhydroxides deposit from a water column with minimal complexation with OM (Fig. 8), as seen in the samples here (Figs. 4–5). Small amounts of OM may have been exported from shallow marine environments and deposited in BIF to give the observed correlation with Al (Supplementary Fig. 3; Fig. 8). The small amounts of OM deposited in BIF were then quickly oxidised by ferric iron to form apatite and carbonate (Fig. 5).

The composition and mineralogy of BIF is heterogenous and dependant on their respective depositional sites. Generally, shallower water BIF are carbonate-rich, whereas deeper water BIF are iron-oxide-rich^17,55,58^, both of which can have variable amounts of Fe-silicates. These shallow water BIF contain about an order of magnitude more OM and Al than deeper water BIF^55,58^, suggesting they received more organic and detrital input from coastal regions (Fig. 8). As a result iron-reduction would be more prevalent in shallow water/carbonate-facies BIF, than in deep water/ oxide-facies BIF. Thus, our model predicts a more active iron cycle in nearshore BIF. Whereas deeper water BIF would have a less active iron cycle, therefore retaining their initial ferric components and adsorbed nutrients. This results in preferential nutrient regeneration in shallow water BIF, acting to maintain productivity in coastal waters and restrict it in the open-ocean.

**Fig. 9:**
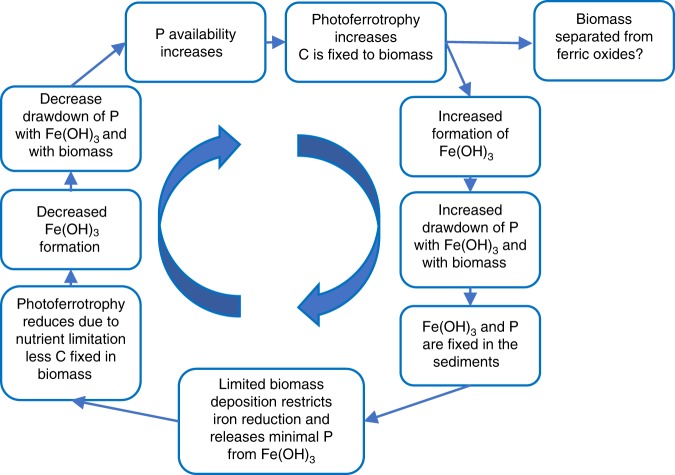
Negative feedback model for BIF biogeochemical iron cycle. Minimal deposition of biomass in banded iron formations prevents phosphorus release from ferric oxides in the sediments, creating a phosphate limited growth cycle for photoferrotrophs

The consequences of low OM deposition in BIF, especially deep-water BIF, would produce a negative feedback cycle on early ecosystems (Fig. 9). If photoferrotrophy was active, OM could have been bound with ferric-oxyhydroxides and deposited in BIF sediments, however iron-oxidising bacteria often separate themselves from iron particles to avoid encrustation, thus resulting in OM-poor metalliferous sediments^59^ (Fig. 8). Therefore ferric oxyhydroxides produced by photoferrotrophs or by abiotic processes such as photo-oxidation^60^ or reaction with dissolved O_2_^61^ would adsorb phosphorus and other nutrients removing them from the photic zone and depositing them in the sediments. The inferred low totals of OM deposited in BIF would have been rapidly oxidised to produce apatite and δ^13^C-depleted carbonate (Fig. 5), but insufficient to support widespread reduction of ferric iron, and therefore prevent the return of phosphorus to the water column, leading to upwelled waters being poorer in nutrients. This in turn would lead to a less productive water column, therefore decreasing ferric iron production and BIF formation rate, and decreasing phosphorus drawdown, thus allowing phosphorus and nutrient levels to build again (Fig. 9). If photoferrotrophs were the major control on BIF formation, this would produce a self-limiting biogeochemical cycle which creates cycling periods of low and high rates of biological activity and BIF deposition, but never stopping BIF formation. The self-limiting biological formation of ferric iron would decrease the overall oceanic drawdown of phosphorus by iron oxides during the Archean^62^. Alternatively, if ferric iron was produced abiotically, then nutrient scavenging would simply restrict biological productivity during periods of elevated ‘rust’ production. In summary, associations between minerals and OM in BIF provide evidence for iron-reduction coupled to OM oxidation, however the specific association of clays and OM in BIF and pelite, suggests that initial OM levels in BIF were too low to support widespread iron-reduction, leading to a negative feedback biogeochemical cycle (Fig. 9).

The presence of OM-bearing apatite and carbonate in BIF^13^, point to oxidation of biomass by ferric iron, which would have contributed to the low δ^13^C values in carbonate in BIF and released any adsorbed nutrients^62^. However, the relative scarcity of OM in Fe-clays within BIF, and a possible hydrothermal source for negative δ^13^C values in BIF, collectively suggest the deposition of OM in BIF could have been much lower than previously thought. This may be due to one, or a combination of possibilities, including BIF forming in basins, far from regions of primary productivity, and/or a nutrient-limited open ocean biosphere^63^, and/or the separation of OM and iron by photoferrotrophs. It is estimated BIF initially had 1–10 wt% TOC, assuming OM oxidation was the sole source of ^13^C-depleted carbonate in BIF^27^. However, the lack of OM preserved in Fe-clays within BIF suggests the initial amount of OM may have been much lower, and more similar to modern open-ocean sediments, which contain only 0.1–0.6 wt% TOC^64^. Therefore the low δ^13^C_carb_ values in BIF is more likely the result from mixing of ^13^C-depleted carbon from both oxidised biomass and the mantle.

If the initial availability of OM for iron reduction was limited as proposed here, ferric iron reduction would be limited, and therefore phosphorus and other nutrients would remain bound to ferric iron in BIF. This would limit the release of nutrients to the overlying water column and leading to a negative feedback loop. Such a scenario could decrease the importance of biologically-formed ferric iron in BIF, which would reduce biologically-mediated phosphorus drawdown in the Precambrian ocean and stifle the iron cycle, especially in deep basins.

## Methods

### Analytical methods

Optical and micro-Raman microscopy: An Olympus BX51 microscope with 5× , 10× , 20× , 50× and 100× objectives was used to conduct transmitted and reflected light optical microscopy on thin sections, 30 µm in thickness and polished to 0.25 µm with Al2O3 powder in DI water; no oil immersion was used. Micro-Raman imaging was conducted at the London Centre for Nanotechnology in University College London with a WITec α300 Confocal Raman Imaging system. A green 532 nm laser was used with a power <6 mW and was focused at 500× for large area scans (> 400 × 400 µm) and 1000× for smaller area scans, achieving spatial resolutions between 2000 and 360 nm. A 50 µm diameter optic fibre cable was used to collect Raman spectra at confocal depths of at least 1 µm below the surface of thin sections. Each pixel collected a Raman spectrum with a typical dwell time of 0.4–0.6 s. All Raman spectra were corrected for cosmic rays using the cosmic ray reduction function in the WITec Project Four Plus software. All Raman spectra herein were selected from pixels with nearly identical spectra and the averaged spectra were corrected with a background subtraction polynomial fit, typically of the order of 3–7. Raman hyperspectral images of mineral associations were generated by mapping main peak intensities for mineral-specific peaks using the WITec Project Four Plus data processing software. For estimation of crystallisation temperatures of graphic carbon, peaks were modelled using a Lorentzian function to extract peak areas, FWHM and wavenumber.

### Electron microscopy

Scanning electron microscopy (SEM) and energy dispersive spectroscopic (EDS) analyses were performed using a JEOL JSM-6480L SEM in the Department of Earth Sciences at University College London. Operating conditions for SEM imaging and EDS analysis involved a 15 kV accelerating voltage for an electron beam current of 1 nA, with a working distance of around 10 mm. Polished thin sections were cleaned with isopropyl alcohol and dried with dry N2, before deposition of a few nanometres of Au (1 or 2 min coating under a current of about 1.8 mA in Ar) prior to SEM analyses. Elemental compositions were calculated by software using ZAF correction and normalized to 100%. On the basis of repeated measurements on Specpure metal and silicate standards, we estimate the error on the reported values to be ~1%.

### Isotope Ratio Mass Spectrometry

Analyses of bulk rock powders for isotopic compositions of OM were conducted in the Bloomsbury Environmental Isotope Facility at UCL with a Thermo-Finnigan Flash 1112 EA connected to a Thermo Delta V Isotope Ratio Mass Spectrometer via a Conflo IV gas distribution system. Bulk rock powders were prepared using clean techniques that involved crushing in a steel mortar and pestle, cleaned with muffled quartz chips and DI water between samples. Acid-insoluble OM was obtained by accurately weighing between 5 and 80 mg of bulk powder in Ag boats (pre-muffled at 600 °C for 2 h) and subsequently decarbonated with 10% ultrapure HCl, and finally dried in air in a laminar air flow hood. The dried residue was then loaded into a second muffled silver capsule, placed in an autosampler, and dropped into the furnace of the Thermo-Finnigan Flash 1112 EA. An ultrapure He carrier gas was used for the procedure in continuous flow mode. Bursts of reference gas were injected prior to each run to calibrate against. A suite of standard materials were analysed within each run that span a range of δ^13^C values from −26‰ to −6‰. Each standard was analysed multiple times through the run to ensure a reproducibility better than 0.2‰ for all runs. Empty muffled silver capsules were processed as procedural blanks with and without HCl added, to ensure that no C was detected.

Analyses of bulk rock powders for carbonate were conducted in the Cardiff School of Earth Sciences with a Thermo Finnigan Delta V Advantage mass spectrometer connected to a Gas Bench II. Crushed sample powders in vials were acidified with >99% H_3_PO_4_ by manually injecting the acid using a syringe. All samples and standards were left to react for 4 days at 60 °C before analysis. The evolved CO_2_ from the acid dissolution of the carbonates in the powder were then fed into a mass spectrometer. The reproducibility for δ13CCarb and δ18 OSMOW was better than ± 0.1‰ (1σ), based on multiple measurements of an in-house standard of Carrara marble (calcite). Measured ^18^O/^16^O ratios were corrected for the respective carbonate mineralogy, inferred from EDS and Raman analyses, using acid fractionation – temperature equations for siderite (Fernandez et al., 2016) and ankerite (Rosenbaum and Sheppard, 1986).

### Bulk rock geochemical analysis

The bulk major and trace element analyses were collected by ICP-AES and ICP-MS, respectively, in the Laboratory for Environmental Geochemistry at the University of Colorado at Boulder according to standard procedures. Briefly, about 100 mg of powders were digested in a heated solution of nitric and hydrofluoric acid before their introduction into the instruments. A number of measurements on standards were performed for different trace element concentrations, using four orders of magnitude for dilutions and the results were corrected for blanks. All trace elements are reported in ppm with an error of about 10%, estimated from duplicate analyses of powdered aliquots from the same sample.

## Supplementary information

Supplementary Info

Peer review

## Data Availability

All data generated or analysed during this study are included with this article (and its supplementary information files).
