# Supplementary material for: Minimal biomass deposition in banded iron formations inferred from organic matter and clay relationships
Source: Nat Commun. 2019 Nov 4;10:5022. doi: 10.1038/s41467-019-12975-z (PMC6828686; doi:10.1038/s41467-019-12975-z)
Supplement: Supplementary file 1 — Supplementary Info [file 41467_2019_12975_MOESM1_ESM.pdf]

Supplementary Information for

Minimal biomass deposition in banded iron formations inferred from organic matter and clay relationships

by Dodd et al.

Supplementary Notes 1

Samples and geological descriptions

BIF and metapelite from the Eoarchean Isua Supracrustal Belt, SW Greenland

The Isua supracrustal belt outcrops in south-west Greenland, and is chiefly comprised of amphibolite, felsic gneiss, and interbedded schist and BIF [3,4]. Zircons obtained from schists accepted to represent felsic volcanics gave U-Pb ages of 3,703 $\pm$ 3 Myr [5]. Rare, zircons isolated from chemical sediments in the belt have been interpreted as detrital grains sourced from felsic volcanics, and give U-Pb ages from 3,690 to 3,940 $\pm$ 10 Myr [5], whereby setting a maximum age of 3,690 $\pm$ 10 Myr for the sediments. Assemblages including garnet+staurolite+biotite and kyanite+biotite were used to determine metamorphic temperatures and pressures of 550° C and 5 Kbar; in addition, rare relict inclusions of chloritoid in garnet porphyroblasts define a maximum temperature of prograde metamorphism at 600° C [6]. The metapelite (Fig. 1A) is predominately comprised of chlorite, garnet and muscovite, while the BIF (Fig. 1B) is mainly composed of quartz, magnetite and actinolite (Supplementary table 2).

BIF and metapelite from the Neoarchean Anshan Group, Liaoning

The Anshan area is situated in the northeastern part of the North China Craton. The supracrustals in the area comprise metapelites, such as quartz-mica schists, and also BIF and minor metavolcanic units. The BIF units form repeated cycles of BIF and metapelites, and are interpreted to have been deposited in an arc-basin [7]. The belt was variably metamorphosed at the upper greenschist to upper amphibolite facies [7]. Zircon U-Pb dating shows that magmatic zircons from metavolcanic rocks in the Anshan area were recrystallized at 2,551 ± 10 Myr, representing the minimum age of the Anshan BIF, while metamorphic zircons were formed at 2,469±23 Myr, reflecting the age of a later metamorphic event [8]. The youngest group of detrital zircons from the metapelite samples, constrain the maximum depositional age of the metapelites, which are interleaved with the BIF, to around 2,540 Myr [7]. The depositional age of the BIF and metapelite therefore can be constrained between 2,540 and 2,510 Myr [7]. This is supported by further Secondary Ion Mass Spectrometry U-Pb zircon dating on metavolcanic and metasedimentary rocks of the Anshan Group in the Anshan-Benxi area [9]. Samples of metapelite (Fig. 1C) and BIF (Fig. 1D-E) were taken from a drill core taken several kilometres southeast of Qidashan, Liaoning province. The metapelite is comprised of muscovite and chlorite layers, which alternate with quartz layers, sulphide forms small lenses with chlorite throughout (Supplementary table 2). The BIFs are composed mainly of quartz, magnetite, grunerite and ankerite, one BIF sample contains little quartz and has a matrix of grunerite (Fig. 1E).

BIF from the Neoarchean Temagami Supracrustal Belt, Ontario

The Temagami BIF (Fig. 1F) is an oxide-facies BIF, found in the Temagami Supracrustal Belt in Ontario, Canada. The sample contains millimetre thick layers of magnetite and quartz, apatite layers up to a millimetre in thickness, minnesotaite and ankerite (Supplementary table 2). The sample was collected from tailings inside the Sherman mine in Ontario. The belt comprises one metasedimentary unit and four metavolcanic units. The BIF occurs at the top of the uppermost metavolcanic unit and is associated with turbiditic greywacke and shale, all of which have been metamorphosed to the lower greenschist facies [10]. The Iceland Lake pluton and a nearby contemporaneous rhyolite flow stratigraphically below the BIF, yield U-Pb ages on zircons of 2,736±2 Myr and 2,736±3 Myr respectively [11]; the youngest plutonic activity is the emplacement of a late rhyolite porphyry dike at 2,687±2Myr [11]. This places the age of the BIF, between 2,736±3 to 2,687±2Myr.

BIF from the early Palaeoproterozoic Dales GorgeIron Formation, Western Australia

Samples of the Dales GorgeBIF (Fig. 1G-J) were taken from drill core DGH-1, which was drilled through the Dales Gorge Member; the lowermost portions of the Dales GorgeIron Formation in the Hamersley Supergroup, north Western Australia. The BIF samples vary widely in the relative abundances of minerals, all contain magnetite apart from one sample (Fig. 1J), all contain quartz, as

well as siderite, apatite forms bands up to hundreds of microns in thickness along magnetite bands (Fig. 1H-I). OM forms visible laminae in one sample (Fig. 1J), where it almost exclusively occurs with stilpnomelane (Supplementary Table 2). On the basis of U–Pb dating on zircons extracted from tuffaceous bands, a depositional age between 2,494 and 2,464Myr has been determined for the Dales Gorge member [12]. Oxygen isotopes of co-existing chert and magnetite, along with observable mineralogies, suggest the BIF did not exceed lower greenschist facies [13,14]. The BIF outcrops with interbedded black shales forming a repeating sequence of 16 individual BIF and black shale layers [15].

Metapelite from the Palaeoproterozoic Pecors Fm, Ontario

The Pecors formation is an argillite composed predominately of quartz, mica and sulphides (Fig. 1K; Supplementary table 2), and forms part of the Hough Lake Group of the lower Huronian Supergroup, which outcrops along the northern shore of Lake Huron in Ontario, Canada. The supergroup is an early Palaeoproterozoic volcanosedimentary succession more than 10 km in thickness. The Huronian Supergroup is constrained in age by a basal rhyolite formation with a zircon U–Pb age of 2,452 $\pm$ 6 Myr [16], and by a baddeleyite U–Pb age of 2,219.4 $\pm$ 4 Myr from the Nipissing diabase, which intrudes the supergroup [17]. The Huronian Supergroup is proposed to represent the product of a Wilson cycle with early sedimentation in an active rift basin, followed by subsequent deposition in a passive margin setting [18]. The Huronian group is punctuated by three glacial and interglacial cycles represented by diamictites. The Pecors Fm. directly overlies the first glacial cycle, and is interpreted to have been deposited in a distal deltaic environment that precedes the formation of the overlying Mississagi delta [18].

Iron Formation from the Palaeoproterozoic Pääkkö Fm, eastern Finland

The Pääkkö iron formation is included in the Lower Kaleva successions of the Väyrylä Group in eastern Finland within the Kainuu Belt [19]. It is dominantly comprised of amphibole, quartz and magnetite, with siderite and sulphide rich horizons (Fig. 1L; Supplementary table 2). The iron formation occurs as ca. 0.5m thick beds within black schists which overlie mica schists [20]. The sequence has been metamorphosed to low amphibolite facies [21]. Based on U-Pb isotopic data from detrital zircons, the Lower Kaleva sedimentation is constrained roughly to 1,920-2,000 Myr [19,22].

Metapelite from the Palaeoproterozoic Tuomivaara Fm, eastern Finland

The Tuomivaara metapelite forms part of the Tuomivaara Formation of the Sotkamo Group, within the Lower Kaleva successions of the Kainuu belt of central Finland [19]. The Tuomivaara Formation consists of amphibolite with tholeiitic affinities and interbedded chemical and clastic sedimentary rocks. Chemical sediments include iron-formation and phosphorus beds, while the clastic sediments include garnetiferous and sulphidic pelite (Fig. 1M; Supplementary table 2), and sandstone beds, which exhibit graded bedding. The clastic sediments form thin beds from 10 to 100 cm in thickness between several cycles of sulphide, silicate and oxide facies iron-formation. The formation is suggested to have been deposited under low-energy conditions, with interspersed turbiditic flows and volcanic activity in an offshore rift basin [23]. The Tuomivaara area Kainuu schist Belt was deformed and regionally metamorphosed at the middle amphibolite facies during the Svecofennian Orogeny 1,885 – 1,900 Myr ago [21]. Constrained by detrital zircon U-Pb-data, the depositional age of the Lower Kalevian successions is about 1,920-2,000 Myr [19,22].

Supplementary Table 1. Description of samples used in this work

Formation name and sample numbers	Location	Age (Myr)	Metamorphic facies	Rock type	Mineralogy	Figures
Isua (GRDP0402; GRDP0404)	SW Greenland GRDP0402: N65°08’83.2” W50°10’15.9” GRDP0404: N65°10’17.4” W49°48’52.9”	Ca. 3, 700	Amphibolite	BIF	Qtz+mag+act+py+apa	5E
				Pelite	Chm+qtz+grt+mus+ann+ilm+apa+cal	2A-B
Temagami (TE0704)	Sherman mine, Ontario, Canada	Ca. 2, 700	Greenschist	BIF	Qtz+mag+mns+ank+apa+gra	5F-G
Anshan (ANS0904; ANS0917; ANS1507)	Drill core taken several kilometres south east of Qidashan, Liaoning province	Ca. 2,550	Amphibolite	BIF	Qtz+mag+gru+py+apa+cal	5A-D
				Pelite	Qtz+mus+chm+alb+gra+py+rt	2D-E
Brockman (DGM-1-127-5; DGM-1-169'6; DGM-1-189'6; DGM-1-249; DGM-1-273-10)	DGH-1 drill core	Ca. 2,500	Lower greenschist	BIF	Qtz+mag+ank+stp+hem+Fe-talc+apa	5H-I, 6
				Ferruginous chert	Qtz+stp+ank+OM	4A
Pecors (SM0175_1383.5)	Kerr-McGee 156-1 core Depth: 1383.5m	Ca. 2,220 - 2,450	Greenschist	Pelite	Qtz+mus+chm+alb+py+rt+OM	2F-H, 3C-D, F
Tuomivaara (TU2-98.25)	TU2 drill core in the Kainuu schist belt of central Finland	Ca. 1,920 -2,000	Lower amphibolite	Pelite	Qtz+mus+bt+py+gra	2I-J
Pääkkö (RPK344-156.4)	Kainuu schist belt of central Finland Drill core #344 - M-52/3441/73/344	Ca. 1,920 -2,000	Lower amphibolite	Sulphidic iron formation	Qtz+mag+gru+py+apa	4B

Supplementary Table 2. Elemental analysis of selected minerals by energy dispersive spectroscopy. Totals accurate to ± 1%. Mus –muscovite, Par – parisite, Alb – albite, Gru – grunerite, Cal – calcite, Mnz – monazite, Ann – annite, Bt – biotite, Stp – stilpnomelane, Ank – ankerite.

Sample	Anshan						Pecors				Isua				Tuomivaara		Pääkko	Dales Gorge		Temagami	
Mineral	Ms	Par	Chm	Alb	Gru	Cal	Mnz	Par	Ms	Chm	Ms	Chm	Ann	Act	Bt	Ms	Gru	Stp	Ank	Ank	Fe-talc
Element																					
C		11.1				12.9		12.2		39.3	49.5	37.7							13.9	15.7	
O	43.7	27.0	40.9	46.1	35.3	46.6	38.6	41.2	45.1				38.5	47.0	42.7	46.7	34.0	39.5	41.9	38.9	42.7
F		6.8						9.5													
Na				6.8																	
Mg			4.4		5.4				1.9	7.7		3.6	2.2	9.7	9.7	1.6	6.0	7.9	7.3	4.6	3.3
Al	20.6		13.6	12.4					18.3	13.1	17.6	13.3	10.1		10.8	18.3		2.6			
Si	25.1		12.5	33.3	25.5				21.3	13.1	22.0	12.3	16.6	25.7	19.1	24.1	26.4	26.2			27.5
P							11.4														
K	9.2								7.7		8.1		7.5		8.0	9.4		1.6			
Ca		6.2		1.4		39.1	1.0	9.7						8.9					22.7	20.2	
Ti													1.3		1.3						
Fe	1.4		28.6		33.8	1.4		1.6	5.8	26.8	2.8	33.1	23.9	8.7	8.3		33.6	22.2	14.2	18.7	26.5
Mn																				1.9	
La		12.9					14.2	7.6													
Ce		26.0					26.7	11.9													
Nd		5.8					7.9	6.4													
Th		4.2																			
Total	100.0	100.0	100.0	100.0	100.0	100.0	100.0	100.0	100.0	100.0	100.0	100.0	100.0	100.0	100.0	100.0	100.0	100.0	100.0	100.0	100.0

Supplementary Table 3 Stable isotope compositions of OM and carbonate in bulk rock powders of samples in this study.

**Sample**	**TOC %**	**$\delta^{13}C_{org}$ (VPDB) ‰**	**TIC %**	**$\delta^{13}C_{Carb}$ (VPBD) ‰**	**$\delta^{18}O_{Carb}$ (SMOW) ‰**
Isua metapelite	0.1 to 8.8[1]<br>Ave. 1.36[1]	-12.5 to -23.8[1]<br>Ave. -17.8[1]	-	-1.4[2]<br>-2.7[2]	11.8[2]<br>17.0[2]
(GRDP0402 – this study only)	-	-21.8	-	-	-
Isua BIF (GRDP0404)	0.04	-26.9	Bdl	Bdl	Bdl
Anshan metapelite (ANS0904)	-	-23.4	Bdl	Bdl	Bdl
Anshan BIF (ANS0917)[3]	0.03	-26.7	3.0	-8.2	14.5
Temagami BIF (TE0704)	0.04[3]	-27.8[3]	1.5	-4.6[3]	15.7[3]
Dales Gorge BIF (DGM-1-127-5)	0.03	-24.7	25.5	-8.8	19.2
Dales Gorge BIF (DGM-1-169'6)	0.01	-24.8	5.4	-10.5	20.5
Dales Gorge BIF (DGM-1-198'6)	0.03	-25.2	10.1	-10.3	19.9
Dales Gorge BIF (DGM-1-249)	0.02	-23.6	4.8	-10.5	20.6
Dales Gorge BIF (DGM-1-273-10)	0.05	-24.0	2.9	-8.0	19.7
Pecors metapelite [4]<br>*(SM0175_1383.5 – this study only)*	0.03 to 0.87<br>Ave. 0.46	-26.6 to -40.5<br>Ave. -34.6	-	-	-
Pääkkö IF (RPK344-156.4)[3]	1.54	-19.6	13.3	-11.7	18.8
Tuomivaara metapelite (TU2-98.25)	-	-23.8	Bdl	Bdl	Bdl

Where indicated data taken from [1]Ohtomo et al., 2013; [2]Oehler and Smith, 1977; [3]Dodd et al., 2019; [4]Bekker and Kaufman, 2007. Bdl – below detection limit.

Supplementary Table 4 Crystallisation temperatures for graphitic carbon in the samples studied, using the thermometer developed by Beyssac et al., 2002.

Sample	G-band position	G-band FWHM	D1-band position	D-band FWHM	2D-band position	2D-band FWHM	D1-band area	G+D2 band area	D/G intensity	2D/ G intensity	T estimate Beyssac ± 50°C
Pääkkö	1578	21.9	1351	39.9	2701	71.2	2565	6812	0.2	0.1	517
Tuomivaara	1583	35.6	1357	56.6	2698	86.0	1972	4009	0.3	0.2	491
Isua	1593	19.3	1363	42.7	2715	76.8	1845	3530	0.2	0.1	485
Anshan	1581	26.7	1353	43.4	2700	70.8	2424	3729	0.4	0.2	462
Pecors	1594	47.7	1360	44.1	2700	64.3	10497	6576	2.7	0.5	361
Temagami	1592	43.1	1359	42.9	2703	65.9	1200	681	1.6	0.1	351
Brockman	1601	50.4	1348	68.7	NR	NR	6917	3464	1.5	NR	338

Supplementary Table 5. Bulk geochemical data of BIF and pelite relevant to this study

	Averaged Compositions of BIF and Pelites relating to the samples of this study									
	Isua		Temagami	Anshan		Dales Gorge		Pecors	Pääkkö	Tuomivara
	BIF	Pelite	BIF	BIF	Pelite	BIF	Pelite	Pelite	Sulphidic pelitic IF	Pelite
Ref.	This study	Ohtomo et al., 2013	This study	Dai et al., 2014	Peng et al., 2018	Ewers and Morris, 1989; Pecoits et al., 2009	Kurzweil et al., 2015; Alibert and McCulloch, 1993	McLennan et al., 1978	Laajoki, 1975; Laajoki and Saikkonen, 1977	Gehör and Havola, 1986
SiO_2	44.18	65.68	28.99	43.44	54.40	39.82	46.01	60.76	43.72	42.60
TiO_2	0.01	0.05	0.02	0.05	0.77	0.02	0.13	0.77	0.08	0.64
Al_2O_3	0.16	0.98	0.71	0.91	13.70	0.15	2.46	19.36	0.76	12.50
Fe_2O_3	56.01	19.64	66.74	51.86	21.53	47.55	31.21	6.60	14.34	33.10
FeO									22.24	
MgO	1.07	10.08	1.04	1.17	3.98	3.14	5.76	2.58	4.05	4.00
CaO	0.75	0.40	2.09	1.80	2.50	2.23	2.03	0.63	4.42	1.36
Na_2O	0.01	0.04	0.17	0.04	0.35	0.02	0.09	1.54	0.11	0.00
K_2O		0.04	0.05	0.03	0.35	0.03	0.97	3.90	0.13	3.50
MnO	0.06	0.14	0.04	0.05	0.26	0.02		0.05	0.04	0.09
P_2O_5	0.02	0.03	0.14	0.15	0.17	0.30	0.25	0.10	1.45	0.21
S						0.14	0.24		1.09	
LOI		3.26		0.33	1.78	4.77	9.66	3.92	2.05	
CO_2						5.26	6.70			
Total	102.27	100.00	99.98	99.81	99.80	98.38	98.79	100.21	100.00	98.10
TOC	0.04	1.36	0.04	0.03		0.03	2.50	0.46	1.54	
Sc		2.46		1.27	16.50	0.44	14.30			
V	50.29	20.51	53.20	12.47	122.00	1.13	128.00			
Cr	4.53	46.12	2.97	279.50	198.67	2.25	150.00			
Co	5.71	6.21	3.58	5.18	36.27	0.45	28.30			
Ni	68.88	52.78	39.36	5.87	30.67	1.42	90.70			
Cu	5.63	10.47	1.31	6.90	514.00	1.57	72.30			
Zn	34.04	57.53	13.25	42.98	90.70	9.37	129.00			
Ga	22.88	5.28	8.34	2.28	15.10	0.14	18.80			
Rb	3.75	1.50	4.14	0.38	123.63	1.77	195.00			
Sr	5.35	9.00	84.71	7.56	83.23	5.30	15.20			
Zr	1.64	15.80	8.57	0.48	22.53	2.08	108.00			
Nb	0.49	0.80	0.47	0.32	153.33	0.10	10.40			
Mo		1.09				0.32				
Sn	1.54	1.25	1.18			0.37				
Sb		2.04				0.32				
Cs			0.33	0.13	5.62	0.32	6.05			
Ba	1.22	3.58	3.54	4.79	20.98	20.86	213.00			
La	1.08	4.83	2.77	1.60	280.20	0.79	35.90			
Ce	2.09	9.03	5.44	2.72	11.53	1.21	68.10			
Pr	0.27	1.18	0.65	0.42	24.83	0.13	7.53			
Nd	1.19	4.23	3.06	1.91	3.37	0.56	26.20			
Sm	0.32	1.18	0.54	0.47	13.90	0.11	4.61			
Eu	0.26	0.80	0.40	0.25	3.29	0.05	1.03			
Gd	0.58	1.29	0.94	0.65	1.12	0.17	3.46			
Tb	0.09	0.32	0.11	0.12	3.56	0.03	0.50			
Dy	0.61	1.57	0.63	0.91	0.64	0.20	2.96			
Y	6.43	13.22	7.20	6.52	4.21	0.05	18.40			
Ho	0.39	0.41	0.37	0.22	0.91	0.18	0.56			
Er		1.08		0.66	2.74	0.03	1.72			
Tm	0.35	0.18	0.38	0.10	0.42	0.25	0.28			
Yb		1.01		0.66	2.81	0.05	1.78			
Lu		0.16	0.41	0.10	0.44	0.16	0.28			
Hf	0.18	0.61	0.44	0.04	3.29		2.60			
Ta	0.33	0.19	0.44	0.02	0.40		0.83			
W		0.70				0.62				
Tl				0.05	0.90					
Pb	1377.97	7.00		0.34	3.67	0.37	8.26			
Bi		2.71		0.07	2.70					
Th		0.59	0.50	0.10	0.96	0.06	13.03			
U		0.16		0.20	0.57	0.04	2.74			

Supplementary Table 6 List of selected clay minerals and their chemistry in BIF and pelite

Clay minerals in weakly metamorphosed BIF	Chemical formula	Clay minerals in weakly metamorphosed pelite	Chemical formula
Greenalite	$Fe_3^{2+}Si_2O_5(OH)_4$	Kaolinite	$Al_2Si_2O_5(OH)_4$
Stilpnomelane	$(K,Ca,Na)(Fe^{2+},Mg,Al)_8(Si,Al)_{12}(O,OH)_{36}\cdot nH_2O$	Montromillite	$(Na,Ca)_{0.33}(Al,Mg)_2Si_4O_{10}(OH)_2\cdot nH_2O$
Minnesotaite (Fe-talc)	$Fe_3^{2+}Si_4O_{10}(OH)_2$	Chamosite	$(Fe^{2+}, Al)_6(Si, Al)_4O_{10}(OH)_8$
Chamosite	$(Fe^{2+}, Al)_6(Si, Al)_4O_{10}(OH)_8$	Nontronite	$(CaO_{0.5},Na)_{0.3}Fe^{3+}{}_2(Si,Al)_4O_{10}(OH)_2\cdot nH_2O$
Ripidolite	$(Mg,Fe,Al)_6(Al,Si)_4O_{10}(OH)_8$	Illite	$(K,H_3O)(Al,Mg,Fe)_2(Si,Al)_4O_{10}[(OH)_2,(H_2O)$
Riebeckite	$Na_2(Fe^{2+},Mg)_3Fe_2^{3+}Si_8O_{22}(OH)_2$		
Ferri-annite	$K_2(Mg, Fe^{2+})_6Fe_2^{3+}Si_6O_{22}(OH)_4$		
Metamorphic successors			
Cummingtonite-Grunerite	$(Mg,Fe^{2+})_2(Mg,Fe^{2+})_5Si_8O_{22}(OH)_2$	Biotite	$K(Mg,Fe)_3(AlSi_3)O_{10}(OH)_2$
Ferroactinolite-tremolite	$Ca_2(Mg, Fe^{2+})Si_8O_{22}(OH)_2$	Muscovite	$KAl_2(AlSi_3)O_{10}(OH)_2$

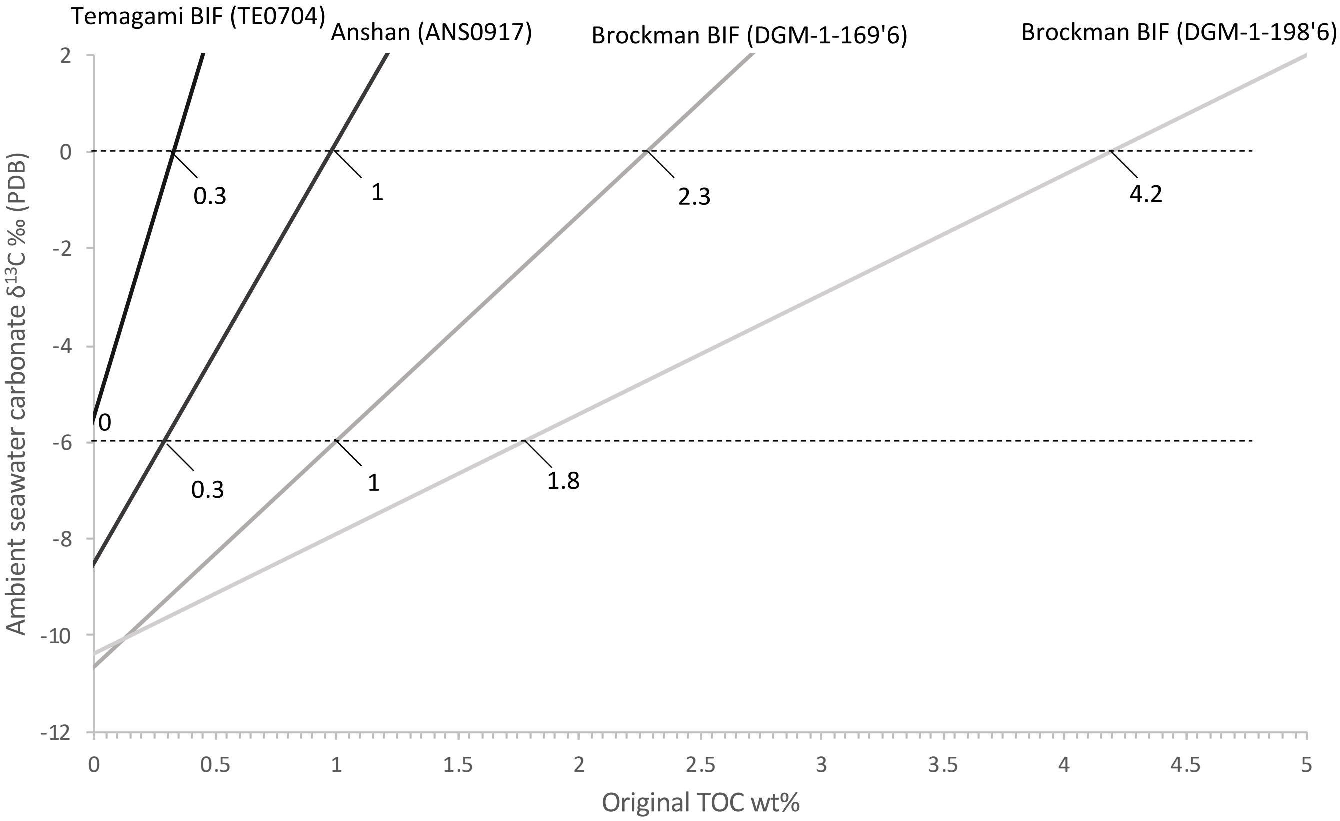

Supplementary Figure. 1 Isotopic mass balance model for the original TOC in BIF as a function of seawater carbonate $\delta^{13}C$ composition. Total inorganic carbon and carbonate $\delta^{13}C$ values used for the model are found in Supplementary Table 3.

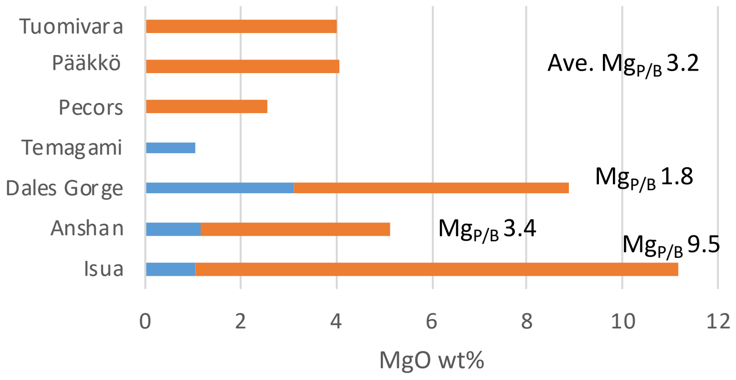

Supplementary Figure 2. Bulk rock MgO for BIF (B) and pelite (P) in this study. MgO here is used as a proxy to compare clay content in BIF and pelite.

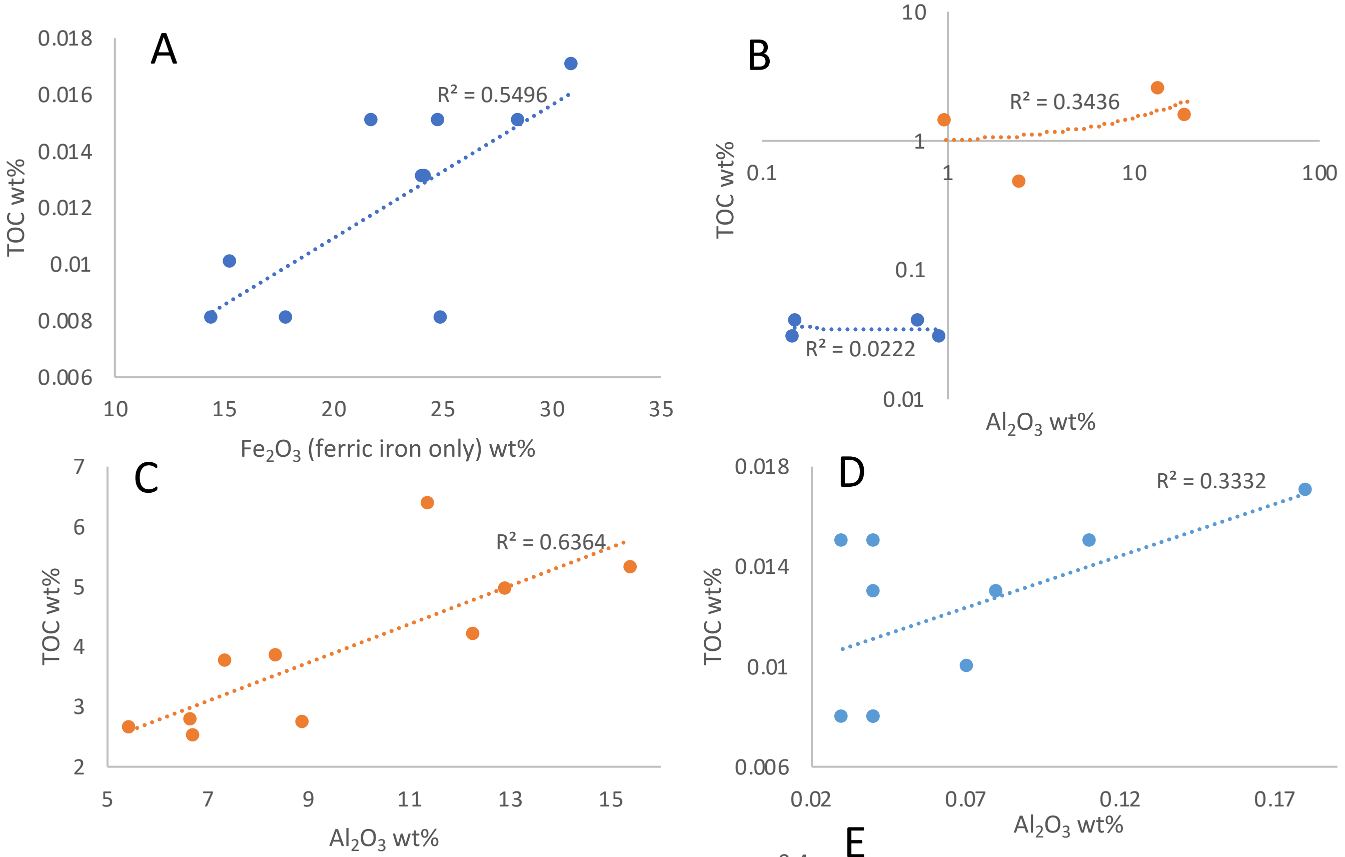

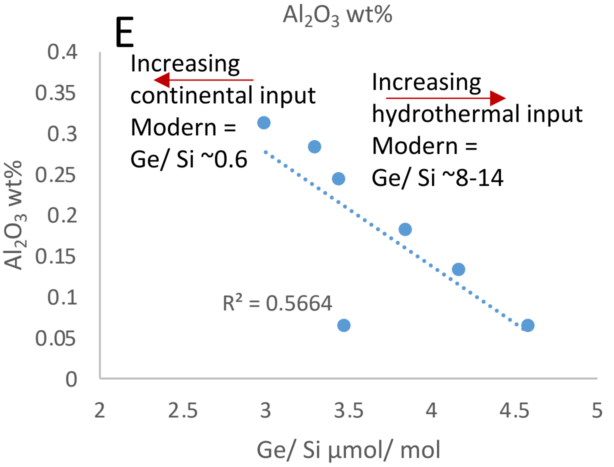

Supplementary Figure 3. Cross-plot data for selected elements in BIF (blue) and pelite (orange). A) TOC vs. ferric iron in BIF from the Transvaal supergroup . B) TOC vs. Al in BIF and pelite from data in this study. C) TOC vs. Al in pelite from the Transvaal supergroup D) TOC vs. Al in BIF from the Transvaal supergroup E) Al vs. Ge/ Si in silica-rich layers from BIF in the Mozaan group taken from Delvigne et al., 2012. All data from Transvaal supergroup taken from Klein and Beukes, 1989.

Supplementary References

1 Fernandez, A., van Dijk, J., Müller, I. A. & Bernasconi, S. M. Siderite acid fractionation factors for sealed and open vessel digestions at 70 ° C and 100 ° C. *Chemical Geology* **444**, 180-186, doi:10.1016/j.chemgeo.2016.10.015 (2016).
2 Rosenbaum, J. & Sheppard, S. M. F. An isotopic study of siderites, dolomites and ankerites at high temperatures. *Geochim Cosmochim Ac* **50**, 1147-1150, doi:https://doi.org/10.1016/0016-7037(86)90396-0 (1986).
3 Nutman, A. P., Allaart, J. H., Bridgwater, D., Dimroth, E. & Rosing, M. Stratigraphic and geochemical evidence for the depositional environment of the early archaean isua supracrustal belt, southern west greenland. *Precambrian Research* **25**, 365-396, doi:https://doi.org/10.1016/0301-9268(84)90010-X (1984).
4 Ohtomo, Y., Kakegawa, T., Ishida, A., Nagase, T. & Rosing, M. T. Evidence for biogenic graphite in early Archaean Isua metasedimentary rocks. *Nature Geoscience* **7**, 25-28, doi:10.1038/ngeo2025 (2013).
5 Nutman, A. P., Friend, C. R. L. & Paxton, S. Detrital zircon sedimentary provenance ages for the Eoarchaean Isua supracrustal belt southern West Greenland: Juxtaposition of an imbricated ca. 3700 Ma juvenile arc against an older complex with 3920-3760 Ma components. *Precambrian Research* **172**, 212-233 (2009).
6 Boak, J. L. & Dymek, R. F. Metamorphism of the ca. 3800 Ma supracrustal rocks at Isua, West Greenland: implications for early Archaean crustal evolution. *Earth and Planetary Science Letters* **59**, 155-176, doi:https://doi.org/10.1016/0012-821X(82)90123-6 (1982).
7 Wang, C. *et al.* Changing provenance of late Neoarchean metasedimentary rocks in the Anshan-Benxi area, North China Craton: Implications for the tectonic setting of the world-class Dataigou banded iron formation. *Gondwana Research* **40**, 107-123, doi:https://doi.org/10.1016/j.gr.2016.08.010 (2016).
8 Dai, Y., Zhang, L., Wang, C. & Li, L. Chentaigou BIF iron mine in anshan and archean crustal accretion: zircon UPb age and Hf isotope constraints *Acta Geologica Sinica* **29**, 2537-2550 (2013).
9 Wan, Y. *et al.* Formation Age of BIF-Bearing Anshan Group Supracrustal Rocks in Anshan-Benxi Area: New Evidence from SHRIMP U-Pb Zircon Dating. *Journal of China University of Geosciences* **43**, 57-81 (2018).
10 Bau, M. & Alexander, B. W. Distribution of high field strength elements (Y, Zr, REE, Hf, Ta, Th, U) in adjacent magnetite and chert bands and in reference standards FeR-3 and FeR-4 from the Temagami iron-formation, Canada, and the redox level of the Neoarchean ocean. *Precambrian Research* **174**, 337-346, doi:10.1016/j.precamres.2009.08.007 (2009).
11 Bowins, R. J. & Heaman, L. M. Age and timing of igneous activity in the Temagami greenstone belt, Ontario: a preliminary report. *Canadian Journal of Earth Sciences* **28**, 1873-1876, doi:10.1139/e91-167 (1991).
12 Trendall, A. F., Compston, W., Nelson, D. R., De Laeter, J. R. & Bennett, V. C. SHRIMP zircon ages constraining the depositional chronology of the Hamersley Group, Western Australia*. *Australian Journal of Earth Sciences* **51**, 621-644, doi:10.1111/j.1400-0952.2004.01082.x (2004).
13 Ewers, W. E. & Morris, R. C. Studies of the Dales Gorge Member of the Brockman Iron Formation, Western Australia. *Econ Geol* **76**, 1929-1953, doi:10.2113/gsecongeo.76.7.1929 (1981).
14 Kaufman, A. J., Hayes, J. M. & Klein, C. Primary and diagenetic controls of isotopic compositions of iron-formation carbonates. *Geochim Cosmochim Ac* **54**, 3461-3473, doi:http://dx.doi.org/10.1016/0016-7037(90)90298-Y (1990).
15 Morris, R. C. Genetic modelling for banded iron-formation of the Hamersley Group, Pilbara Craton, Western Australia. *Precambrian Research* **60**, 243-286, doi:https://doi.org/10.1016/0301-9268(93)90051-3 (1993).
16 Ketchum, K. Y., Heaman, L. M., Bennett, G. & Hughes, D. J. Age, petrogenesis and tectonic setting of the Thessalon volcanic rocks, Huronian Supergroup, Canada. *Precambrian Research* **233**, 144-172, doi:https://doi.org/10.1016/j.precamres.2013.04.009 (2013).
17 Corfu, F. & Andrews, A. J. A U–Pb age for mineralized Nipissing diabase, Gowganda, Ontario. *Canadian Journal of Earth Sciences* **23**, 107-109, doi:10.1139/e86-011 (1986).
18 Young, G. M., Long, D. G. F., Fedo, C. M. & Nesbitt, H. W. Paleoproterozoic Huronian basin: product of a Wilson cycle punctuated by glaciations and a meteorite impact. *Sedimentary Geology* **141-142**, 233-254, doi:https://doi.org/10.1016/S0037-0738(01)00076-8 (2001).
19 Lahtinen, R., Huhma, H., Kontinen, A., Kohonen, J. & Sorjonen-Ward, P. New constraints for the source characteristics, deposition and age of the 2.1–1.9Ga metasedimentary cover at the western margin of the Karelian Province. *Precambrian Research* **176**, 77-93, doi:https://doi.org/10.1016/j.precamres.2009.10.001 (2010).
20 Laajoki, K. & Saikkonen, R. On the geology and geochemistry of the Precambrian iron formations in Väyrylänkylä, South Puolanka area, Finland *Bull. Geol. Soc. Finland* **292** (1977).
21 Hölttä, P. & Heilimo, E. in *Bedrock of Finland at the scale 1:1 000 000 - Major stratigraphic units, metamorphism and tectonic evolution* (ed Mikko Nironen) 77-128 (2017).
22 Kontinen, A. & Hanski, E. in *Mineral Deposits of Finland* (ed W.D: O'Brien Maier, H: Lahtinen, R) 557-607 (Elsevier, 2015).
23 Gehör, S. & Havola, M. in *Sedimentology of the Precambrian formations in eastern and northern Finland* Vol. 5 (ed Kauko Laajoki) (Geological survey of Finland, 1986).
24 Beyssac, O., Goffe, B., Chopin, C. & Rouzaud, J. N. Raman spectra of carbonaceous material in metasediments: a new geothermometer. *Journal of metamorphic geology* **20**, 859–871 (2002).
25 Oehler, D. Z. & Smith, J. W. Isotopic composition of reduced and oxidized carbon in early Archaean rocks from Isua, Greenland. *Precambrian Research* **5**, 221-228, doi:https://doi.org/10.1016/0301-9268(77)90029-8 (1977).

26 Dodd, M. S. *et al.* Widespread occurrences of variably crystalline 13C-depleted graphitic carbon in banded iron formations. *Earth and Planetary Science Letters* **512**, 163-174, doi:10.1016/j.epsl.2019.01.054 (2019).
27 Bekker, A. & Kaufman, A. J. Oxidative forcing of global climate change: A biogeochemical record across the oldest Paleoproterozoic ice age in North America. *Earth and Planetary Science Letters* **258**, 486-499, doi:https://doi.org/10.1016/j.epsl.2007.04.009 (2007).
28 Peng, Z., Wang, C., Tong, X., Zhang, L. & Zhang, B. Element geochemistry and neodymium isotope systematics of the Neoarchean banded iron formations in the Qingyuan greenstone belt, North China Craton. *Ore Geology Reviews* **102**, 562-584, doi:https://doi.org/10.1016/j.oregeorev.2018.09.008 (2018).
29 Kurzweil, F., Wille, M., Schoenberg, R., Taubald, H. & Van Kranendonk, M. J. Continuously increasing δ 98 Mo values in Neoarchean black shales and iron formations from the Hamersley Basin. *Geochim Cosmochim Ac* **164**, 523-542, doi:10.1016/j.gca.2015.05.009 (2015).
30 Alibert, C. & McCollom, M. T. Rare earth element and neodymium isotopic compositions of the banded iron-formations and associated shales from Hamersley, western Australia. *Geochim Cosmochim Ac* **57**, 187-204 (1993).
31 McLennan, S. M., Fryer, B. J. & Young, G. M. Rare earth elements in Huronian (Lower Proterozoic) sedimentary rocks: composition and evolution of the post-Kenoran upper crust. *Geochim Cosmochim Ac* **43**, 375-388 (1978).
32 Delvigne, C., Cardinal, D., Hofmann, A. & André, L. Stratigraphic changes of Ge/Si, REE+Y and silicon isotopes as insights into the deposition of a Mesoarchaean banded iron formation. *Earth and Planetary Science Letters* **355-356**, 109-118, doi:https://doi.org/10.1016/j.epsl.2012.07.035 (2012).
33 Klein, C. & Beukes, N. J. Geochemistry and Sedimentology of a Facies Transition from Limestone to Iron-Formation Deposition in the Early Proterozoic Transvaal Supergroup, South-Africa. *Econ Geol* **84**, 1733-1774 (1989).
