# Supplementary material for: Minimal biomass deposition in banded iron formations inferred from organic matter and clay relationships
Source: Nat Commun. 2019 Nov 4;10:5022. doi: 10.1038/s41467-019-12975-z (PMC6828686; doi:10.1038/s41467-019-12975-z)
Supplement: Supplementary file 2 — Peer review [file 41467_2019_12975_MOESM2_ESM.pdf]

Review round 1

Reviewer #1 (Remarks to the Author):

1.11 The authors examine the depositional link between organic matter (OM) and Fe silicates in metamorphosed shales and banded iron formations (BIFs). In doing so the authors seek to evaluate the widely accepted hypothesis that the lack of organic carbon observed in present day BIFs is the result of dissimilatory iron reduction (DIR) – generating the siderite and magnetite observed in modern BIF. Instead, the authors conclude that the OM inventory during BIF deposition was too low to support significant DIR and that this would have limited the nutrients recycled into the water column. This interpretation comes from the fact that OM deposited with shales was insulated from reductive processes through its association with Fe-silicates, so an absence of organic carbon with clays in BIF must mean it was never present in the precursor sediment. Their model also implies then that the primary iron minerals in BIF must have initially been deposited as ferrous iron-bearing minerals.

We thank the reviewer for taking the time to write this detailed review. The above synopsis is accurate and well-written, and we would here address two small points:

1) **Near-complete organic oxidation in BIF has been a popular model (Konhauser et al., 2005), and we agree in this study that organic oxidation did occur in BIF, yet the amount of OM deposited in BIF was likely low and therefore any organic oxidation removed the original small amounts of OM, thus limiting iron-reductive cycling. There are some other recent papers which suggest the abundance of OM in BIF could have been low (Kurzweil et al., 2015a; Rasmussen and Muhling, 2018).**

2) **We maintain in our model that the initial BIF sediment comprised ferrous iron minerals (such as Fe-clays) but also ferric iron minerals which may have formed via one of a multitude of possible pathways including free O2 oxidation, photoferrotrophs, or UV photolysis (Figure 9)**

1.12 General Comments: This manuscript puts an interesting twist on the primary and secondary mechanisms of BIF deposition. In the case of the former they argue that there was a lack of primary productivity in the Archean oceans, and thus BIF may have started out as ferrous silicate-rich sediment.

We appreciate the reviewer's acknowledgment of this works novelty. We would not posit for purely ferrous silicate muds, as iron-oxides must be accounted for. Some have suggested iron-oxides are secondary (Rasmussen et al., 2017), however we would suggest a portion is likely primary given the plethora of possible mechanisms available for iron oxidation.

We would not go as far as saying the entire Archean ocean suffered low productivity, however BIF are generally believed to have been deposited in deep basins, far from coastal settings, which are

low productivity environments today. Today productivity is 5-6 times that of the open ocean due to upwelling of nutrients along coastlines and nutrient runoff from the continents. However, the capture of phosphorus by iron-oxides in BIF may have prevented the return of some of these nutrients to shallow waters in the Archean.

1.13 In the case of the latter, the lack of primary ferric iron means that DIR was not an important diagenetic reaction. My issue with this model is not whether it is correct or not, but the lack of actual evidence presented here for a paradigm shift in our thinking. The carbon and iron isotopes in BIF, along with some more recent trace metal isotopes (e.g., Mo, Cu), are supportive of deposition of Feoxyhydroxides or Fe(III)-silica gels and their subsequent reduction via DIR.

We agree with the reviewer there was a significant ferric iron component in BIF, we Also argue here that there was low deposition of OM in BIF which would limit DIR. Below we discuss the carbon, iron and trace metal isotopes in BIF which were highlighted by the reviewer:

Figure from Johnson et al., 2008, showing the iron isotopic composition of iron formations and shale in the Precambrian. Note BIF apart from those deposited at ca. 2.5Ga carry positive iron isotope enrichments.

As correctly stated by the reviewer iron isotope records in BIF have previously been used to argue for DIR in BIF. However, most values fall between -1‰ to +1‰, with shales having much more negative values down to -3‰. BIF generally have more positive δ^{56}Fe values and therefore iron isotopes do not appear to strongly support DIR being a dominant reaction. Alternatively, an iron reduction shuttle was proposed to explain some of the negative δ^{56}Fe values in BIF (Li et al., 2015) where shallower water sediments like shales, would release isotopically depleted iron which was transported to BIF sites.

We concur the low δ^{13}C values in BIF carbonates may be partially derived from oxidation of ^{13}C-depleted OM during iron reduction, however as detailed in the new Figure 7, if hydrothermal activity modified the ambient seawater carbon isotope reservoir, less OM would be needed to be oxidised to account for ^{13}C- depleted carbonate, however we emphasize that OM oxidation played a role in lowering BIF δ^{13}C values.

In this revision we also provide a new compilation of Eu/Eu* and δ^{13}C data in BIF to support our data and hypothesis that hydrothermal activity could modify carbon isotopes in BIF. Eu/Eu* is a commonly used proxy to assess the strength of mixing between hydrothermal fluids and seawater, therefore by comparing the relative strength of hydrothermal fluid mixing with δ^{13}C values in BIF we can ascertain the probability that hydrothermal fluids modified ocean and BIF

carbon isotopic chemistry. Eu/Eu* and δ^{13}C in BIF, show increasing Eu/Eu* values (greater hydrothermal influence) producing more negative δ^{13}C values. Such a trend supports the hypothesis that carbon sourced from hydrothermal fluids modified local seawater isotopic compositions. A similar process can be observed in modern hydrothermally influenced sediments, whereby sediments deposited closer to vent sites carry more pronounced Eu anomalies compared to those deposited further away (Olivarez and Owen, 1991). Additionally, in the modern ocean brine pools around hydrothermal vent sites carry dissolved carbonate with δ^{13}C values down to -7‰, mixing between this pool and seawater precipitates Fe-Mn carbonates with negative δ^{13}C values (Zierenberg and Shanks, 1988).

Additionally, recent work utilising Mo isotopic compositions and abundances in BIF, and coexisting shale, were given as evidence for little organic deposition in BIF (Kurzweil et al., 2015a). The study compared total and isotopic Mo trends in shales and coexisting BIF and found BIF had lower total Mo and lighter Mo isotopic values, interpreted to reflect predominantly iron binding of molybdate, with little isotopic effect from Mo binding with organic matter.

Also, in this revised version of the manuscript we provide new whole rock data and analysis of previously published data that show TOC is correlated with ferric iron content in BIF (Supplementary Fig. 3), this observation would argue against widespread DIR in BIF, which should display the opposite trend.

1.14 I would argue that the lack of preserved OM is not evidence that these processes did not occur.

The central theme of our work is not that an absence of OM is evidence DIR did not occur, it is the absence of clay associated OM. As recent studies of modern sediments have shown, OM is much more resistant to oxidation than previously thought (Estes et al., 2019), and therefore OM preservation in BIF must be given considerable contemplation. The sum of work on clay bound OM (Ahn et al., 1999; Curry et al., 2007; Kennedy et al., 2014; Konhauser and Urrutia, 1999b; McMahon et al., 2016; Playter et al., 2017; Wacey et al., 2014; Williams et al., 2005) suggests the abundance of clay minerals in BIF should favour the preservation of a significant amount of the initial OM, if there were significant quantities of original OM. In this revision we try to approach this quantitatively, by assessing the relative abundances of clay minerals in BIF and associated shale, and how this affects their relative TOC contents. On lines 317-331 we estimate BIF have around 31% less clay content than shales and therefore should have 31% less OM bound in clays if clays in both shales and BIF were suspended in an organic-rich water column for a period prior to their deposition (see point 1.16), thus allowing them to capture OM. The McRae shale which forms part of the Hamersley iron formation has an average of 2.5 wt% TOC (Kurzweil et al., 2015a), so

assuming most of the OM is bound in clays (which is what we have observed in the shales studied here), the associated iron formations should preserve 31% of this TOC giving 0.78wt% TOC, instead the actual TOC of the iron formations is 0.03wt% or 17 times lower.

1.15 Indeed, even in some of the author's thin sections there is OM preserved in the Dales Gorge BIF samples in close proximity to hematite and magnetite, suggesting the necessary conditions for DIR may have been met.

We agree several of our samples show local evidence supporting OM oxidation. These instances show the reactants and products co-occurring as one would expect. We agree iron-reduction and OM oxidation occurred in BIF, and we now show this in the model in Fig. 8. However, the absence of OM in clays in BIF, correlated $\delta^{13}C$ and Eu/Eu* values which indicate a hydrothermal influence over $\delta^{13}C$, and other published work using trace elements such as Mo to argue for low OM deposition in BIF, we argue that OM oxidation did occur but was limited by the small initial amounts of OM deposited in BIF. We highlight this observation now in the abstract to make clear limited iron-reduction is plausible.

1.16 In my opinion, there also seem to be two major inconsistencies in the model. The first is the comparison of mechanisms in the preservation of organic carbon in shales (with high clay content) to preservation of organic carbon in chemical sediments such as BIF, rocks with low clay content. Why would we expect BIF to preserve organics when they never had a primary clay component to them?

This is a good idea to compare the abundance of clays in both shale and BIF to accurately determine their role in OM preservation. We now provide new data to address this, please see point 1.14 for detailed discussion on this. Also, see the below schematic for mechanisms for OM-clay complexation in shale and BIF, which is illustrated in main text fig. 9.

Several groups have conducted experimental BIF genesis experiments and found Fe-clays readily form in seawater analogues (Halevy et al., 2017; Konhauser et al., 2007; Tosca et al., 2016), so there is strong support for primary precipitation of Fe-clays from seawater. Similarly, experiments trying to simulate BIF diagenesis when silica has been included, have so far failed to produce Fe-clays during simulated diagenetic reactions (Kohler et al., 2013; Posth et al., 2013), so the sum of experimental work currently support a primary seawater origin of Fe-clays in BIF. Additional observations have shown that Fe-silicates have petrographic and geochemical features consistent with them being primary precipitates (Johnson et al., 2018; Rasmussen et al., 2019; Rasmussen et al., 2017). Some Fe-silicates may have formed during diagenesis, which remains a plausible mechanism for some Fe-clays in BIF, but primary precipitation from seawater cannot be discounted based on the current literature.

Schematic figure illustrating OM complexation with clays in a hypothetical water column. Clay minerals formed from chemical weathering are exported from shallow to deep marine settings. OM produced in the water column is then complexed with clays and sinks to deposit on the seafloor as OM-clay aggregates. In BIF depositional settings ferrous iron reacts with dissolved silica to produce Fe-clays in the water column (see Tosca et al., 2016 for Fe-clay formation in seawater solution). OM then can complex with these precipitated clays to form OM-clay aggregates like those in shales. However, this is not observed in BIF, suggesting there was low concentrations of OM in the water column.

1.17 And, it is a red herring to mention the iron silicates in oxide facies BIF because they are diagenetic. In fact, the authors mention that in the Dales Gorge they analyzed the cherty layers with high OM relative to the "typical BIF layers" (L91-92).

Please see the previous point 1.16 regarding primary Fe-clays in BIF. Yes, we found Fe-clays occur with OM in chert layers between the Dales gorge BIF layers, these Fe-clays in the chert layers support and show that Fe-clays do indeed capture and preserve OM just like clays in shale. Unless one invokes all OM in BIF was produced within the sediments by a benthic microbial community, but even then microbes growing within the sediments still can become encased in clays and OM preserved, see (Konhauser and Urrutia, 1999b; Wacey et al., 2014) for detailed information.

1.18 Second, I'm confused about how the authors envision BIF forming in the first place. It is generally assumed that they required bacteria, so how would BIF form if there were no nutrients and hence primary productivity? However, the authors themselves acknowledge that photoferrotrophs tend to shed iron encrusted sheaths to avoid cellular death, an observation that could account for the observed lack of OM deposition with BIF.

There have been numerous models to explain the formation of BIF, which include abiotic and biotic mechanisms (Cairns-Smith, 1978; Konhauser et al., 2002; Rasmussen et al., 2017). One model does involve photoferrotrophs (Konhauser et al., 2002), and indeed their life-style does not promote deposition of large quantities of OM (Thompson et al., 2014). Therefore, if photoferrotrophs acted to separate iron and OM, this would be unfavourable for DIR in BIF, which require the close association of OM and ferric iron. Some Fe-oxides in BIF could also have formed via photoferrotrophy (Konhauser et al., 2002), UV-photo-oxidation of ferrous iron (Cairns-Smith, 1978), and green rust formation under low O2 conditions (Halevy et al., 2017). Therefore, in the model depicted in Fig. 8, we include a range of initial oxides and iron minerals.

1.19 Moreover, the biomass could have been recycled in the water column either through exposure to an oxygenic photosynthetic zone (likely more prevalent for younger formations) or DIR. The decomposition of OM and recycling of nutrients therefore need not be restricted to the sediment pile, especially if anoxygenic photosynthesizers were able to shed iron encrusted sheaths.

This is a valid point, and biomass could have been oxidised in the water column, however even in modern oxygenated water columns, where OM has to travel to great depths in pelagic environments there is still considerable deposition of OM to the sediment pile (Estes et al., 2019), so it is unlikely that less OM can reach the sediment pile in an anoxic ocean. But more importantly if this was the case, then this would support our observations because reduction in the water column would minimise OM deposition in the sediments and therefore the measured 40-50% of ferric iron in BIF sediments would remain unreduced therefore acting as a sink for phosphorus and other nutrients adsorbed to the oxides.

1.20 Line specific comments: L15-18: This reads as though all clays are created equal or that they have similar sorptive properties.

We agree different clays are better at selectively absorbing different molecules, but we have shown in this study that OM is adsorbed onto a diverse range of alumina-rich clays in shales (Fig. 2) and Fe-rich clays in BIF (Fig. 3). So, while the chemistries may differ, most (if not all) clays appear to adsorb and preserve organic matter.

1.21 The text also implies then that organic carbon content in clays is a direct proxy for primary productivity, but if that were true, then one could simply do a TOC measurement of clays through time to back out their amount of planktonic biomass. Obviously, it is more complicated than that, but perhaps this paper would have been better constrained to just looking at the metapelites through time and not comparing to BIF?

Yes, this is partly true, TOC and clay content do correlate in sediments. Although, this depends on the amount of clay available to bind organic carbon, and vice-versa. For instance, there could have been high productivity and little clay or detritus availability for organic matter to bind to, as a result a lot of organic matter could be left unprotected and become oxidised. So, it is likely a combination of the two. However BIF need to be comapred to other sedimetns which had more obvious evidence for the deposition of pelagic OM, in order to assess the organic carbon cycle in BIF, and the role iron and OM cycling played in sequestering nutrients and their effects on ocean productivity.

1.22 L19: So, do the authors support the notion that BIF started out as ferric iron-rich deposits? If so, how did the Fe(II) get oxidized in the absence of plankton?

Ferric iron deposition likely contributed to the formation of BIF as seen with heamatite-rich jasper-BIF, as well as considering the plethora of possible oxidation mechanisms, please see point 1.18. We would not suggest there was an absence of plankton overlying BIF depositional areas, but the abundance of such plankton was limited as shown by the low TOC of our BIF samples and mass balance considerations of carbonate content and source mixing. This is now more clearly

illustrated in our proposed model. For instance, increased primary productivity could lead to more ferric iron produced through either direct O_2 oxidation or photoferrotorphy. This leads to a greater drawdown of phosphorus by adsorption to ferric oxides thus, limiting productivity. This would lead to a decrease in the plankton abundance and therefore a decrease in the rate of ferric oxide formation and decrease rate of phosphorus drawdown. Thus, producing a negative feedback mechanism (Fig. 9). This may lead to fluctuating cycles of productivity.

1.23 L29-35: The Keil reference pertains to sediments rich in clay minerals, so yes, in terms of muds one would expect high C preservation. But BIF are not composed primarily of clay minerals.

We agree, BIF are not primarily muds, but may still contain up to 30% or even 80% clay as estimated for our samples (main text lines 282-292), therefore these clays should still be able to sequester a considerable amount of OM (see point 1.14). Previous studies have argued for a component of BIF having been Fe-rich muds formed by precipitating Fe-clays (Rasmussen et al., 2017). This is also supported by experimental work on Fe-clay formation, please see point 1.16.

1.24 As chemical sediments, they have very low Al content, so comparing BIF deposition to modern mud deposition is not appropriate. Where the authors are being unclear is what they mean by BIF having Fe-silicates, i.e., clays. In the oxide BIF bands, there is minimal iron silicates of detrital origin – they are instead diagenetic. Yes, in the S-bands, there are detrital or authigenic (e.g., greenalite) iron silicates, but these do not represent times of true BIF oxide deposition. The authors need to clean up this bit of text to be clear what kind of clay they are talking about, detrital vs. diagenetic. If the former, then they are discussing different environments of deposition (shales vs. BIF). If the latter, then the processes took place within the sediment after burial and thus has no direct link to water column.

We thank the reviewer for raising this point, it is indeed important to compare BIF and detrital sediments carefully. BIF have low concentrations of detrital minerals and low totals of Al, however Fe-clays in BIF could have formed in the water column, which is supported by experimental and observational work (Rasmussen et al., 2017; Tosca et al., 2016). Please see point 1.16 regarding a primary water column origin of Fe-clays in BIF. If clays were detrital or precipitated in the water column or in sediments, it does not change their ability to bind with OM. Therefore, while BIF and clay-rich shales were deposited in different environments, and elements which make up clays in shale are detrital, while those in BIF are water column precipitates, both would still have had clays in the overlying water column capable of OM sequestration. It is important we clarify the meaning of what detrital vs. diagenetic clays are. Detrital we take to mean weathered rock particulates; diagenetic clays are clays formed in the sediment pile during diagenesis. Clays in BIF would have acted like detrital clays, in that they deposited out of a water column once formed, like detrital

clays settling out of the water column due to buoyancy. The text on line 313 has been modified accordingly to: "Furthermore, while clays in BIF could have been water column or sedimentary precipitates, and clays in pelite detrital, this difference in origin still permits both clay-types being present in the water column, and therefore open to capture and preserve OM."

1.25 L38-42: Clays might preserve organic carbon, but is that true for ferric oxyhydroxides? I do not think so as there are many papers that have shown the rapid reactivity of ferric oxyhydroxides with organic carbon – see Posth et al (2013) in Chem. Geol. and a number of other papers coming out of Andreas Kappler's lab. The Curry reference is also not relevant to BIF as again it is about clay mineral protection, and in this case, animal organics. Quite frankly, the intermixing of muds and BIF is confusing.

The detailed work from Posth and Kappler's groups on diagenetic ferric oxyhydroxide reduction with organic matter, supports iron reduction with OM during diagenesis. However, at the same time there is a multitude of papers arguing for exactly the opposite trend. In modern sediments iron oxides have been proposed to be major preservers of OM (Adhikari and Yang, 2015; Lalonde et al., 2012; Ma et al., 2018; Wagai and Mayer, 2007). Lalonde et al., 2012 suggested 22% of OM in sediments is bound with iron oxides. Therefore, the prevailing effects that iron oxides have on OM preservation in sediments is unclear. We provide a test of this puzzle on a published data set of TOC and ferric and ferrous iron content in BIF. We found that TOC contents of BIF are positively correlated with increasing ferric iron content (Supplementary Fig. 4). This would imply ferric oxyhydroxides may have captured and preserved OM in the water column. High resolution Raman scans of haematite bands in the Dales gorge BIF show microscopic particles of OM associated with hematite (Fig. 6) which could support this conclusion.

1.26 L46-48: Comparing and contrasting the OM content of BIF and shales through time is a great idea, but as I mention above, in the context of them being different lithologies that formed in different environments.

We are grateful for this kind comment on the study. Yes, indeed we have taken measures to more accurately compare the two by including a table detailing the chemistry of clay minerals in BIF and shale (Supplementary Table 6). We also include a new section on lines 317-331 describing the clay contents in BIF and shale.

Section 2.1: I am curious as to why the authors did not analyze the Mount McRae Shale or Whaleback Shale Member of the Brockman as comparisons to the Dales Gorge BIF. For the other periods of time there seems to be BIF vs. metapelite analyses, so why not here?

We did not currently have these samples. However, we now present a compilation of published geochemical data for the Mount McRae shale and other shales from the Hamersley iron formation (Supplementary Table 5).

1.27 L122-125: I presume the stilpnomelane-rich layers in the Dales Gorge are not from the oxide BIF (i.e., not photo Fig 1N right core)?

All the sections analysed in the Dales Gorge BIF contained Fe-silicates to some degree. The stilpnomelane-rich layers occur between magnetite bands like the middle core section in Fig. 1N, which contains layers of magnetite, hematite and Fe-silicates.

1.28 L140: Seems like a discussion of phase specific REE analysis from BIF would be fitting here, or elsewhere. (i.e., Alibert, 2016 in GCA; Oonk et al., 2017 in Chem. Geol and 2018 in GCA).

We have removed the section on REE and sulphur cycling in pelite as it distracted from the focus of the study, following the suggestion of other reviewers. We have however included discussion of phase specific REE and its implications for our observations and model: [excerpt from main text] "Also, REE patterns of greenalite in the Hamersley BIF were interpreted to reflect a diagenetic origin for the Fe-silicate." And "Furthermore, REE patterns for stilpnomelane and minnesotaite in BIF from the Transvaal group were interpreted to reflect a primary seawater origin for these Fe-silicates."

1.29 L158-161: You cannot compare the Fe-oxide bound phosphorous flux to organic phosphorous flux in modern oceans to the Precambrian and stating that "the iron-oxide fluxes may have been higher in the Precambrian?" is rather obvious. Does anyone not think that BIF deposition required higher iron fluxes in the past?

Yes, indeed iron-oxide fluxes would have likely been higher, however this section has been removed as it distracted from the main conclusions of the work, please see previous point 1.28.

1.30 Section 3.2: This entire section makes the paper disjointed as suddenly the focus is on whether graphite was initially biological, and hence Isua is a record of the oldest biosphere.

We agree and but have removed these sections on REE, sulphur and biological graphite in pelite (see above points 1.28-1.29).

1.31 P194: is illite actually a detrital clay vs. authigenic, i.e., illitization of smectite?

Illite may be a secondary mineral, however it will transform to muscovite as written in this line Gharrabi et al., 1998. However, the mechanisms are not relevant here, this was part of the same section which was removed (see above points 1.28-1.30).

1.32 L216-217: The text: "Fe-silicates in BIF are believed to have formed from inorganic, chemical reactions of iron and silica in the Precambrian oceans" is based on a selective reading of the literature. Yes, Rasmussen et al. believe BIF started out as greenalite, but most of the community

disagrees because of the difficulties in oxidizing all the greenalite to ferric iron-bearing minerals post-depositionally. It also implicitly assumes that there was no plankton to oxidize dissolved Fe(II) to ferric oxyhydroxides. But if true, then how do you explain the organic carbon preserved in BIF or the ferruginous cherts (L225).

We agree it would be difficult to oxidise all the Fe-silicates post-depositionally to ferric iron, however this does not preclude deposition of both ferric oxyhydroxides and Fe-silicates. In our model we consider BIF forming from precipitation of ferric oxyhydroxides, Fe-silicates and Fe-carbonates collectively. Several groups have found Fe-silicates readily form in Precambrian seawater analogues supporting a primary origin of the Fe-clays, please see point 1.16. We consider a plankton presence overlying BIF depositional areas, please see point 1.22. However, another source of organic carbon in the BIF can be organic export from shallow marine areas. TOC in BIF is correlated with Al (Beukes et al., 1990) (Supplementary Fig. 4), which supports the possibility of transport of organic matter with detritus from productive continental shelfs.

1.33 L226-228: Again, if greenalite is diagenetic, then the relationship with organic carbon falls apart in terms of it being a primary water column productivity indicator.

Yes, if greenalite is diagenetic it would limit its ability to capture OM from the water column, but it may still capture OM in the sediments, similar to clays forming around microbes in sediments (Konhauser and Urrutia, 1999a) (see below figure). In the below figure diagenetic clays form around microbes through binding of positively charged cations such as Mg^{2+} and Fe^{2+} with negatively charged organic groups in organic matter and cell walls.

From Konhauser and Urrutia, 1999. TEM of a heavily mineralised epilithic bacterial cell, with abundant amorphous and poorly ordered grains of Fe-Al silicates on cell wall and within encompassing extracellular polymers. Scale bars320 nm.

1.34 L259-270: This is where the text seems to contradict earlier assertions. The authors admit that there was likely organic carbon deposited with the primary BIF sediment, so isn't the lack of organic carbon in the BIF now either an indicator of DIR or that it was not preserved. But it does not seem to me to suggest that it never existed in the first place, i.e., the model proposed here.

We agree OM did exist in the sediments, and it is still preserved in the sediments as indicated by our observations (Fig. 5). We now make clear in the model and abstract and conclusions, that OM was deposited in BIF. However, it is estimated BIF initially had 1-10 wt. % TOC, assuming OM oxidation is the sole source of $\delta^{13}C$ depletions in BIF (Konhauser et al., 2017) (Supplementary Figure 1). Here we argue the lack of OM preserved in Fe-clays within BIF suggests the initial amount of OM may have been much lower, and more similar to modern open-ocean sediments

which contain only 0.1-0.6 wt. % TOC (Emerson and Hedges, 1988). We still maintain that small amounts of OM were deposited in BIF. We also provide another alternative/ supporting mechanism ($\delta^{13}C$ depleted hydrothermal fluid mixing with seawater) to explain the low $\delta^{13}C$ in BIF, thus allowing for less OM oxidation (see lines 157-162).

1.35 L287: Isn't the statement: "If Fe-silicates formed diagenetically, it could occur through the reduction of ferric-oxides with OM oxidation" already what the community thinks happened?

Many members of the community likely believe so, but equally other groups (Konhauser; Tosca; Rasmussen) have shown and would suggest Fe-silicates are primary precipitates, please see 1.16. We have included discussion on the diagenetic origin of Fe-silicates by discussing how they may have formed this way and how this impacts OM preservation on lines 229-243).

1.36 L291-294: "Nevertheless, if Fe-silicates were forming during iron-reduction, the newly formed diagenetic clays would adsorb to, or encapsulate the remaining OM and/ or microbes, as is seen in modern microbial biofilms, preventing further OM oxidation, and therefore OM should be preserved within Fe-silicates in BIF". Two problems here. First, I don't see why the remaining C would be encapsulated by the diagenetic silicates because DIR consumes the organic C and the remaining C is bound to other minerals such as ferric oxyhydroxides.

It has been shown that microbes (or OM) in solution can be rapidly encased in clays (Playter et al., 2017), therefore before OM even reaches the sediments OM would be encased in clays. Nevertheless, even if OM reached the sediments without binding to any mineral surfaces, if there were microbes living in the sediments, they would be excreting and be sources of OM themselves. Because there are many functional groups in OM, such as COO^- or PO_3^- (Newcomb et al., 2017), OM in sediments are reactive sites which will bind with species such as Fe^{2+}, Si^{2+} and Mg^{2+}, allowing local supersaturation of these species and promote the diagenetic formation of Fe-clays. This trend has been observed in microbial cell cultures (Konhauser and Urrutia, 1999b). Please see image in point 1.33. Thus, even diagenetic clays could preserve OM, like primary precipitate clays in the water column.

1.37 Second, processes in biofilms cannot be equated to unconsolidated sediments unless the authors are arguing for the seafloor being covered in biofilms?

Yes, as seen in modern hydrothermal vent environments there are often various pockets of ferruginous and siliceous gel forming on pillow basalt (Edwards et al., 2010; Peng et al., 2011). However, if there was active DIR there had to be bacterial cells within the sediment pile. These cells would secrete extracellular polymeric substances like cells in a biofilm (albeit perhaps not in such great quantities), and therefore as described in point 1.36, these cells would act as sites for Fe-clay formation and OM preservation.

1.38 L295-298: The authors are arguing that all BIF layering is the same. The greenalite in oxide facies is likely diagenetic while that in the cherts or S-bands could be primary. But again, this is not oxide facies BIF per se.

We are grateful the reviewer raised this point.There are currently two models for the origin of greenalite in oxide facies BIF, one is a diagenetic origin and the other that greenalite is a water column precipitate, please see point 1.16 and 1.33. As the sum of experimental work has shown so far Fe-silicates can be formed in solution please see point 1.16. However, it may be possible to form some Fe-silicates during diagenetic reactions, but further experimental work will be needed to clarify the mechanisms behind this. If the clays were diagenetic or formed within the sediments only, then they may still capture organic matter, as can be seen by authigenic clays encapsulating microbes.

1.39 L3112-314: This sentence is only true if the iron silicates were formed in the water column.

Yes, we agree, and this can be supported by experimental and observational work (see point 1.16). But also, authigenic clay formation about microbes could also lead to OM clay associations (see point 1.33).

Figure 7: Magnetite is shown as a primary precipitate and not diagenetic. What is the evidence? Magnetite is also mentioned at the end of the discussion as being a derivative of green rust but that would require some primary ferric iron. Magnetite forming as a metamorphic mineral would require initial C and ferric iron, so I'm not sure why this is offered as a mechanism precluding carbon burial?

We do not argue that magnetite is a primary precipitate, we have removed this from the figure. Magnetite was written in the figure as a precipitate because it was proposed magnetite formed after green rust (Halevy et al., 2017). Also, magnetite has been hypothesised to form through the mixing of ferric iron produced in the upper water column and upwelling ferrous iron (Li et al., 2017). We include these possibilities of magnetite formation via these proposed mechanisms to be inclusive, however have now reworded the text in the figure to iron-oxyhydroxides for simplification.

1.40 L350-356: I am a bit confused about how the negative feedback works. If limited DIR means less nutrients to the water column, then biomass would decline. So, how did the BIF form unless you want to argue that all BIF started out as ferrous iron-bearing minerals. But you are not consistent in this view throughout the text.

Yes, this is right, less DIR would prevent the reduction of ferric iron and release of nutrients bound to this iron. This in turn would limit nutrient return to the water column, limiting primary productivity and therefore limit ferric iron production, if there was a biological control on ferric iron production. This would in turn limit the drawdown of nutrients by ferric iron. What this

means is any ecosystem in the sediments or overlying water would be self-limiting, i.e. if they become more active, they lead to more nutrients being removed from the water column, which will limit their activity. This model does not prevent plankton productivity, or remove the possibility for ferric iron formation, but demonstrates there is a negative feedback involved between primary productivity and BIF deposition which acts to regulate productivity.
We have addressed this comment by elaborating on the model in the closing paragraph of the discussion on line 387.

Reviewer #2 (Remarks to the Author):

2.11 This study explores the role of biology in the deposition of iron formation by analysing organic matter (OM) associated with clay minerals. OM associated with clay minerals should be protected from anaerobic degradation during early diagenesis, and as such, may provide a more accurate estimate of the original OM content of BIF than bulk sediment analysis. BIF genesis is controversial, and some theories suggest that OM content was originally high, but was consumed during ferric iron reduction, producing diagenetic ferrous minerals. In contrast, these new data suggest BIF deposition was decoupled from OM. The work relies on several major assumptions: i) that greenalite is associated with organic matter in a similar way to other clay minerals, and ii) that the grunerite and minnesotite are the metamorphic products of primary greenalite and stipnomelane, and iii) that the OM content has not been lost or altered during metamorphism. These weaknesses aside, I think this timely study takes a novel approach to tackling a long standing problem, and will be of interest to a wide audience. I support publication of this work in Nature Communications following some moderate revisions.
We are grateful for the support of the reviewer and their time taken reviewing the manuscript.
2.12 One of my major concerns is that the discussion in sections 3.1 and 3.2 on REE and C-S cycling and Earth's oldest organic matter don't fit well with the rest of the paper. There is a nice simple story here about the genesis of BIF, and these two sections are distracting and don't link to the abstract and introduction. They either need to be better integrated and justified throughout the manuscript, or removed.
We agree this section was not directly related to the main topic of the study and has been cut.

2.13 The authors conclude that the deposition of iron silicate minerals must be decoupled from OM deposition. They don't explicitly state whether they prefer a scenario where iron silicates formed around the vent, and were subsequently remobilised, or formed from the water column but

during intervals of higher exhalative activity. Figure 7 suggests the authors prefer the second scenario. Do these new data enable us to distinguish between these two depositional scenarios?

We would consider a model whereby iron silicates precipitate when the appropriate geochemical conditions are met, this could be around the vent sites or further away during upwelling of ferrous iron bearing seawater (Rasmussen et al., 2017). We agree that iron silicates would form during periods of elevated exhalite activity. We have modified the model in figure 8 to show Fe-clays forming in the water column during periods of higher exhalative activity.

2.14 Another implication of this work is that the ferrous minerals are primary, rather than formed through diagenetic reduction of ferric iron minerals. The flux of primary ferric minerals from surface waters must, by extension, have been lower than previous work has assumed. One consequence of this is that phosphate may have been more available in the water column. However, the authors only consider the impacts of lower recycling rates on P availability.

This is true if ferrous minerals constituted more of the original BIF precipitates than previously thought, then P drawdown would be less. What this means is if ferric iron formation was tied to biological productivity, then ferric iron P drawdown would be controlled by biological productivity. We thank the reviewer for pointing out this factor. We have now included this factor in the feedback model described in the last paragraph of the discussion on line 387.

2.15 These rocks have all been metamorphosed to greenschist-amphibolite facies. Could some of the very low ∂13C signatures discussed on lines 269-270 be altered? Have the ∂13C data been linked to petrography?

This is a good point, greenschist to amphibolite facies metamorphism could have caused a 1-3‰ decrease in carbonate δ^{13}C. We have included the following sentence on line 182: "Finally, the metamorphic grade of the BIFs studied here are all at or above the greenschist facies, therefore δ^{13}C could have been lowered by 1-3‰ due to metamorphic alteration.".

There have been previous attempts to link δ^{13}C values to different layers in some BIF (Baur et al., 1985). Baur et al., 1985 measured δ^{13}C values across iron-poor chert, iron-rich and carbonate layers in the Marra Mamba and Mt. Sylvia iron formations, the δ^{13}C values were rather homogenous between layers, however some showed significant differences between iron-rich and iron-poor layers.

2.16 There is some existing work that is not discussed but I believe supports the authors conclusions. Beukes et al. 1990 (p 682-683) suggest that initial OM content of BIFs was low based on Ba concentrations. See:

Beukes, N. J., Klein, C., Kaufman, A. J. & Hayes, J. M. Carbonate petrography, kerogen distribution,

and carbon and oxygen isotope variations in an early Proterozoic transition from limestone to iron-formation deposition, Transvaal Supergroup, South Africa. Econ. Geol. 85, 663–690 (1990).

We thank the reviewer for pointing out this important supporting study. We now cite this supporting work on line 295 and we have also identified and cited similar published work which uses Mo concentration and isotopic compositions to support low organic matter deposition in BIF (Kurzweil et al., 2015b).

Minor

2.17 Line 14 – 'my' should be by

This has been corrected.

2.18 Line 201 – 0.1-8% is a huge range. Is this for a range of minerals/layers?

This is between many different samples across the Isua belt (Ohtomo et al., 2012).

2.19 Line 311 – "around 0%" - What does this mean? Were all samples were below detection? It is difficult to describe a range as 'around' 0%, since you cannot go lower than zero.

True, since we detected no OM in the Fe-silicates we analysed we have reworded to appear as less than 1%.

2.20 Lines 276-344 – This is a very long paragraph, and difficult to follow. Please break it down.

It has now been split into three paragraphs.

2.21 Fig 1a – 'GRT' – is this meant to read GRU?

Grt represents garnet, we have added the abbreviation to the caption.

Reviewer #3 (Remarks to the Author):

3.11 The manuscript presents petrographic and isotopic data focusing on organic matter from a variety of metamorphosed mudstone (metapelite) and iron formation units of Archean to Paleoproterozoic age. The observations show that high amounts of organic matter is preserved in the pelites and associated with phyllosilicates, especially muscovite. In contrast, the phyllosilicates in the iron formations show no organic matter, with overall organic matter content lower in the iron formations. Any organic matter still present in the iron formations is associated with phosphates and carbonate. The authors continue to motivate a primary origin for organic matter in the pelites, and explains non-association of phyllosilicates and organic matter in iron formations to a decoupling of phyllosilicate and organic matter deposition, with the former happening more rapidly than the latter. They further argue for a limited role for coupled organic matter oxidation and iron reduction, leading to nutrient deficiencies in the overlying water column leading to negative feedback loops for

organic activity in early oceans.The manuscript is a carefully considered and generally well-balanced one and the author(s) need to be complimented for that. Testing well-established hypotheses that lack more detailed evidence is an important exercise, and most of the methodology employed here is appropriate for testing the relationship between organic matter and early Earth deep-water sediments. The data and observations reported in the manuscript is generally of a high standard and can add great value in the understanding of Archean to Paleoproterozoic iron formations and pelites. I also mostly to partially agree with some of the conclusions which include: the deposition of phyllosilicates was decoupled from that of organic matter in iron formations; the graphite in the pelites are of primary oprganic matter and not purely metamorphic origin; the negative δ13C values observed in iron formation carbonates cannot be exclusively linked to either a mantle or an organic matter source, and likely came from a combination of both. The model presented by the author(s) is indicating variable depositional rates of silicates, oxides and organic matter in iron formations is an eloquent one, although I have some concerns, which will be discussed in more detail below. The author(s) should also be complimented on section 3.1, which contains the strongest arguments in the manuscript in my opinion.

We are thankful to the reviewer for the time taken writing this complimentary review.

3.12 Interestingly, there appears to be an inverse correlation between δ13Ccarb and δ13COM in the studied samples. Have the author(s) considered assessing this further?

We thank the reviewer for pointing out this correlation between δ^{13}Ccarb and δ^{13}Corg. This is indeed an interesting trend, we attempted to seek validation of this trend with other published data from Beukes and Klein, 1990 (see below figures), however this published data did not produce a similar trend.

From Beukes and Klein, 1990, oxide facies iron formation and shale showed no significant trends while limestone and carbonate iron formation showed slightly positive correlations between δ13Ccarb and δ13Corg. Since data from Beukes and Klein, 1990 are more numerous and are from the same stratigraphic section (as opposed to our samples which span different time periods, basins and metamorphic grade), we cannot find conclusive evidence for this being a correlation in BIF.

Inorganic and organic carbon isotope values in BIF and associated sediments from BIF in this study and those from Beukes and Klein, 1990.

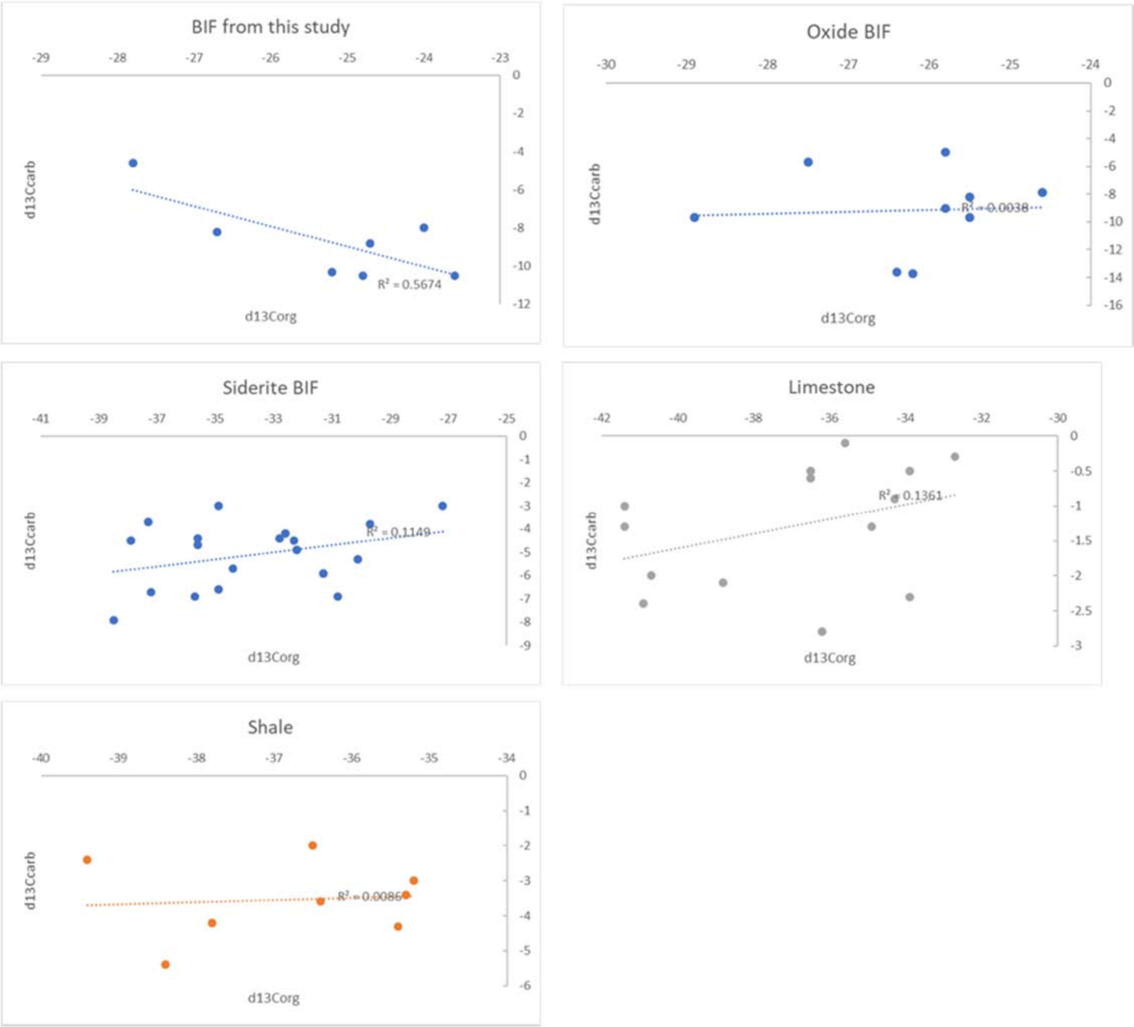

3.13 However, there are some facets where I have concerns. Four of them are major concerns and discussed here:

1. My first major concern with the manuscript relates to some contradicting data and arguments. With regards to the arguments, a lot of the content is so well-balanced and considered that it puts the conclusions and model into stark contrast with it. The author(s) show a direct observable link between organic matter oxidation (small spot of residual organic matter in the iron formations) and mineral phases that would be a by-product of this oxidation (e.g. ankerite and phosphates). Additionally, carbonate is a minor constituent in the metapelite, which is in stark contrast to the iron

formations, and the δ13C values reported are much higher in the metapelite than for many iron formations (lines 85-88). Therefore, to some extent, the author(s) have proven, and states, that the process of coupled iron reduction and organic matter oxidation did occur. However, the abstract, presented model and conclusions downplay or does not mention this. This, in my opinion, makes the abstract, model and conclusions not truly representative of the data and discussions presented in the manuscript. Also, it should be noted that inferring rapid deposition in iron formations is dangerous (lines 333-335) as the existing literature has not convincingly proved this as can be seen in the relatively wide geochronological error ranges.

We agree with the assessment made here. As evidenced by the Raman and petrographic images some OM was deposited in BIF therefore it was possible some OM oxidation occurred. We indeed provide evidence for small spots of local iron reduction and organic matter oxidation (Fig. 5). We have made clearer this observation by including description of this in the abstract and inclusion in the model description (Fig. 8) and conclusions to make a more complete representation of the data.

We have removed the geochronological dates due to these large error ranges and the depositional rates being unnecessary for the revised text.

3.14 2. A second concern is that the authors do not convince in their arguments that the organic matter in the metapelites are not detrital. The strong association of the organic matter with muscovite is suspicious, as muscovite requires aluminium to form, and aluminium is detritally sourced in marine sediments. The muscovite is also absent in the iron formations. The authors need to address this with stronger arguments as to why the organic matter in the metapelites are of a similar source to that still preserved (and oxidized) in the iron formations. One suggestion on testing this is presented in the next paragraph.

We agree this is an important factor to consider. The association of organic matter with muscovite in the metapelites could be due to two factors. 1) clay minerals (muscovite) and OM complexed in the water column (Playter et al., 2017) (see below figures) and were deposited in the sediments bound to one another; or 2) organic matter was exported from highly productive shallow marine environments, to pelite depositional sites, this would be coeval with detritus export from shallow marine areas therefore producing a positive correlation. Both processes were likely at play, which would result in a positive association of TOC with Al, or OM with muscovite. Most of the organic matter in sediments is sourced from primary production in the oceans, some organic matter may be detrital organic matter eroded from sediments. Detrital OM deposition would obviously increase TOC in detritally enriched regions, however this does not affect the model, because clay

and bacteria/ OM would still complex in the water column as per point 1 above. The detrital OM would add to total TOC, which may account for pelite having more detrital OM than BIF, but the lack of OM with clay in BIF still supports a lack of OM complexation with Fe-clay in BIF.

From Playter et al., 2017. Time course of representative experimental sedimentation tanks illustrating Synechococcus-clay deposition. Kaolinite and montmorillonite (50 g) were added to 1 L volume of cyanobacteria. Images include kaolinite-Synechococcus and montmorillonite-Synechococcus mixtures for comparison. Note that within 15 min, the kaolinite-Synechococcus mixture is largely settled, creating a pale green/grey mass at the bottom of the tank.

From Playter et al., 2017. Transmission electron microscope (TEM) images of kaolinite-Synechococcus mixtures showing clay encasing microbes

3.15 Stating that the muscovite can't be detrital because "the pelites are mature sediments" (line 202) is not satisfactory, especially when considering the higher aluminium and titanium contents in metapelites. Also, a few lines before that the author(s) suggest the muscovite formed "from recrystallization of detritial clays or muscovite" (lines 191-192), which is contradictory to the statement from line 202. Although the muscovite is likely a diagenetic to metamorphic product, the precursor phase(s) was detrital, and should be treated as such.

We believe there has been some confusion here arising from the wording on this line. In this section we were arguing that the graphite encased in muscovite in the Isua pelite was not weathered out of an older rock whole. I.e. an older rock type could have had crystals of muscovite with graphitic inclusions which were eroded from the rock and deposited in the Isua sediment pile. We have removed this section following the advice of some of the reviewers due to it disjointing the focus of the study. However, the word detrital should be clarified here, we agree aluminium which formed the clays in shale was detrital. Detrital organic matter is here suggested to mean kerogen like material eroded out of sediments, not organic matter formed in the water column. This section was removed per suggestions from the other reviewers.

3.16 3. A third concern is the author(s) to a large extent comparing the phyllosilicates in the metapelites to those in the iron formations with regards to their relationship with organic matter. The phyllosilicates in the metapelites are all aliminium-rich, and therefore must have a significant detrital source, whereas the phyllosilicates in the iron formations are either of chemical or diagenetic origin and are aluminium-poor. Additionally, the general presence of pyrite in the pelites also indicate very different depositional conditions. Not only was it more anoxic but potentially even euxenic. Comparisons between BIF and pelites are dangerous! The discussions on the relationship between phylosilicates and organic matter in these two very different rock-types should therefore

be more clearly decoupled in the discussions than what they are in the current version of the manuscript (e.g. lines 310-315).

We agree the two sediment types are different in nature, so we have provided new bulk rock geochemical data to better compare the two (Supplementary Table 5, Supplementary figures 2-3). However, the chemistry of the clays in BIF and pelite do not appear to affect the clays ability to bind with organic matter, this we have shown in Fig. 2 and 3. We agree clays had different origins in BIF and shale but they both exhibit capabilities to bind organic matter, and as long as both clay types are present in the water column they should both complex with organic matter in the water column, or even within the sediments around microbes (Konhauser and Urrutia, 1999b).

We agree the regular occurrence of pyrite in shale compared to BIF does tell of different conditions, however the presence of sulphides does appear to affect the OM inventory of BIF. For instance, the ca. 2.5Ga Wutai BIF from Northern China, has abundant sulphides with characteristically low TOC (0.05wt%), like other BIF (Dodd et al., 2019) (see below Fig). We therefore do not consider the presence of sulphide content of shale and BIF a detrimental factor in comparisons between BIF and shale. Both sediments would have been deposited out of anoxic water columns, however BIF could have had a higher proportion of oxidants, i.e. more ferric iron. As described in point 3.17, the difference in iron content between BIF and shale could have contributed to varying levels of OM oxidation however, likely did not make a significant difference in the measured TOC. Following this constructive comment, we have taken extra care to point out the differences between the two rock-types in the revised discussion on lines 275-323, in order to address their different formation mechanisms and potential to preserve OM.

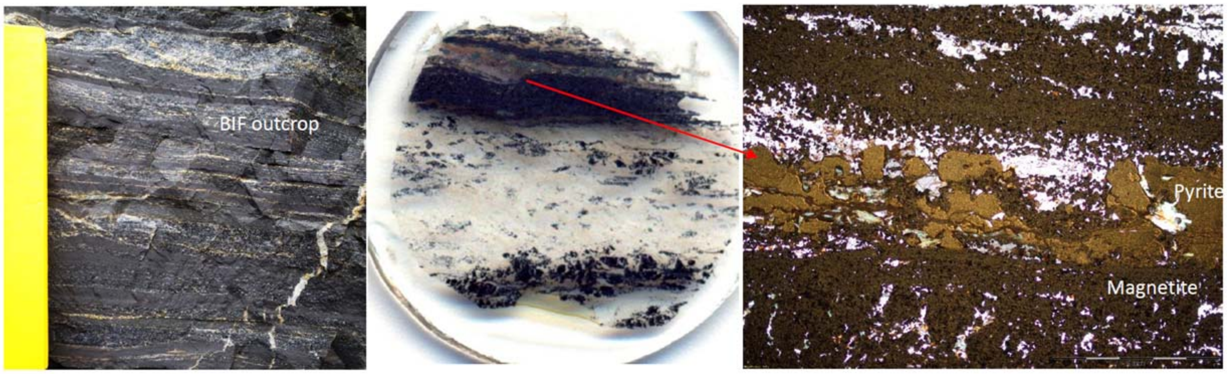

Wutai BIF showing abundant sulphides amongst magnetite layers in BIF

3.17 4. My final major concern with the manuscript in its current form is the comparison of the metapelites with the iron formation without the necessary whole rock geochemical characterization. This is especially important with regards to the total iron content in the metapelites in comparison

to the iron formations. The hypothesis tested here is testing a link between organic matter and iron redox, and with the metapelites generally containing more organic matter than the iron formations, the total iron content needs to be addressed, as well as the general abundance of ferrous and ferric minerals in the metapelite. The ability of the higher clay content in the metapelites to protect the organic matter from iron reduction-related decay can only be assessed if the nature and content of the iron in the metapelites compared to the iron formations have been addressed. It might be that there was much less ferric iron in the pelite depositional system when compared to the iron formation depositional system.

We are grateful for this helpful suggestion, we have added new whole rock geochemical data (Supplementary table 5 and supplementary fig. 2-3; Fig. 7,) and agree this geochemical characterization has greatly improved the study.

We have added the following text to line 297 and new figures (Fig. 6 and Supplementary Fig. 3) to assess the iron content of BIF and shale, and its implications for OM preservation: [main text excerpt] ***"The average iron oxidation state of BIF is estimated as $Fe^{+2.4}$ (Klein and Beukes, 1992), this means of the total iron in BIF on average 60% is ferrous and 40% is ferric iron, whereas the average oxidation state of iron in pelite before the Great Oxidation Event is 80% ferrous and 20% ferric iron (Bekker and Holland, 2012). Therefore, more ferric iron was available for iron reduction in BIF. However, the ferric iron content in BIF from the Transvaal supergroup, S. Africa, show a positive correlation with OM (Supplementary Figure 4A), which is the opposite trend expected for ferric iron reduction coupled to OM oxidation. In modern marine sediments iron is believed to be a 'rusty sink' and preserver of OM in the sediments, and OM bound to iron possibly accounts for up to 21% of the total OM in modern sediments (Lalonde et al., 2012). The observed occurrence of microscopic particles of OM and haematite in the Dales Gorge BIF (Fig. 6) may support this interpretation that ferric oxyhydroxides captured and preserved OM in BIF as opposed to removing OM via oxidative reactions."***

3.18 The authors also do not attempt to compare the abundances of clay minerals in the metapelite to that in the iron formations. In addition to iron, the total aluminium and titanium contents of the studied samples also need to be addressed, as this has an effect on the presence/formation of certain clay minerals (e.g. muscovite, stilpnomelane, chamosite instead of greenalite and minnesotaite) and is indicative of detrital content.

This is a good point, from petrographic estimates the pelite samples in this study contained 50-80% clay minerals, and BIF 5-80%, with the majority having a 20-30% Fe-clay content, which equates to BIF having 25-40% of the clay content of pelite. Alternatively, if we are to be more quantitative, we can use MgO as a proxy for clay content. Clays in BIF and shale have different

chemistries, i.e. BIF have Fe-clays predominately composed of Fe and Mg, and shale clays are Fe, Mg, Al, and K bearing, so we have to select common elements for fair comparison. Mg is a good choice and the average Mg content of clays in BIF and clays in shale are of a similar order around 4.4 and 6.5% respectively (Supplementary Table 2). We note however in BIF Mg may also constitute 10% of carbonate minerals, considering carbonate average around 5wt% in BIF, Mg in carbonate would shift total Mg by 0.5wt% of BIF. However, many Fe-clays in BIF may have no Mg and therefore bulk rock MgO may underestimate total clay content. The ratio of MgO in Shale/ BIF varies from 1.8 to 9.8 with an average of 3.2 (Supplementary Table 5; Supplementary Figure 2), so BIF have about 31% of the clay content of shales, which is consistent with petrographic estimates. Therefore, if BIF have about 31% the clay content of pelite, thenBIF should have 31% of the the clay-bound OM found in pelite. The McRae shale which forms part of the Hamersley iron formation has an average of 2.5 wt% TOC (Kurzweil et al., 2015a), so assuming most of the OM is bound in clays (which is what we have observed in the shales studied here), the associated iron formations should preserve 31% of this TOC giving 0.78wt% TOC, instead the actual TOC of the iron formations is 0.03wt% or 17 times lower.

3.19 A simple plot of total organic matter versus aluminium plus titanium will act as a first test to see if there is a link between detrital input and organic matter. If there is no correlation, it would strengthen the authors' arguments related to the non-detrital nature of the organic matter.

This is an important test which we have now included in supplementary information figure 3. We clarify here the adopted description for detrital and non-detrital organic matter: 1) detrital organic matter is kerogen-like material eroded from sediments; 2) non-detrital organic matter (or biomass in the water column), is formed by organisms living in the water column or sediments and ferruginous gels. We do not consider organic matter in shale or limestone as detrital, however they can show positive correlations with Al and or Ti (Beukes et al., 1990). The correlation between these elements can be due to Al-bearing clay or TiO_2 binding with OM in the water column or sediments (see point 3.14). Additionally, in distal shelf environments - where shale may have formed - OM may reach these areas as it is exported from shallow marine areas where detritus is enriched, whereby producing a correlation (see point 3.14 for description of the correlation between Al and OM). It is therefore expected that organic matter should correlate with detrital input because organic matter will be exported from productive shallow marine settings with detrital material from the continental shelves. Previous work by Beukes and Klein, 1990, show that in the Transvaal sedimentary successions shallow marine sediments have more detrital elements and more TOC, consistent with their proximity to shallow marine environments which experience higher weathering input and higher productivity. We have plotted TOC vs. Al in

BIF and shale from new and published data (Supplementary Fig. 3). New geochemical data of the BIF samples in this study show, no significant correlation between TOC and Al, whereas metapelite show a positive correlation. Using previously published data for the Transvaal group (Klein and Beukes, 1989) and (Kurzweil et al., 2015b), we find TOC and Al have a positive correlation in both shale and BIF. The positive correlation between Al and TOC in BIF and shale suggests a similar source of OM for both BIF and shale. If organic matter in shale and BIF was exported form shallow marine areas with high productivity this could explain the correlation between TOC and Al. As well as the binding of OM to Al-bearing clay minerals in shale.

3.20 The trace elements of the studied samples are also important with regards to the author(s) inferring a possible mantle/hydrothermal source for the 13C-depleted carbonates in iron formations, as rare earth elements, and Eu in particular, is indicative of hydrothermal input into a system. Therefore, if hydrothermalism is the more dominant control on δ13C in carbonates, a link should be observed to the trace elements.

This is a good idea, and we are grateful for this suggestion. We have followed through with the idea and produced a plot of Eu/Eu* (indicator for hydrothermal fluid influence e.g. (Grenne and Slack, 2003)) against $\delta^{13}C_{carb}$ using published data for BIF where the two variables have been measured in cohort on the same sample. There are surprisingly few studies which measure both variables in tandem, therefore we made use of the only two studies known to the authors, which provide these values (Smith et al., 2013; Teixeira et al., 2017). Plots of Eu/Eu* vs. $\delta^{13}C_{carb}$ in BIF samples from these studies show a correlation with increasing Eu/Eu* and decreasing $\delta^{13}C_{carb}$ (Fig. 7) which supports the possibility that hydrothermal fluid mixing with seawater modified the carbon isotopic chemistry of the mixed solutions.

Here are additional comments that also require attention:

3.21 • There are some typos and grammatical errors in the manuscript (e.g. using “my” instead of “by”; using “comprises of” instead of “comprises). Although not major or prevalent, these will need to be identified and corrected. Especially “comprised of” is used regularly in the manuscript and supplements, and will need to be corrected.

We thank the reviewer for highlighting these issues. The document has been checked and these typos and grammatical errors have been fixed.

3.22 • General comment: graphite is always associated with muscovite in the pelites, and muscovite is notoriously absent in clean BIF. This observation needs to be better addressed in the discussion.

This is true muscovite is the most common (silicate) clay mineral in the pelite here studied, however OM will bind with many different clay types e.g. (Ahn et al., 1999; Playter et al., 2017),

and even those in BIF such as stilpnomelane and grunerite (Fig. 3). We have included a table comparing clays (silicates) in BIF and pelite in the supplementary information and added text on lines 292-297 to address this difference.

3.23 • Line 67: With further reference to major concerns 3 and 4, rutile is concentrated in the OM layers. So is the OM of a detrital source? Can one say that the OM in pelite and that in BIF came from the same source and had the same available reactivities? Was it subjected to the same oxidants? Was ferric iron ever in the pelites?

The organic matter is not proposed to be detrital due to its association with detrital minerals. These minerals (clays/ rutile) can be transferred to the sediment by complexation with OM (Ahn et al., 1999; Curry et al., 2007; Erhayem and Sohn, 2014; Parnell, 2004; Playter et al., 2017; Thomas and Syres, 2012; Wang et al., 2014). Detrital OM is defined by the authors as OM eroded from sediments. Most OM in the pelite is likely derived from biomass synthesised in the ocean. From the observed trends in TOC vs. Al in BIF and pelite (Supplementary figure 4) it could be suggested OM was exported from the same source, (shallow marine areas) to depositional sites of both BIF and pelite. Although a positive correlation between ferric iron and OM in BIF suggests OM could also have been sourced from regions of ferric iron production (Supplementary figure 3). OM in BIF was possibly deposited with more ferric iron (see point 3.18) and therefore oxidants, but even with excess oxidants the protective capabilities of clays should still preserve OM-clay aggregates in BIF, if there was significant OM deposition in BIF.From our review of the literature we suggest ferric iron must have been originally present in pelite because they contain 20% ferric iron, likely they contained even more ferric iron, considering their high TOC must have led to some ferric iron reduction, which is supported by ^{56}Fe depletions in shale (see 1.13).

3.24 • Line 84-85: “syngenetic phase”. Be more specific. Syngenetic with what? Metamorphism? This is either a misuse of the term “syngenetic” or it requires better explanation.

We have removed this section on the syngenecity of graphite and OM in the Isua pelite, and its implications for early life, following the advice of other reviewers.

3.25 • Line 97-99: This IF has very strange characteristics such as feldspar and sulphide and organic matter. More characterization and whole rock geochemistry would be required to characterize it as a true IF. Also, grunerite indicates strong metamorphism as this is never seen in lower grade iron formations.

Yes indeed, this is a weird iron formation. It does contain 37 wt% Fe so would qualify as an iron formation in this respect (Fe >15 wt%). Perhaps a more appropriate name would be a sulphide-magnetite-bearing mudstone. The bulk rock geochemical characteristics are now present in

supplementary table 5. This sample was utilised in this study to make the case that grunerite preserves OM even at the amphibolite facies, so its lithological classification does not affect the conclusions.

3.26 • Line 119: Reference to a manuscript that is still in review is inappropriate in my opinion.

This manuscript is now published and cited here for references to carbon isotopic data in BIF used in this study.

3.27 • Line 155-161: The phosphorous cycle in modern oceans should not be used as a proxy for ancient oceans. The authors need to use more caution here.

This is the same section that has been removed (see point 3.24).

3.28 • Line 261: Also see Smith et al. (2013), Economic Geology, v. 108, 111-134.

Thank you for the reference, this citation has been added.

3.29 • Line 286-291: What about ferrous pore water from reduced ocean source causing magnetite formation? I.e. no organic matter was required anyway. Also, what about pH control on Fe-silicate formation (see Eh-pH diagrams by Brookins)?

This is a valid point and this reasoning was added to this section. Fe-silicates would require alkaline conditions see Tosca et al., 2016. We have added this to the description on line 235.

3.30 Line 353-356: The negative feedback loop at the end needs to be better explained. Very confusing! Also, what would be the result of limiting photoferrotrophy? Would it lead to decreased BIF deposition?

We have made a new figure to make the negative feedback cycle clearer (Fig. 9). The cycle would have limited the deposition of biologically formed ferric iron in BIF, so if BIF deposition was entirely controlled by biological activity this feedback could have reduced the rate of biological iron oxidation and iron deposition.

3.31 Reporting quantitative EDS data in supplementary materials is not acceptable and if it is not adding true value to the manuscript it should be removed.
We feel the EDS data is necessary for the comparison of clay chemistry in BIF and pelite, however the bulk of this data is not used in the manuscript and rather, we mostly refer to this data by the mineralogy. Hence this data is not critical to present in the main text and we believe it is supplementary information

3.32 To conclude, I would recommend major revisions to the manuscript, mostly related to the four major concerns listed in this review. I believe the data and many of the arguments to be of great value, and that a revised manuscript could warrant publication in Nature Communications where it will enjoy a large readership.
We thank the reviewer for their supporting words.

Adhikari, D., and Yang, Y., 2015, Selective stabilization of aliphatic organic carbon by iron oxide:

Scientific reports, v. 5, p. 11214-11214.
Ahn, J. H., Cho, M., and Buseck, P. R., 1999, Interstratification of carbonaceous material within illite: American Mineralogist, v. 84, p. 1967-1970.
Baur, M. E., Hayes, J. M., Studley, S. A., and Walter, M. R., 1985, Millimeter-Scale variations of stable isotope abundances in carbonates from banded iron-formations in the Hamersley Group of Western Australia: Economic Geology, v. 80, no. 270-282.
Bekker, A., and Holland, H. D., 2012, Oxygen overshoot and recovery during the early Paleoproterozoic: Earth and Planetary Science Letters, v. 317-318, no. Supplement C, p. 295-304.
Beukes, N. J., Klein, C., Kaufman, A. J., and Hayes, J. M., 1990, Carbonate Petrography, Kerogen Distribution, and Carbon and Oxygen Isotope Variations in an Early Proterozoic Transition from Limestone to Iron-Formation Deposition, Transvaal Supergroup, South-Africa: Economic Geology and the Bulletin of the Society of Economic Geologists, v. 85, no. 4, p. 663-690.
Cairns-Smith, A. G., 1978, Precambrian solution photochemistry, inverse segregation, and banded iron formations: Nature, v. 276, p. 807-808.
Curry, K. J., Bennett, R. H., Mayer, L. M., Curry, A., Abril, M., Biesiot, P. M., and Hulbert, M. H., 2007, Direct visualization of clay microfabric signatures driving organic matter preservation in fine-grained sediment: Geochimica et Cosmochimica Acta, v. 71, no. 7, p. 1709-1720.
Dodd, M. S., Papineau, D., She, Z.-B., Manikyamba, C., Wan, Y.-S., O'Neil, J., Karhu, J. A., Rizo, H., and Pirajno, F., 2019, Widespread occurrences of variably crystalline 13C-depleted graphitic carbon in banded iron formations: Earth and Planetary Science Letters, v. 512, p. 163-174.
Emerson, S., and Hedges, J. I., 1988, Processes controlling the organic carbon content of open ocean sediments: Paleoceanography, v. 3, no. 5, p. 621-634.
Erhayem, M., and Sohn, M., 2014, Stability studies for titanium dioxide nanoparticles upon adsorption of Suwannee River humic and fulvic acids and natural organic matter: Science of The Total Environment, v. 468-469, p. 249-257.
Estes, E. R., Pockalny, R., D'Hondt, S., Inagaki, F., Morono, Y., Murray, R. W., Nordlund, D., Spivack, A. J., Wankel, S. D., Xiao, N., and Hansel, C. M., 2019, Persistent organic matter in oxic subseafloor sediment: Nature Geoscience, v. 12, no. 2, p. 126-131.
Grenne, T., and Slack, J. F., 2003, Paleozoic and Mesozoic silica-rich seawater: Evidence from hematitic chert (jasper) deposits: Geology, v. 31, no. 4, p. 319-322.
Halevy, I., Alesker, M., Schuster, E. M., Popovitz-Biro, R., and Feldman, Y., 2017, A key role for green rust in the Precambrian oceans and the genesis of iron formations: Nature Geoscience, v. 10, no. 2, p. 135-139.
Johnson, J. E., Muhling, J. R., Cosmidis, J., Rasmussen, B., and Templeton, A. S., 2018, Low-Fe(III) Greenalite Was a Primary Mineral From Neoarchean Oceans: Geophysical Research Letters, v. 0, no. 0.
Kennedy, M. J., Löhr, S. C., Fraser, S. A., and Baruch, E. T., 2014, Direct evidence for organic carbon preservation as clay-organic nanocomposites in a Devonian black shale; from deposition to diagenesis: Earth and Planetary Science Letters, v. 388, no. Supplement C, p. 59-70.
Klein, C., and Beukes, N. J., 1989, Geochemistry and Sedimentology of a Facies Transition from Limestone to Iron-Formation Deposition in the Early Proterozoic Transvaal Supergroup, South-Africa: Economic Geology, v. 84, no. 7, p. 1733-1774.
Klein, C., and Beukes, N. J., 1992, Time Distribution, Stratigraphy, and Sedimentologic Setting, and Geochemistry of Precambrian Iron-Formations, *in* Schopf, J. W., ed., The Proterozoic Biosphere: A Multidisciplinary Study: Cambridge, UK, p. 139-147.
Kohler, I., Konhauser, K. O., Papineau, D., Bekker, A., and Kappler, A., 2013, Biological carbon precursor to diagenetic siderite with spherical structures in iron formations: Nat Commun, v. 4, p. 1741.
Konhauser, K. O., Amskold, L., Lalonde, S. V., Posth, N. R., Kappler, A., and Anbar, A., 2007, Decoupling photochemical Fe(II) oxidation from shallow-water BIF deposition: Earth and Planetary

Science Letters, v. 258, no. 1-2, p. 87-100.
Konhauser, K. O., Hamade, T., Raiswell, R., Morris, R. C., Grant Ferris, F., Southam, G., and Canfield, D. E., 2002, Could bacteria have formed the Precambrian banded iron formations?: Geology, v. 30, no. 12, p. 1079.
Konhauser, K. O., Newman, D. K., and Kappler, A., 2005, The potential significance of microbial Fe(III) reduction during deposition of Precambrian banded iron formations: Geobiology, v. 3, p. 167–177.
Konhauser, K. O., Planavsky, N. J., Hardisty, D. S., Robbins, L. J., Warchola, T. J., Haugaard, R., Lalonde, S. V., Partin, C. A., Oonk, P. B. H., Tsikos, H., Lyons, T. W., Bekker, A., and Johnson, C. M., 2017, Iron formations: A global record of Neoarchaean to Palaeoproterozoic environmental history: Earth-Science Reviews, v. 172, p. 140-177.
Konhauser, K. O., and Urrutia, M. M., 1999a, Bacterial clay authigenesis: a common biogeochemical process: Chemical Geology, v. 161, no. 4, p. 399-413.
Konhauser, K. O., and Urrutia, M. M., 1999b, Bacterial clay authigenesis: a common biogeochemical process: Chemical Geology, v. 161, p. 399-413.
Kurzweil, F., Wille, M., Schoenberg, R., Taubald, H., and Van Kranendonk, M. J., 2015a, Continuously increasing δ98Mo values in Neoarchean black shales and iron formations from the Hamersley Basin: Geochimica et Cosmochimica Acta, v. 164, p. 523-542.
-, 2015b, Continuously increasing δ 98 Mo values in Neoarchean black shales and iron formations from the Hamersley Basin: Geochimica et Cosmochimica Acta, v. 164, p. 523-542.
Lalonde, K., Mucci, A., Ouellet, A., and Gelinas, Y., 2012, Preservation of organic matter in sediments promoted by iron: Nature, v. 483, no. 7388, p. 198-200.
Li, W., Beard, B. L., and Johnson, C. M., 2015, Biologically recycled continental iron is a major component in banded iron formations: Proc Natl Acad Sci U S A.
Li, Y.-L., Konhauser, K. O., and Zhai, M., 2017, The formation of magnetite in the early Archean oceans: Earth and Planetary Science Letters, v. 466, p. 103-114.
Ma, W.-W., Zhu, M.-X., Yang, G.-P., and Li, T., 2018, Iron geochemistry and organic carbon preservation by iron (oxyhydr)oxides in surface sediments of the East China Sea and the south Yellow Sea: Journal of Marine Systems, v. 178, p. 62-74.
McMahon, S., Anderson, R. P., Saupe, E. E., and Briggs, D. E. G., 2016, Experimental evidence that clay inhibits bacterial decomposers: Implications for preservation of organic fossils: Geology, v. 44, no. 10, p. 867-870.
Newcomb, C. J., Qafoku, N. P., Grate, J. W., Bailey, V. L., and De Yoreo, J. J., 2017, Developing a molecular picture of soil organic matter-mineral interactions by quantifying organo-mineral binding: Nat Commun, v. 8, no. 1, p. 396.
Olivarez, A. M., and Owen, R. M., 1991, The europium anomaly of seawater: implications for fluvial versus hydrothermal REE inputs to the oceans: Chemical Geology, v. 92, no. 4, p. 317-328.
Parnell, J., 2004, Titanium mobilization by hydrocarbon fluids related to sill intrusion in a sedimentary sequence, Scotland: Ore Geology Reviews, v. 24, no. 1, p. 155-167.
Playter, T., Konhauser, K., Owttrim, G., Hodgson, C., Warchola, T., Mloszewska, A. M., Sutherland, B., Bekker, A., Zonneveld, J. P., Pemberton, S. G., and Gingras, M., 2017, Microbe-clay interactions as a mechanism for the preservation of organic matter and trace metal biosignatures in black shales: Chemical Geology, v. 459, p. 75-90.
Posth, N. R., Köhler, I., D. Swanner, E., Schröder, C., Wellmann, E., Binder, B., Konhauser, K. O., Neumann, U., Berthold, C., Nowak, M., and Kappler, A., 2013, Simulating Precambrian banded iron formation diagenesis: Chemical Geology, v. 362, p. 66-73.
Rasmussen, B., and Muhling, J. R., 2018, Making magnetite late again: Evidence for widespread magnetite growth by thermal decomposition of siderite in Hamersley banded iron formations: Precambrian Research, v. 306, p. 64-93.
Rasmussen, B., Muhling, J. R., and Fischer, W. W., 2019, Evidence from laminated chert in banded iron formations for deposition by gravitational settling of iron-silicate muds: Geology, v. 47,

no. 2, p. 167-170.
Rasmussen, B., Muhling, J. R., Suvorova, A., and Krapež, B., 2017, Greenalite precipitation linked to the deposition of banded iron formations downslope from a late Archean carbonate platform: Precambrian Research, v. 290, p. 49-62.
Smith, A. J. B., Beukes, N. J., and Gutzmer, J., 2013, The Composition and Depositional Environments of Mesoarchean Iron Formations of the West Rand Group of the Witwatersrand Supergroup, South Africa: Economic Geology, v. 108, no. 1, p. 111-134.
Teixeira, N. L., Caxito, F. A., Rosière, C. A., Pecoits, E., Vieira, L., Frei, R., Sial, A. N., and Poitrasson, F., 2017, Trace elements and isotope geochemistry (C, O, Fe, Cr) of the Cauê iron formation, Quadrilátero Ferrífero, Brazil: Evidence for widespread microbial dissimilatory iron reduction at the Archean/Paleoproterozoic transition: Precambrian Research, v. 298, p. 39-55.
Thomas, A. G., and Syres, K. L., 2012, Adsorption of organic molecules on rutile TiO2 and anatase TiO2 single crystal surfaces: Chemical Society Reviews, v. 41, no. 11, p. 4207-4217.
Thompson, K. J., Lliros, M., Michiels, C., Kenward, P., and Crowe, S., 2014, Pelagic photoferrotrophs deposit BIF without biomass, 2014 GSA Annual Meeting Volume 46: Vancouver, British Columbia, Geological Society of America Abstracts with Programs, p. 401.
Tosca, N. J., Guggenheim, S., and Pufahl, P. K., 2016, An authigenic origin for Precambrian greenalite: Implications for iron formation and the chemistry of ancient seawater: Geological Society of America Bulletin, v. 128, no. 3-4, p. 511-530.
Wacey, D., Saunders, M., Roberts, M., Menon, S., Green, L., Kong, C., Culwick, T., Strother, P., and Brasier, M. D., 2014, Enhanced cellular preservation by clay minerals in 1 billion-year-old lakes: Sci Rep, v. 4, p. 5841.
Wagai, R., and Mayer, L. M., 2007, Sorptive stabilization of organic matter in soils by hydrous iron oxides: Geochimica et Cosmochimica Acta, v. 71, no. 1, p. 25-35.
Wang, H., Burgess, R. M., Cantwell, M. G., Portis, L. M., Perron, M. M., Wu, F., and Ho, K. T., 2014, Stability and aggregation of silver and titanium dioxide nanoparticles in seawater: Role of salinity and dissolved organic carbon: Environmental Toxicology and Chemistry, v. 33, no. 5, p. 1023-1029.
Williams, L. B., Canfield, B., Voglesonger, K. M., and Holloway, J. R., 2005, Organic molecules formed in a "primordial womb": Geology, v. 33, no. 11, p. 913.
Zierenberg, R. A., and Shanks, W. C. P., 1988, Isotopic studies of epigenetic features in metalliferous sediment, Atlantis II deep, Red Sea: Canadian Mineralogist, v. 26, p. 737-753.
Review round 2

Reviewer #1 (Remarks to the Author):

R1.1 This is the second time that I have reviewed this manuscript. In my initial review I suggested reject because the authors did not provide compelling evidence to change the currently accepted view that banded iron formations were precipitated as ferric iron via the activity of microbial plankton. Instead, the authors argued that the lack of organic carbon in BIF means that there was limited plankton to start with, and that the subsequent lack of buried biomass then led to limited ferric iron reduction (DIR) and minimal replenishment of nutrients that would have been co-deposited with the iron. Further, the BIF likely have started out as ferrous silicates (i.e., as primary precipitates), thus negating the need to have a biological mechanism to produce the ferric iron
We are grateful to reviewer 1 for providing important comments which have improved the manuscript. We accept the view that ferric iron was part of the initial mineral assemblages in BIF, and we also agree microbial plankton could have been an oxidising agent, although other avenues for iron oxidation are also possible (Cairns-Smith, 1978; Holland, 2006). It is now stated on **lines 342, 368, 380, 383** that our model includes ferric iron being an initial component of BIF, so this should make clear to readers we argue for both ferric and ferrous iron being initially present.
R1.2 The authors have done a significant re-write, largely by moving sections of text

about. They have also politely addressed all of my original points and conceded that there is room for both the traditional BIF models and their own. I agree with that sentiment, but I unfortunately still cannot recommend acceptance of this manuscript because the proof of minimal organic carbon deposition in BIF is lacking. In fact, the rewrite has muddied their model because there are now a number of contradictory statements throughout the text.

We thank the reviewer for appreciation of our previous response; we have replied to the points suggested by the reviewer below. We emphasize here that clays in iron-formations carry the same crystallography (Klein, 2005), and capture OM just like clays in other marine sediments (Ahn et al., 1999; Curry et al., 2007; Dodd et al., 2018; Kennedy et al., 2014; Konhauser and Urrutia, 1999; McMahon et al., 2016). Therefore, clays are archives of OM in marine sediments, our work shows a distinct depletion of OM in clays in BIF, providing evidence of minimal organic carbon deposition in BIF. The latest version of the manuscript includes a description of how plankton may have contributed to iron oxidation (R1.3), the implications of ferric iron and OM in BIF (R.1.5), and why the comparison of BIF and pelite is necessary for comparing OM preservation (R1.6). We also include clarification of the negative feedback model (R1.7) and the model applicability to different types of BIF (R3.4).

R1.3 First, arguing against a ferric iron precursor is fine, but the authors must then better provide a convincing reason to argue against the presence of marine plankton, because if they existed – and overwhelming evidence suggests they did – then they would have oxidized dissolved Fe(II) to Fe(III) oxyhydroxides. Along these lines, the first example of self-contradiction occurs in the abstract where they state that: "As a result of negligible iron reduction nutrients adsorbed to ferric-oxides in BIF would be retained in the sediments and create nutrient-limited waters above, consequently restricting photoferrotrophy, and other metabolisms, thus restraining productivity in the early open oceans". In short, they admit there was plankton and a primary ferric oxyhydroxide but a lack of nutrients from DIR prevented their abundance. Further, the general argument is that the nutrients came onto the shelf via upwelling, a mechanism not addressed in this manuscript.

We agree with the reviewer that BIF initially had ferric iron, we do not argue against a ferric iron precursor (see point R1.1) and our model is entirely consistent with BIF initially having ferric iron. Limited deposition of OM in BIF does not negate ferric iron having been present. OM is believed to reduce ferric iron to magnetite and siderite in BIF, however these minerals can form via other pathways such as ageing of green rust or direct precipitation from seawater or in pore waters (Halevy et al., 2017; Jiang and Tosca, 2019). Therefore, the above statement is not contradictory, we accept photoferrotrophy may have been active and we agree ferric iron was deposited in BIF, which would have scavenged nutrients from seawater, this is stated on **lines 383-393**. The presence of photoferrotrophs does not mean that lots of OM was deposited in BIF, for two reasons: 1) As highlighted in the text on **lines 383,** modern photoferrotorphs separate themselves (OM) from ferric oxides which would produce iron-rich and OM-poor sediments (Emerson and Revsbech, 1994). 2) Ferric iron in BIF may have only been partly sourced from photoferrotrophs, therefore small amounts of OM would be produced, other mechanisms could create ferric iron such as abiotic photo-oxidation (Cairns-Smith, 1978) or direct oxidation by O2 (Holland, 2006).

It is a good idea to discuss the role of upwelling as the reviewer suggests, we have modified the sentence on **line 390** to address upwelling of nutrients.

R1.4 Second, if the authors want to argue for primary ferrous silicate phases, then they must also explain why BIF today have a large ferric iron component.

We see there has been some confusion regarding ferrous vs. ferric iron being the primary minerals of BIF. As mentioned in points R1.1 and R1.3 we accept ferric iron being a primary element in BIF, this does not change our model. We also consider ferrous iron as being a primary element, indicated by petrographic and experimental evidence (Johnson et al., 2018; Konhauser et al., 2007; Rasmussen et al., 2019; Rasmussen et al., 2017; Tosca et al., 2016). We have clarified this by emphasising the primary origin of both ferric and ferrous iron in BIF on **lines**: **245, 318, 342, 368, 371, 380, 383.**

R1.5 Third, the lack of organic carbon in BIF is not proof that the carbon was not there to

begin with. Indeed, the authors in several places in the text point out that there was some carbon burial with BIF. And, as I pointed out in the previous review, even in some of the author's thin sections there is organic matter (OM) preserved in the Dales Gorge BIF samples in close proximity to hematite and magnetite, suggesting the necessary conditions for DIR may have been met.

It is correct that a lack of organic carbon in BIF, is not proof of it initially being absent. That is why we have applied petrographic analysis of organo-minerals in this study. It is possible for organic matter to be oxidised if unprotected (Keil and Mayer, 2014), but BIF contain 20-80wt% clay minerals, which are one of the most potent preservers of organic matter on Earth. Therefore, uniformitarianism would suggest organic matter will be preserved in protective clays, if organic matter was present in abundance. Indeed, organic matter is present in BIF at low concentrations of 0.1 to 0.01wt% and even less, but this does not mean that there was lots of OM to begin with. In fact it would be more strange to find no OM preserved in marine sediments. We also agree that conditions for ferric iron reduction have been met and we show petrographic evidence for this in the form of organic matter associated with carbonate, apatite, and magnetite, which is also explained this way on lines XYZ, and interpreted. However, such conditions would be limited by the initial low supply of OM, as evidence by depletions of OM in clays.

R1.6 Fourth, I still struggle with the comparison of mechanisms in the preservation of organic carbon in shales (with high clay content) to preservation of organic carbon in chemical sediments such as BIF, rocks with low clay content. The authors state in the abstract that: "We find that within shales >80% of OM occurs in clays, but around 0<1% occurs in clays within BIF, suggesting water columns above BIF were starved of OM in BIF clays. However, BIF do not have detrital clays as demonstrated by their low Al content.

The reviewer is correct that shales on average have a higher clay content than BIF, and generally these clays are detrital. As highlighted in the previous revision of this manuscript, BIF still have appreciable amounts of clay 20-80wt% (in samples of this study). While the clays in BIF are believed to be precipitates as opposed to detrital, both detrital clays and precipitates would be suspended in the water column and therefore open to scavenge organic matter. Therefore, it is appropriate organic matter can be found in clays in both sediment types. We can show organic preservation by clays is similar in shales and BIF, because granular iron formations exhibit higher organic carbon contents in clay-rich varieties (Table. 2; (Dodd et al., 2018)), and organic matter is found in clays (Fig. 8; Dodd et al., 2018). We highlight this reasoning on **lines 292-295**.

R1.7 Fifth, as I mentioned in my initial review, I was confused about how the negative feedback works. If limited DIR means less nutrients to the water column, then biomass would decline. So, how did the BIF form unless you want to argue that all BIF started out as ferrous iron-bearing minerals. But you are not consistent in this view throughout the text. Also, as the traditional model goes, DIR in the sediment pile would reduce ferric oxyhydroxides and release previously sorbed elements into the sediment porewaters. But why would we assume that the dissolved elements would diffuse/advect back into the water column versus becoming incorporated into an authigenic mineral phase? Again, this model also assumes the elements for plankton come from the sediment versus upwelling currents.

If biomass declined because of nutrient depletion, BIF formation would indeed slow if biological productivity was the main control on BIF formation. However, biological activity would never cease to exist, so BIF could always be forming but at fluctuating rates due to the negative feedback cycle. Text was added on **lines 396-401** to highlight this.

As we noted in points R1.1 and R1.3 we do accept ferric and ferrous iron were major constituents of primary minerals in BIF. We do not advocate solely for ferric or ferrous-bearing minerals. We have now emphasised this view, where possible in the text (**lines 245, 318, 342, 368, 371, 380, 383**) to avoid this misunderstanding.

Dissolved nutrients indeed may not diffuse out of the sediments, and may be incorporated into authigenic minerals. This is controlled principally by sediment chemistry and redox conditions (Ingall et al., 1993; Tribovillard et al., 2006). BIF have a lot of iron-oxides which would retain nutrients in the

sediment unless these oxides are reduced by organic matter. We argue they were not reduced, and nutrients were retained in the sediments adsorbed to minerals and in authigenic minerals. Upwelling is a major source of nutrients in the modern ocean. Dissolved phosphorus and its diffusion from sediments will contribute to the P inventory of bottom water and therefore the nutrient contents of upwelled waters (Föllmi, 1996). Phosphorus diffusion from sediments accounts for about $1x10^{12}$ mol P/yr in the modern ocean, whereas the total upwelled P is $1.9\ x10^{12}$ mol P/yr (Ruttenberg, 2003), therefore P diffusion from sediments accounts for more than half of the upwelling nutrient inventory. Thus, it is appropriate the model accounts for nutrients from the sediments as a major control on biological activity. To address upwelling of nutrients we included the following statement on **lines 390**: "... insufficient to support widespread reduction of ferric iron, and therefore prevent the return of phosphorus to the water column, leading to upwelled waters being poorer in nutrients."

Minor issues:

R1.8 L65: There are more factors responsible for sorbing organic carbon that just grain size. What about particle surface properties?
This is true. Also, the type of functional groups in the organic matter affects what minerals it bonds to best (Newcomb et al., 2017). We added the following sentence to clarify this: "Other factors such as depositional rate, redox conditions and OM chemistry (such as specific functional groups) also will affect OM preservation in sediments (Keil and Mayer, 2014)."
R1.9 L67-84: Yes, clays may preserve organic carbon but surely not iron oxides. Therefore, stating that: "Organo-minerals can consequently be used to reveal whether the low organic totals of BIF are a result of little to no deposition of OM in BIF, or near complete oxidation of the initial amount" is not actually true.
We agree with the reviewer that clays preserve organic matter, so organic matter should be preserved in clays within BIF if, there was significant quantities of organic matter. However, we found no organic matter in clays in the BIF samples and we have used this observation to argue there was limited initial organic matter in BIF. Independent research has shown iron-oxides also can preserve OM preserve OM (Lalonde et al., 2012; Newcomb et al., 2017). We provide Raman data in Fig. 6 showing OM bonded with the iron oxide hematite. However, it is the use of clays as organo-minerals which best shows that OM was deposited in low quantities in BIF. Therefore, organo-minerals can be used to assess OM preservation. We can show this principle works for iron formations, because granular iron formations exhibit higher organic carbon contents in clay-rich varieties (Table. 2; (Dodd et al., 2018)), and organic matter is found in clays (Fig. 8; Dodd et al., 2018).
R1.10 L189-192: but most BIF did not form in brine pools adjacent to hydrothermal vents. Surely the hydrothermal carbon isotope signature would get diluted in bulk seawater during transport from submarine vents to the shelf.
We agree with the reviewer the carbon isotope signature would be diluted during transport. We used this example to show that hydrothermal activity can modify the carbon isotope signature of seawater. On the early Earth, higher hydrothermally activity may have allowed long transport of ^{13}C-depleted carbon isotope signatures (Beukes and Klein, 1990). However, we have modified the text to include the reviewer's comment about dilution during transport. See **lines 154-158**.
R1.11 L195-198: The broad community would argue that the negative 13C in BIF is caused via DIR, the exact thing the authors here are arguing against.
The reviewer is correct that the ^{13}C values in BIF is commonly thought to be a result of DIR. We accept this view, however as echoed by reviewer 3 during revisions, negative ^{13}C in BIF may likely represent a combined effect of hydrothermal activity and some DIR. To make clear that we consider this an important process, we included the following statement:" The isotopic composition of carbon in carbonate from some BIF is lower than -10‰ [3,17,24] (Supplementary Table 3), suggesting average

mantle carbon values might not be solely accountable for the isotopic depletions (Supplementary figure 1)."

R1.12 L291-293: The sentence: "Therefore, the presence of apatite layers containing inclusions of OM in the Dales Gorge BIF (Fig. 5B), and OM in carbonate (Fig. 5F), is compelling evidence for localised oxidation of OM in some BIF layers" seems contradictory. Here the authors argue for organic carbon deposition, but to avoid arguing against much carbon deposition (e.g., their model) they propose that it is localized. There is no proof that it is localized production of organic carbon versus the traditional model that most organic carbon was oxidized.

We acknowledge that organic matter was deposited in BIF, made evident by total organic carbon measurements and Raman imaging or the OM associated with apatite and carbonate. However, these measurements show organic carbon is exceptionally low, and the Raman imaging reported here shows clays do not preserve OM. Clays should have preserved OM if OM was present in large abundance, given clays in iron formation do preserve OM (Dodd et al., 2018). Therefore, it is concluded OM was in short supply, but still present and what little was present would have been oxidised to form apatite or carbonate. We appreciate the reviewer's comment, and agree the word localised is not the proper term to use in this instance. We reworded the sentence to: "Therefore, the presence of apatite layers containing inclusions of OM, magnetite and haematite in the Dales Gorge BIF (Fig. 5B), and OM in carbonate (Fig. 5F), is compelling evidence for oxidation of OM by ferric iron in some BIF layers". We agree that there is evidence for OM oxidation, however this was limited by the limited deposition of OM in BIF, as evidence by their absence in typical clay organo-mineral associations. This is made clear in the conclusions on **lines 401-405**.

R1.13 L319: Importantly the OM with clays is in the chert bands, not the true oxide-bearing BIF. I'm sure the authors are well aware that not all BIF layers formed in the same manner, but as written, the general reader might not.

We agree with the reviewer that BIF layers can be heterogeneous and there may be different varieties of BIF layers, a point also mentioned by reviewer 3. However, the siliceous layers or shale layers are not BIF and are not reported as such, therefore as concluded by our model (Fig. 8), there was high OM deposition in shales and low OM in BIF. Therefore, OM will occur in clays within chert/ shale layers and not in BIF layers. New text was added on **lines 359-362** to clarify to the general reader the heterogeneity of BIF.

R1.14 L372-373: The statement: "The relatively common association of OM with carbonate and apatite, but not Fe-clay layers, in BIF is puzzling" is not all that puzzling if you invoke DIR.

If there was OM in clays and with carbonate and apatite, yes this would be compelling evidence for lots of OM deposition and DIR. However, one must explain why OM does not occur within its typical clay organo-mineral assemblage, as it does in all other sedimentary types. For OM to avoid encapsulation with clays, the clays would need to be late-diagenetic minerals, which is contradictory with the view that clays are marine precipitates or early diagenetic clays (Konhauser et al., 2007; Rasmussen et al., 2017; Tosca et al., 2016). Therefore we favour a depositional system with low OM concentrations.

R1.15 L396-398: The authors have to watch selectively citing papers that support their model while ignoring others. An example is: "Experimental observations however show amorphous Fe-silicates readily form within Precambrian seawater solutions 31,32,47,27,28,40, therefore a purely diagenetic origin for Fe-silicates seems unlikely". Yes, there are studies that purport that ferrous silicates could have formed out of Precambrian seawater, but there are also quite a few that would argue the clays are diagenetic – see a recent paper by Isson and Planavsky 2018.

We agree with the reviewer that and we have tried to keep an open debate on the authigenic (marine precipitate) vs. diagenetic origin of Fe-silicates in BIF. This can be seen on **lines 193-197 and 227-259** in the manuscript. We cite literature which argues for a diagenetic origin of Fe-silicates in BIF, as well as those advocating for an authigenic origin. Therefore, we believe we have been fair with the cited literature.

R1.16 L476-479: Here the authors calculate how much organic carbon should have been preserved

in BIF assuming that the shales and BIF had the same clay content. But, they did not, so if you need clays to preserve the carbon then clay-deficient BIF will not preserve organic carbon.

We stress that BIF are not always clay deficient, as described on **lines 303-314** from bulk rock geochemical data and petrographic estimates BIF contain about 5-80wt% (average 20-30%). This calculation used the relative abundance of clay in BIF and shale, and calculated how much TOC should be preserved in BIF, taking into consideration its lower clay content. The calculation does not assume BIF and shale have the same clay content, on the contrary it shows even with lower clay totals in BIF, BIF should have about 20 times more OM that it currently preserves. We have reworded the **lines 310-314** to make clear this distinction.

R1.17 L504-507: This is another example of contradiction. Here the authors state that: "there is a stronger correlation between TOC and ferric iron than TOC and Al in the Transvaal BIF (Supplementary Figure 3A, D), which could imply OM was sourced from regions of ferric iron production". Yes, BIF started out as ferric iron, not ferrous iron, and biomass was responsible for the oxidation reactions.

This statement would indeed be contradictory if we argued that BIF did not have a ferric iron component initially. However, as pointed out in R1.1 and R1.3 we agree BIF had ferric and ferrous iron components initially, this is highlighted in the model (Fig. 8).

R1.18 L509-511: BIF are not deep water as they formed on the shelf. The authors are correct that the BIF basin was starved of detritus, which is why they have low clays – a point I've been making and a reason why shales and BIF must be treated differently.

Thank you for the comment, we have removed the words deep-water and left the line as: "... BIF being deposited in basins which are starved of detritus". However, we emphasise that clays in BIF are precipitates formed by mixing of dissolved iron and silica (Konhauser et al., 2007; Tosca et al., 2016), and are not detrital, therefore their presence does not depend on detrital input.

R1.19 L521-524: Another contradiction because here the authors are in fact arguing for plankton forming BIF, hence carbon being associated with ferric iron. This then leads to DIR which oxidizes the organic carbon and therefore nicely explains why BIF have limited organic carbon.

Studies of modern iron-oxidizing bacteria show that the ferric iron they produce is rarely associated with OM (Boyd and Scott, 2001; Emerson and Moyer, 2002; Emerson and Revsbech, 1994), nor are their fossilized counterparts found with OM (Ayupova et al., 2016; Little et al., 2004). Therefore, we do not suggest it is a contradiction arguing for plankton being involved in BIF formation. Following our points R1.1 and R1.3 we agree plankton could have been involved in iron oxidation, however this doesn't necessitate large quantities of organic matter being deposited with ferric iron as described in R1.1 and R1.3.

R1.20 As I stated in my initial review, the strength of this paper would be in looking at trends in organic matter content of the metapelites but I think the comparison of them with BIF does not work because we are comparing apples and oranges.

We thank the reviewer for the constructive criticism and we agree that BIF and metapelite are different and should be discussed carefully. We have therefore carefully highlighted this on **lines 285-314**. We chose these two rock types because BIF have exceptionally low OM (0.01wt%) and metapelite have >0.5-1wt% OM, and both contain clays, therefore the contrasting TOC preservation of these sediments provides the right opportunity to test whether OM deposition in BIF was high. While the clays in BIF are believed to be precipitates or early diagenetic as opposed to detrital in shales, all these clays would be suspended in the water column or open to capture OM in the sediments and therefore all can trap organic matter. So irrespective of the different chemistry of the rocks, the physical properties and nature of clays is comparable between both rock types. Additionally, we can show organic preservation by clays is similar in shales and BIF, because granular iron formations exhibit higher organic carbon contents in clay-rich varieties (Table. 2; (Dodd et al., 2018)), and organic matter is found in clays in these rocks (Fig. 8; Dodd et al., 2018). This demonstrates organic matter is preserved by similar organo-minerals in both shale and BIF, therefore the comparison of these sediments is valid.

Reviewer #2 (Remarks to the Author):

R2.1 The authors have addressed my concerns, and I am now very happy to recommend the manuscript for publication in Nature Communications with no further revisions.
We are grateful to reviewer 2 for having taken the time to review this manuscript.

Reviewer #3 (Remarks to the Author):

R3.1 I would like to thank the authors for taking the time and effort to address my comments and for using them to improve the manuscript while rebutting others. This kind of constructive discussion is what science is all about. The biggest improvement to this version is the addition of geochemical data and its use in strengthening and adjusting their proposed model. I have some additional comments below that should be addressed. However, these are not, in my opinion, major and mostly relates to the authors addressing whether their model is applicable to BIFs in general, or only certain examples/parts/facies of BIF. I think it is an interesting model that shows enough evidence to warrant publication, but could be misinterpreted or applied incorrectly unless its representativity of and applicability to the wide variety of BIF occurrences on Earth are fleshed out better.
Thank you for taking the time to review the manuscript and the considerate reply. We agree that the review discussion has improved the manuscript. We also agree that BIF are heterogenous and that the model will benefit from further clarification regarding this.
R3.2 The authors have added figure 7 that show a slight correlation in literature between Eu anomalies and the δ13C values in BIF. It is an interesting figure and I agree that it can account for some of the negative δ13C values in BIF. However, they state in their rebuttal to reviewer 1 that in modern vent sites that the δ13C values can go down to -70/00. A lot of the data in figure 7 go well below that, so OM oxidation must have, in at least some examples, played a significant role. The authors should consider to add a comment that their model is likely more applicable to some BIFs and less applicable to others, and add some characteristics in a BIF that would align better with their model. Not all BIFs are alike.
We agree with the reviewer that OM oxidation could have contributed to decreasing the carbon isotope signature of those BIF with $d^{13}C$ values greatly exceeding mantle carbon isotope values. We discuss this on **lines 174-185, 357, 402-403**. We have added a description of BIF characteristics most consistent with our model on **lines 359-375.**
R3.3 There is the possibility that the lack of OM associated with clays in BIF when compared to shale, is because the clays were not there at the time of OM preservation. This comes down to the issue of BIF mineral paragenesis. Some argue all BIF were non-redox ferrous clay precipitates (mostly publications from Rasmussen et al.), which is a hypothesis that is simply not representative of BIFs as a whole and are based on microscopic observations on non-representative samples. The authors make it clear that they do not fully support this and that some ferric precipitates must have formed, which is well considered. However, the authors need to admit that their model will be less applicable in instances where all the observed clay minerals are diagenetic products.
There reasoning in lines 236-240 is not, in my opinion, satisfactory as these diagenetic silicates might very likely post-date OM oxidation. In addition, what about instances where there were no clays at all in the BIF, either primary or secondary? The experiment described by the authors in lines 234-236 actually created the mineralogy observed in many BIFs that contain no Fe-silicates (e.g. Smith et al., 2013). To some extent, they address this by looking at ferric oxyhydroxides as sinks for OM, but the potential pitfalls of the model with regards to interpreted mineral paragenesis need to also be more

clearly mentioned. There is a lot of examples and evidence that many BIFs, or parts of BIFs, do not and never did contain any Fe-silicates.

Reviewer 3 correctly summarises the current thinking on ferrous clays in BIF and agrees with our rational that BIF initially comprised both ferrous and ferric mineral portions. We agree with the reviewer that the timing of clay formation is important, if we are to conclude that they captured OM like clays in shale. We acknowledge this on **lines 235-236**, where we give an account of primary vs. diagenetic origins of ferrous clays, and its implications for our model. We have added a sentence on **line 244** to address the reviewer's comment regarding the Fe-silicate formation post-dating OM oxidation.

If there are no clays in the BIF, such as a pure oxide-facies BIF, this does not affect our model, this just means that there was no Fe-silicates to capture and preserve OM in the BIF. It does not change the model, as it can be inferred OM was in low supply, as evidenced by BIF containing some Fe-silicates. It can be inferred oxide facies BIF would also have had low OM, given mixed oxide-silicate facies BIF with Fe-silicates contain no OM in the silicates (Fig 1D; F; I). The experiment we described on **lines 245-247** showed that with the appropriate conditions and chemicals present, Fe-silicates did not form diagenetically, providing evidence against a diagenetic origin for Fe-silicates. Therefore, to account for the presence of Fe-silicates in BIF another mechanism is responsible i.e. water column precipitation. Yet, even if some Fe-silicates/ clays were diagenetic these could still capture OM, as evidence by modern observations of OM being encapsulated by authigenic/ early diagenetic clays (Konhauser and Urrutia, 1999).

R3.4 BIFs show different mineral facies that can be linked to variations in depositional environments in the BIF (e.g. distal deposition preserves magnetite and hematite; more proximal deposition preserves carbonates; Smith et al., 2013 and the multiple works by Beukes and Klein/Klein and Beukes). This implies that the model proposed by the authors could be more applicable to certain parts within a BIF depositional system and not necessarily the BIF as a whole. It would be worthwhile to add comments on the actual mineral heterogeneity linked to stratigraphy in BIFs and how that might influence this model, and how it applies to proximal/distal/shallower/deeper deposition within a BIF and shale system. From what I can ascertain, this model is more applicable to BIF facies where Fe-silicates formed early which, in my opinion, represents only a BIF facies type and not all BIFs.

We agree that the BIF mineral facies are dependent on depositional environments, this is evidenced by bulk rock and trace element chemistry of the various BIF types, exemplified by the papers cited by the reviewer. Proximal deposition preserves iron-rich carbonates with higher TOC and detrital contents than distal magnetite and hematite BIF (Beukes and Klein, 1990; Klein and Beukes, 1989). This is consistent with our model (Fig. 8) which shows higher OM and detrital input in shallower (proximal) sites. The model states that there was low OM deposition in BIF, which limited iron reduction and nutrient release. The key here is OM input and deposition; in our model, distal sites would be further from sites of high productivity and detrital sources, as indicated in Smith et al., 2013 and Beukes and Klein, 1990 and Klein and Beukes, 1989. In these settings, iron reduction would be more limited than proximal BIF which received higher OM input. In summary the reviewer is correct that the model implications will vary depending on depositional site and BIF type. We propose that this is a sliding scale, with the model holding most true for distal BIF which had the lowest OM input. As we gradually move towards proximal sites higher OM deposition would favour more iron reduction and greater release of adsorbed nutrients. This would act to maintain higher biological activity in shallow water/ proximal sites and create nutrient poor open-ocean/ distal conditions, as described in the model (Fig. 8). Please see **lines 359-375** for added discussion on this. The early vs. late formation of Fe-silicates will not affect the model, this would affect the ability of Fe-silicates to preserve OM. The model is only affected by the initial OM deposition. We agree with the reviewer that the ratio of early to late Fe-silicate formation would change depending on the depositional site, and the diagenetic mineral inventory. Proximal BIF with more OM would produce more ferrous iron during diagenesis, which could form more late-stage Fe-silicate.

Refs.

Ahn, J. H., Cho, M., and Buseck, P. R., 1999, Interstratification of carbonaceous material within illite: American Mineralogist, v. 84, p. 1967-1970.

Ayupova, N. R., Maslennikov, V. V., Sadykov, S. A., Maslennikova, S. P., and Danyushevsky, L. V., 2016, Evidence of Biogenic Activity in Quartz-Hematite Rocks of the Urals VMS Deposits, *in* Frank-Kamenetskaya, V. O., Panova, G. E., and Vlasov, Y. D., eds., Biogenic—Abiogenic Interactions in Natural and Anthropogenic Systems: Cham, Springer International Publishing, p. 109-122.

Beukes, N. J., and Klein, C., 1990, Geochemistry and sedimentology of a facies transition from microbanded to granular ironformation in the early Proterozoic Transvaal supergroup, South Africa: Precambrian Research, v. 47, p. 99-139.

Boyd, T. D., and Scott, S. D., 2001, Microbial and hydrothermal aspects of ferric oxyhydroxides and ferrosic hydroxides: the example of Franklin Seamount, Western Woodlark Basin, Papua New Guinea: Geochemical Transactions, v. 2, no. 7, p. 45.

Cairns-Smith, A. G., 1978, Precambrian solution photochemistry, inverse segregation, and banded iron formations: Nature, v. 276, p. 807-808.

Curry, K. J., Bennett, R. H., Mayer, L. M., Curry, A., Abril, M., Biesiot, P. M., and Hulbert, M. H., 2007, Direct visualization of clay microfabric signatures driving organic matter preservation in fine-grained sediment: Geochimica et Cosmochimica Acta, v. 71, no. 7, p. 1709-1720.

Dodd, M. S., Papineau, D., She, Z., Fogel, M. L., Nederbragt, S., and Pirajno, F., 2018, Organic remains in late Palaeoproterozoic granular iron formations and implications for the origin of granules: Precambrian Research, v. 310, p. 133-152.

Emerson, D., and Moyer, C. L., 2002, Neutrophilic Fe-Oxidizing Bacteria Are Abundant at the Loihi Seamount Hydrothermal Vents and Play a Major Role in Fe Oxide Deposition: Applied and Environmental Microbiology, v. 68, no. 6, p. 3085-3093.

Emerson, D., and Revsbech, N. P., 1994, Investigation of an iron-oxidizing microbial mat community located near Aarhus, Denmark: field studies.: Applied Environmental Microbiology, v. 60, p. 4022–4031.

Föllmi, K. B., 1996, The phosphorus cycle, phosphogenesis and marine phosphate-rich deposits: Earth-Science Reviews, v. 40, no. 1, p. 55-124.

Halevy, I., Alesker, M., Schuster, E. M., Popovitz-Biro, R., and Feldman, Y., 2017, A key role for green rust in the Precambrian oceans and the genesis of iron formations: Nature Geoscience, v. 10, no. 2, p. 135-139.

Holland, H. D., 2006, The oxygenation of the atmosphere and oceans: Philosophical Transactions of the Royal Society B: Biological Sciences, v. 361, no. 1470, p. 903-915.

Ingall, E. D., Bustin, R. M., and Van Cappellen, P., 1993, Influence of water column anoxia on the burial and preservation of carbon and phosphorus in marine shales: Geochimica et Cosmochimica Acta, v. 57, no. 2, p. 303-316.

Jiang, C. Z., and Tosca, N. J., 2019, Fe(II)-carbonate precipitation kinetics and the chemistry of anoxic ferruginous seawater: Earth and Planetary Science Letters, v. 506, p. 231-242.

Johnson, J. E., Muhling, J. R., Cosmidis, J., Rasmussen, B., and Templeton, A. S., 2018, Low-Fe(III) Greenalite Was a Primary Mineral From Neoarchean Oceans: Geophysical Research Letters, v. 0, no. 0.

Keil, R. G., and Mayer, L. M., 2014, Mineral Matrices and Organic Matter, p. 337-359.

Kennedy, M. J., Löhr, S. C., Fraser, S. A., and Baruch, E. T., 2014, Direct evidence for organic carbon preservation as clay-organic nanocomposites in a Devonian black shale; from deposition to diagenesis: Earth and Planetary Science Letters, v. 388, no. Supplement C, p. 59-70.

Klein, C., 2005, Some Precambrian banded iron-formations (BIFs) from around the world: Their age, geologic setting, mineralogy, metamorphism, geochemistry, and origins: American

Mineralogist, v. 90, no. 10, p. 1473-1499.
Klein, C., and Beukes, N. J., 1989, Geochemistry and Sedimentology of a Facies Transition from Limestone to Iron-Formation Deposition in the Early Proterozoic Transvaal Supergroup, South-Africa: Economic Geology, v. 84, no. 7, p. 1733-1774.
Konhauser, K. O., Amskold, L., Lalonde, S. V., Posth, N. R., Kappler, A., and Anbar, A., 2007, Decoupling photochemical Fe(II) oxidation from shallow-water BIF deposition: Earth and Planetary Science Letters, v. 258, no. 1-2, p. 87-100.
Konhauser, K. O., and Urrutia, M. M., 1999, Bacterial clay authigenesis: a common biogeochemical process: Chemical Geology, v. 161, p. 399-413.
Lalonde, K., Mucci, A., Ouellet, A., and Gelinas, Y., 2012, Preservation of organic matter in sediments promoted by iron: Nature, v. 483, no. 7388, p. 198-200.
Little, C. T. S., Glynn, S. E. J., and Mills, R. A., 2004, Four-Hundred-and-Ninety-Million-Year Record of Bacteriogenic Iron Oxide Precipitation at Sea-Floor Hydrothermal Vents: Geomicrobiology Journal, v. 21, no. 6, p. 415-429.
McMahon, S., Anderson, R. P., Saupe, E. E., and Briggs, D. E. G., 2016, Experimental evidence that clay inhibits bacterial decomposers: Implications for preservation of organic fossils: Geology, v. 44, no. 10, p. 867-870.
Newcomb, C. J., Qafoku, N. P., Grate, J. W., Bailey, V. L., and De Yoreo, J. J., 2017, Developing a molecular picture of soil organic matter-mineral interactions by quantifying organo-mineral binding: Nat Commun, v. 8, no. 1, p. 396.
Rasmussen, B., Muhling, J. R., and Fischer, W. W., 2019, Evidence from laminated chert in banded iron formations for deposition by gravitational settling of iron-silicate muds: Geology, v. 47, no. 2, p. 167-170.
Rasmussen, B., Muhling, J. R., Suvorova, A., and Krapež, B., 2017, Greenalite precipitation linked to the deposition of banded iron formations downslope from a late Archean carbonate platform: Precambrian Research, v. 290, p. 49-62.
Ruttenberg, K. C., 2003, 8.13 - The Global Phosphorus Cycle, *in* Holland, H. D., and Turekian, K. K., eds., Treatise on Geochemistry: Oxford, Pergamon, p. 585-643.
Tosca, N. J., Guggenheim, S., and Pufahl, P. K., 2016, An authigenic origin for Precambrian greenalite: Implications for iron formation and the chemistry of ancient seawater: Geological Society of America Bulletin, v. 128, no. 3-4, p. 511-530.
Tribovillard, N., Algeo, T. J., Lyons, T., and Riboulleau, A., 2006, Trace metals as paleoredox and paleoproductivity proxies: An update: Chemical Geology, v. 232, no. 1-2, p. 12-32.

Review round 3

Reviewer #3 (Remarks to the Author):

1 I thank the author(s) for once again addressing my comments in great detail. I find the revisions satisfactory and I am happy to recommend publication of this manuscript in Nature Communications.

We are very grateful to reviewer #3 for taking the time to provide this third review and also for assessing our rebuttal to reviewer #1’s comments.

Reviewer #3's assessment of the rebuttal and revisions to Reviewer #1:

This letter serves as my assessment of Reviewer 1’s second round of comments, as well as how they were addressed by the authors (from here on forward I am assuming there were more than one

authors) of the paper titled “Organo-mineral constraints on Earth’s early iron cycle”. I will do this by going through the reviewer’s numbered comments and the authors’ replies to these comments.

2 • R1.1: I am of the opinion that the authors are now providing a balanced view of the different iron formation depositional mechanisms proposed in literature, and that their evidence could point to instances where some previous views need to be challenged or adjusted.

We are glad the reviewer approves of the approach of the manuscript.

3 • R1.2: The reviewer’s comments here are fair with regards to earlier versions of the manuscript. However, I do think the latest version has minimal contradictions. The lack of organic carbon preservation in clay-rich iron formations is a question that needs answering, and the authors make a balanced attempt at that.

We are happy the reviewer approves of the logic of the manuscript and we do not believe that there is any further possible contradictions in this latest version.

4 • R1.3: In my opinion, the latest version of the manuscript does not argue against heterotrophs or ferric iron precipitates thanks to the authors better fleshing out their arguments. Restricting heterotrophy is not the same as stating it was absent. I do understand that the readership might misinterpret this or consider it in extremes, but this will not be the authors’ fault. Also, the authors have scaled back the applicability of their model to all iron formations, and instead apply it to certain depositional settings/iron formation types. The authors are also correct that there are alternative iron oxidation mechanisms.

We thank the reviewer for clarifying this.

5 • R1.4: The authors’ reply to this comment is fair. I, personally, have my reservations about the scientific merits of the cited literature here. However, the literature does suggest that at least some facies in iron formations likely had some primary/early ferrous iron mineral phases.

We thank the reviewer for his/her balanced opinion here.

6 • R1.5: The reviewer is correct that some of the authors’ data support the opposite of what they are arguing. But they also present data in support of their arguments. This could be down to the mineral, textural and depositional heterogeneity of iron formations. It is in the best interest of science that the authors present what appears to be all their data and are not cherry picking, but rather attempting to address their different observations and data. They do ask some hard questions, and their proposed model is feasible for some iron formation occurrences considering their data. I therefore consider their reply to the reviewer here satisfactory.

We thank the reviewer for recognising we have presented a suite of data which best represents the heterogeneity of minerals and textures in BIF and we include citations to other work highlighting the mineral heterogeneity in BIF (Haugaard, Precam Res, 2017; Dodd, EPSL, 2019; Smith, Econ Geol, 2013).

7 • R1.6: Although that there are pitfalls in comparing the mineralogy in different rock types, which I also pointed out in my previous review, the authors’ logic here regarding organic matter preservation in chemically precipitated clays is fair. One would expect a primary clay precipitate to encapsulate and protect at least some organic matter.

We thank the reviewer for their balanced judgment of this reasoning in the manuscript and agreeing with our arguments. The justified comparison of BIF and pelite rock types can be found on lines 303-350. This discussion has been well developed from the reviewer's suggestions through the revision rounds. We have further justified the preservation of organic matter in clays within chemically precipitated rocks, by citing Dodd et al., 2018 which shows clays hosting organic matter in chemically precipitated granular iron formations. We highlight this in reply #12.

8 • R1.7: I can read here the reviewer's concern of proposing a single, blanket iron formation depositional model without the necessary proof. If the authors were proposing an all new and unifying iron formation depositional model, I would also be of opinion that this paper should be rejected. However, the latest version of the manuscript does not read as such and is very carefully considered. I believe the authors' reply to the reviewer here conveys this. I do have some concerns about the abstract of the manuscript though which I will address in my recommendations below.

We thank the reviewer for recognition of our considered model. We previously mentioned in the second response document, and further emphasised in the latest manuscript, we do make the point that there are authigenic mineral phases in BIF that could provide evidence for biomass oxidation, including apatite and carbonate. However, here we document that OM was most likely lower in abundance than previously thought because it is not found in direct association with clays in BIFs, which is an important observation for models of the origin of BIF and early ocean chemistry and productivity. Therefore, we include and consider multiple depositional models and not just one single blanket model. This is made clear on lines 379-388 which includes discussion on our model's applicability to various BIF types, as suggested by reviewer #3 from the previous revision round. We have also addressed the reviewer's comments about the abstract in the replies below.

• R1.8: I am satisfied with the authors' reply here.

9 • R1.9: I am generally satisfied with the authors' reply here. However, their statement "there was limited initial organic matter in BIF" reads too general. This can only be strictly applied to silicate facies BIF, as long as these silicates are primary/early.

We accept the reviewers point here and have rewritten this sentence as follows "Conversely, BIF which possess a primary clay component would be expected to preserve OM in clays, yet this is not seen. This implies OM deposition in silicate-bearing BIF would have been minimal, which would have stifled iron-cycling and primary productivity through the retention of nutrient in sediments.". This newly re-written sentence now highlights that OM and clay minerals can only be used to infer low organic matter burial in those BIF which contain clay (silicate BIF).

• R1.10: I am satisfied with the authors' reply here.

• R1.11: I am satisfied with the authors' reply here.

10 • R1.12: I am satisfied with the authors' reply here. Although I do not fully agree with their interpretation here, their proposed model is not unreasonable considering their data. It does ask some inconvenient questions which will take the research forward.

We are grateful to the reviewer for their balanced assessment of the model and recognition of its value to the field. We revised the manuscript in the previous round following the comments of reviewer #1 from response R1.12 to address the presence of apatite and organic matter in BIF. We noted that apatite occurs in BIF often in layers which can be a result of biomass oxidation. Indeed, as reviewer #3 writes our new observations here asks some inconvenient questions regarding the

origin of this apatite and biomass deposition in BIF. The presence or lack of apatite and OM in BIF may be a result of the varying depositional sites of BIF as we described in the model on lines 379-388 following the suggestion from reviewer #3.

• R1.13: I am satisfied with the authors' reply here.

11 • R1.14: Strictly speaking, one can argue for low organic carbon deposition in silicate facies iron formation, so the authors do take a bit of an extreme view here, which can be scaled back a bit.

We agree with the reviewer's comment here and have highlighted this in the abstract. Please see reply no. 9. As discussed in the previous revision on lines 379-388, the variety of BIF types and their mineral heterogeneity indicate that organic carbon burial in BIF was likely variable depending on the proximity of BIF to shallow marine and productive ocean margins. Therefore, we can suggest in silicate facies BIF - and others with a primary silicate component - that organic carbon burial was low.

• R1.15: I am satisfied with the authors' reply here.
• R1.16: I am satisfied with the authors' reply here.
• R1.17: I am satisfied with the authors' reply here.
• R1.18: I am satisfied with the authors' reply here.
• R1.19: I am satisfied with the authors' reply here.

12 • R1.20: I am partially satisfied with the authors' reply here. However, to directly use granular iron formations in their argument can also have pitfalls, as granular iron formations have a different depositional setting than normal micritic iron formations. The authors should address such potential shortcomings.

The reviewer is correct that granular iron formations had a different depositional setting, however the silicate types in granular iron formations are similar to silicates in micritic iron formations therefore comparison is appropriate. However, we agree with the reviewer that the comparison needs to be clarified. We therefore have reworded this comparison on lines 309-320 as "This principle is also applicable to iron formations, as granular iron formations contain similar clay types (minnesotaite, greenalite, stilpnomelane 46,47) to banded iron formations, and have been found to exhibit higher organic carbon contents in clay-rich varieties 47, and organic matter is found in clays within these clay-rich granular iron formations, as is found in pelites. Therefore, the comparison of OM-clay associations in BIF and pelite is appropriate. However, granular iron formations formed in shallower water settings compared to banded iron formations, therefore depositional mechanics would have been different. Nevertheless, it is unlikely the depth of deposition greatly affects the ability of Fe-clays to bind with OM. Moreover, granular and banded iron formations are both predominantly chemically-precipitated sediments 4,48. Therefore granular iron formations show clays in chemically-precipitated sediments exhibit the same ability for OM preservation as those clays in clastic sediments."

13 Based on the above I would recommend the following minor revisions:
• The abstract is still too one sided and extreme in its statements, especially towards its close. It should be adjusted to be more balanced and accurately convey the contents of the paper. As the abstract reads in the latest version, it highlights many of reviewer 1's concerns, whereas the actual content of the manuscript does less so.

The abstract has been modified to highlight key observations conveyed in the manuscript (presence of OM with apatite and carbonate and clays deficient in organic matter) and discusses both evidence

for the conventional model involving OM oxidation and the new observations for OM depletion. Also we have further balanced the abstract by explaining that our observations are most applicable to silicate-bearing BIF. Please see highlighted revisions in text file and reply number 9. The abstract is then concluded with a brief sentence with the new implications for early ocean chemistry, productivity and the iron cycle.

14 • The use of organic carbon content in granular iron formations in the authors' arguments should be used more carefully, as the granular iron formations have a different depositional setting to deeper water micritic iron formations. The authors should consider any comparative pitfalls.

We have used published observations (Dodd et al., 2018) of clay-rich and clay-poor granular iron formations to show that the clay-rich varieties contain an order of magnitude more organic matter, and that organic matter occurs within clays in granular iron formations. Moreover, the chemistry and structure to those clays in micritic iron formations are almost identical to those clays in granular iron formations (Klein, 2005), therefore, we may expect similar modes of organic preservation in banded iron formations and granular iron formations.

We agree with the reviewer that granular iron formations were deposited in different environments. Therefore, we have carefully reworded comparison with granular iron formations and banded/ micritic iron formations on lines 309-320. Please see reply no. 12 for the new wording used. The new wording highlights that the two sediment types are similar and contain almost identical clay types, although granular iron formations were deposited in shallower environments, however the shallow versus deep setting is unlikely to greatly change the affinity of clays for OM.
